# Membrane sculpting by curved DNA origami scaffolds

**DOI:** 10.1038/s41467-018-03198-9

**Published:** 2018-02-23

**Authors:** Henri G. Franquelim, Alena Khmelinskaia, Jean-Philippe Sobczak, Hendrik Dietz, Petra Schwille

**Affiliations:** 1 Max Planck Institute of Biochemistry, D-82152 Martinsried near Munich, Planegg, Germany; 2 Graduate School of Quantitative Biosciences, Ludwig-Maximilans-University, D-81337 Munich, Germany; 3 Physics Department and Institute of Advanced Study, Technische Universität München, D-85748 Garching near Munich, Germany

## Abstract

Membrane sculpting and transformation is essential for many cellular functions, thus being largely regulated by self-assembling and self-organizing protein coats. Their functionality is often encoded by particular spatial structures. Prominent examples are BAR domain proteins, the ‘banana-like’ shapes of which are thought to aid scaffolding and membrane tubulation. To elucidate whether 3D structure can be uncoupled from other functional features of complex scaffolding proteins, we hereby develop curved DNA origami in various shapes and stacking features, following the presumable design features of BAR proteins, and characterize their ability for membrane binding and transformation. We show that dependent on curvature, membrane affinity and surface density, DNA origami coats can indeed reproduce the activity of membrane-sculpting proteins such as BAR, suggesting exciting perspectives for using them in bottom-up approaches towards minimal biomimetic cellular machineries.

## Abstract (web summary)

BAR domain proteins feature a “banana-like” shape which is thought to aid membrane scaffolding and membrane tubulation. Here authors use DNA origami mimicking BAR domains, giant unilamellar vesicles and fluorescence imaging to study how different BAR domain shapes bind and deform membranes.

## Introduction

The curvatures of biological membranes vary strongly, from predominantly flat in the plasma membrane to highly curved in the endoplasmatic reticulum or in the Golgi apparatus. The transformation of membranes from one shape to another, for example during cell division, belongs to the most fundamental processes in living cells. Numerous factors that regulate membrane curvature have been identified, with scaffolding proteins being the most obvious ones^1–3^. An important class of scaffolding proteins which presumably imprint their shape on lipid membranes is the BAR (Bin/Amphiphysin/Rvs) domain superfamily^4, 5^. When dimerized, BAR proteins form characteristic banana-shaped scaffolds that induce and stabilize membrane curvature through electrostatic and hydrophobic interactions^4–6^. Several BAR proteins were shown to tubulate membranes in vitro^7–10^. BAR proteins presumably rely on their curved shape for their activity: different types of BAR modules adopt folds with different degrees of curvature^4, 5^. By using BAR domains as model proteins^11, 12^, recent studies emphasized the relevance of physical-chemical foundations for membrane bending. From the minimalistic perspective of bottom-up synthetic biology^13, 14^, it is tempting to speculate about the simplest way to induce specific membrane curvatures, and thus engineer a minimal membrane sculpting machinery de novo. The goal of this work is to mimic structural and functional features of BAR domain proteins by rationally designed DNA origami objects (Supplementary Fig. 1), in order to decipher the essential properties of artificial scaffolds for curving lipid membranes.

Programmable self-assembly with DNA origami may be employed to produce a variety of two-dimensional and three-dimensional structures on the nanometer-scale, including objects with custom curvature^15–19^. This molecular toolkit now serves as the starting point for our goal of constructing membrane-sculpting machinery from the bottom-up. DNA origami has been previously employed to create nanoscale channels in lipid membranes^20, 21^ and to guide the assembly of nanoscale lipid compartments^22–25^. In contrast to DNA origami nanocages^24, 25^ that template small liposomes via detergent removal, our designed origami structures act on preexisting cell-sized vesicles, imitating the mechanism of action of protein coats. Subsequently, in this work, we achieve the transformation of membrane shape on much larger scales, reminiscent of deformations observed in cells^2, 3^.

**Fig. 1:**
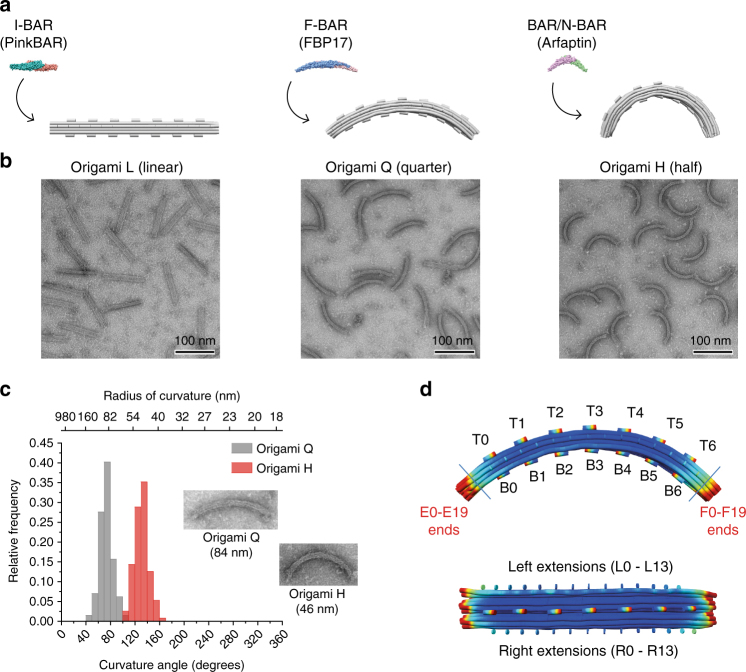
BAR-mimicking DNA origami nanoscaffolds. **a** Structures of origami L (linear), Q (quarter) and H (half), which mimic the shape of I-BAR, F-BAR and BAR/N-BAR domains, respectively. **b** Corresponding negative-stain TEM images of the folded curved nanostructures. **c** The angle of curvature and respective radius of origami structures Q and H (84 and 46 nm, respectively) were experimentally determined from TEM images (*n = *110–130). **d** Schematic representation of marked positions on the top convex (T0–T7), bottom concave (B0–B7), lateral sides (L0–L13, R0–R13) and tips used on the nanoscaffolds (here origami Q) for attaching fluorophores, membrane-anchoring moieties or oligomerizing staples. Scale bars: 100 nm

Taking inspiration from the different degrees of curvature covered by BAR domain proteins, three DNA origami designs (20-helix bundles with hexagonal lattice; Supplementary Figs. 2–4 and Supplementary Tables 1–3) were here developed (Fig. 1): (i) a ‘semi-circle’ named HALF (origami H) with curvature (*C*) ≈ 21.7 μm^−1^; (ii) a ‘quarter-circle’ named QUARTER (origami Q) with *C* ≈ 11.6 μm^−1^ and (iii) a ‘stick’ named LINEAR (origami L) with *C* ≈ 0 (Fig. 1a–c and Supplementary Fig. 5). Despite their fivefold increased length when compared to BAR proteins (∼110 nm vs. ∼ 20 nm, respectively), these origami structures (H, Q and L) mimic the typical shapes of highly-curved BAR/N-BAR dimers, moderately curved F-BAR dimers and flat PinkBAR/I-BAR dimers, respectively (Fig. 1a and Supplementary Fig. 1). Each design further includes positions at the different curved facets for attaching fluorophores, membrane-anchoring moieties or for oligomerizing the objects laterally (Fig. 1d and Supplementary Notes). Thereupon, we studied the interaction of the DNA origami-based scaffolds with lipid model systems and demonstrate the ability for membrane bending in vitro. We determined quantitatively the requirements in terms of shape, membrane-attachment and oligomerization needed for a synthetic scaffold to induce specific membrane curvature. As we explore reconstitution assays with model membranes similar to the ones employed for studying scaffolding proteins in vitro^8–10^, direct comparison with the mechanism of action of BAR domain proteins can be drawn.

Our results demonstrate that DNA nanotechnology has reached the degree of sophistication to reproduce complex biological functionality, which has so far been thought to be reserved for proteins. We show that structure- and function-specific DNA origami devices, biomimetic of proteins targeting and remodeling biological membranes, can be rationally designed and reconstituted into cell-sized model membrane environments. This opens up exciting perspectives for bottom-up synthetic biology approaches, as even more complex fundamental biomimetic nanosystems, such as protein-less membrane trafficking and protocell division machineries, may be within reach.

## Results

### Efficient binding of curved DNA origami to membranes

**Fig. 2:**
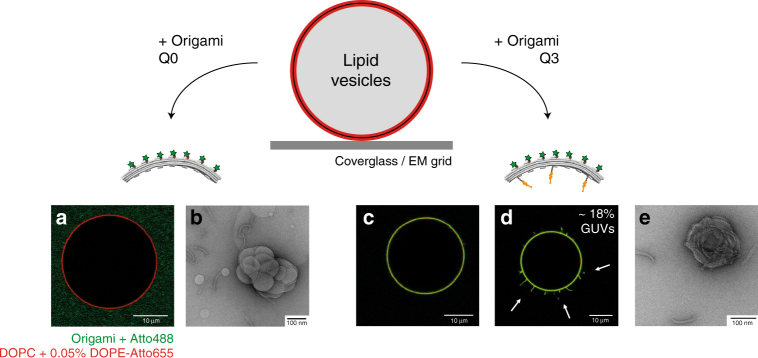
Binding of curved origami structures to lipid model membranes. Interaction of the BAR-mimicking curved origami structures (labeled with Atto488; green) with DOPC model membranes (labeled with DOPE-Atto655; red) assessed using confocal microscopy and TEM. Bare DNA origami nanostructures (Q0) did not interact with GUVs, as observed on GUVs imaged at the equatorial plane by confocal microscopy (**a**) and on MLVs by negative-stain TEM (**b**). Incorporation of three TEG-chol moieties at the distal 5′-end of 18 bps-long linker sequences extending from the origami backbone (structure Q3), rendered optimal binding of the DNA origami structures to lipid bilayers (**c**, **e**). After incubation for at least 1 h with origami structure Q3 (**d**), circa 18 % of the GUVs presented outwards lipidic tubules (marked by arrows). Scale bars: (**b**, **e**) 100 nm; (**a**, **c**, **d**) 10 µm

We assessed the interaction of curved DNA nanostructures with lipid membranes, mainly giant unilamellar vesicles (GUVs) composed of 1,2-dioleoyl-*sn*-glycero-3-phosphocholine (DOPC), via fluorescence confocal microscopy (Fig. 2). Incorporation of 7 × Atto488-modified staples on positions T0-6 enabled fluorescence detection of the origami structures (Fig. 1d). Similarly to what was described elsewhere^26^, bare DNA origami structures lacking membrane anchors were adsorbing to lipid bilayers in the presence of 20 mM MgCl_2_ (Supplementary Fig. 6). We avoided such unspecific membrane attachment (Supplementary Fig. 7) and ensured long-term stability of the nanostructures^27^ with an imaging buffer containing 5 mM MgCl_2_ and 300 mM NaCl in which the Na^+^ outcompetes membrane-adsorbed divalent cations via a counterion release mechanism to break up Mg^2+^ promoted interactions between DNA and the phospholipids^28, 29^. To achieve side-specific binding of the curved DNA origami structures to lipid membranes, we tested various methods including neutravidin-mediated attachment^21, 30^ of biotinylated origami H to biotinylated lipids (Supplementary Fig. 8a) and covalent attachment^24, 31^ of thiolated origami H to maleimide-modified lipids (Supplementary Fig. 8b). However, preferred membrane anchors were oligonucleotides linked to a cholesteryl moiety via a tetraethylene glycol spacer (TEG-chol), as they have been already extensively characterized^32^ and allowed for a steady binding of nanostructure H to lipid bilayers (Supplementary Fig. 9) in comparison to the other approaches.

In order to enhance attachment of curved DNA origami scaffolds to model membranes and avoid steric hindrance (Supplementary Figs. 10–12), we placed TEG-chol moieties at the distant 5′-end of 18 bps-long linker sequences extending from the origami backbone (anchor orientation called TC5). Placing the anchors closer to the origami backbone, i.e., at the proximal 3′-end of the linker sequences (Supplementary Fig. 10a and Supplementary Fig. 11d–f), or shortening the linker length from 18 to 9 bps (Supplementary Fig. 12b, d), severely reduced binding of nanostructures H and Q to membranes. This effect was particularly prevalent for membrane binding through the concave origami surface.

Since anchor accessibility plays a decisive role for attaching DNA origami structures to lipid membranes^33^, we further evaluated how number and positioning of TC5 anchors along the concave origami facet may influence binding of nanostructures H and Q to GUVs. When single TC5 anchors were introduced (Supplementary Fig. 13b–d and h–j), no significant attachment of our curved nanoscaffolds to membranes was observed (Supplementary Fig. 14). In contrast, when three TC5 anchors were incorporated (Supplementary Fig. 13e–f and k–l), membrane affinity was significantly increased, especially if the anchors were placed at positions B0, B3 and B6 (combination from here on called *X*3) (Fig. 2c and Supplementary Fig. 13f, l). Using negative-stain TEM imaging, we further corroborated the attachment of construct Q3 to lipid vesicles (Fig. 2e).

Taken together, we identified some of the major requirements for efficiently attaching curved origami structures to lipid bilayers and we observed indications of curvature-mediated deformations of membranes induced by the F-BAR mimicking structure Q3 (Fig. 2d and Supplementary Fig. 13m). Notably, incubation of GUVs with Q3 for at least 1 h led to the appearance of outwards tubular deformations within a significant fraction of vesicles (~18%; 22 out of 121 GUVs); deformations similar to the positively curved tubules reported for several F-BAR proteins^7, 8, 10, 34^.

### Membrane deformations as a function of DNA origami curvature

As different classes of BAR domains curve membranes in distinct manners^4^, we further investigated whether the appearance of membrane deformations, as reported in Fig. 2d, can be correlated with the direction and degree of curvature of our BAR-mimicking DNA origami structures. Membrane tension has been previously implicated in influencing the assembly of BAR domain proteins^35, 36^. To provide a controllable trigger for assessing vesicle deformations, we lowered the membrane tension by increasing osmolarity of the outer buffer in 10%. Subsequently, shape variations of the deflated GUVs were monitored.

**Fig. 3:**
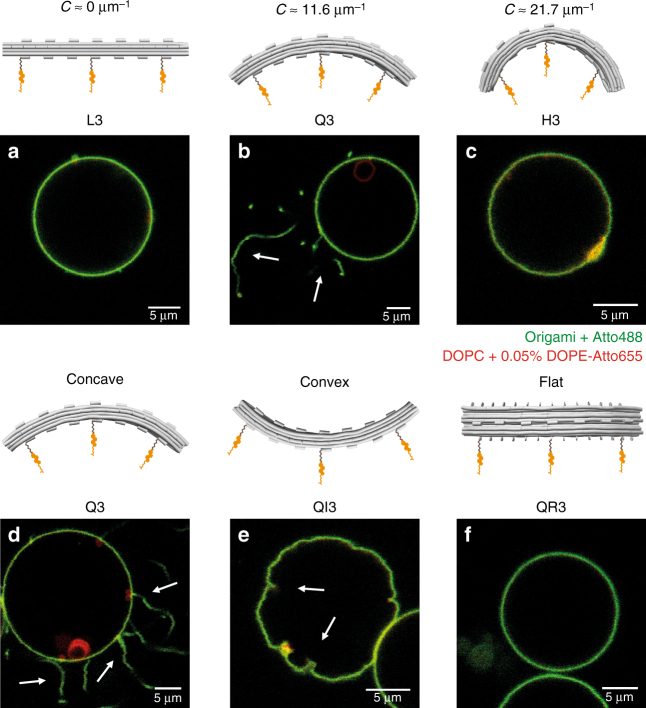
Triggering of membrane deformations depends on the degree of curvature of the BAR-mimicking DNA nanoscaffolds. DNA origami structures (labeled with Atto488; green) of varying curvature (L3, Q3 and H3; here at 5 nM bulk concentration) were incubated with DOPC GUVs (labeled with DOPE-Atto655; red) for at least 1 h. After membrane binding was achieved, the surface tension of the GUVs was lowered by applying a hyperosmotic stress (10% increase in buffer osmolarity) and consequent changes in vesicle shape were monitored. No significant changes in vesicle shape were observed with membrane-bound origami L3 (**a**) and H3 (**c**). Vesicles covered with the moderately curved structure Q3 presented long tubular outward structures upon hyperosmotic stress (**b**; marked by arrows). Similarly, membrane interaction of origami Q variants displaying three cholesteryl anchors on different curved facets was further investigated. Strong binding to GUVs was achieved for all nanostructures, independently of the facet where anchors are localized (**d**–**f**). Upon vesicle deflation, the concave structure (Q3) triggered outwards membrane tubules (**d**; marked by arrows); the convex structure (QI3) triggered evagination/invagination-type of deformation (**e**; marked by arrows); and the structure with null curvature (QR3) led to no significant changes in vesicle shape (**f**). Scale bars: 5 µm

After the hyperosmotic stress, lipid vesicles without membrane-bound DNA origami (no origami in solution or incubated with nanostructures lacking cholesteryl anchors) rapidly regained their spherical shape, suffering only minor shrinkage or blebbing (Supplementary Fig. 16h–j and Supplementary Movie 1). Bursting events were seldom (~13%; 5 out of 40 GUVs). For vesicles incubated with a structure lacking curvature (L3), a comparable effect was observed (Fig. 3a and Supplementary Fig. 15i, j), independently of the total DNA origami concentration.

Remarkably, moderately curved origami quarter-circles (Q) displaying a concave membrane-binding surface were able to trigger tubulation of GUVs upon hyperosmotic shock (Fig. 3b and Supplementary Movie 2). As seen for structure Q3, this process depended on the total origami concentration. At Q3 concentrations ≤3 nM, most vesicles presented no significant deformations, with only a minor fraction (~18%) displaying outwards tubules (43 out of 244 GUVs). By contrast, at Q3 concentrations ≥5 nM, ~70% of all GUVs (128 out of 189) displayed outwards tubules (Supplementary Fig. 19a–d and Supplementary Fig. 15k–m). If additional cholesteryl moieties were used on the concave surface (as observed for Q7 with 7 × TC5), due to the increase in hydrophobicity and consequently enhanced membrane binding, lower total concentrations of DNA origami were able to induce membrane tubulation (Supplementary Fig. 19i–l and Supplementary Fig. 17g–i). Indeed, most vesicles incubated with Q7 (97 out of 191 GUVs) displayed tubular deformations at concentrations ≥2 nM.

In the same way, we investigated origami Q constructs displaying either a convex membrane-binding interface (QI3; anchors at top positions T0, T3, and T6), or a flat membrane-binding interface perpendicular to the curvature (QR3; anchors at lateral positions R0, R6, and R12). For the latter (QR3), no significant membrane deformations were observed upon osmotic trigger (Fig. 3f; Supplementary Fig. 16m, n and Supplementary Movie 3), consistent with the results reported for the non-curved structure L3 (Fig. 3a and Supplementary Fig. 15i, j). For structure QI3 with convex membrane-binding interface, on the other hand, ~60% of the vesicles (55 out of 92 GUVs; at 5 nM QI3) presented evagination/invagination-type shallow deformations upon hyperosmotic stress (Fig. 3e; Supplementary Fig. 16k–l and Supplementary Movie 4). Such deformations effectively contrasted with the outward tubules observed for structure Q3 (Fig. 3b, d) and were somewhat reminiscent of the negatively curved membrane deformations reported for inverted I-BAR domains^37, 38^.

Contrary to the origami Q structures, the more curved origami half-circle (H) structures with concave membrane-binding interface were not capable of inducing the formation of tubular deformation on GUVs (Fig. 3c): neither at high H3 total concentrations (Supplementary Fig. 15n–p), nor for H7 displaying enhanced membrane binding (Supplementary Fig. 17j–l). For instance, with the exception of seldom vesicle bursting (~15%; 20 out of 134 GUVs) and minor flaccid deformations (~10%; 14 out of 134 GUVs), vesicles incubated with H3 (~75%; 100 out of 134 GUVs) remained spherical upon osmotic change and did not display any tubular deformations. Adsorption of these highly curved origami structures to DOPC vesicles seemed therefore insufficient to overcome the energetic barrier required for bending a flat membrane into a positively curved tube. Indeed, assuming a typical bending modulus for lipid bilayers of ~10^−19^ J, the estimated energy cost for bending a flat membrane segment of surface area (*A*) ~1800 nm^2^ (corresponding to the surface area of our DNA origami scaffolds) into a membrane tube with *R* ≈ 46 nm (radius of curvature fitting origami H) is ~38 *k*_B_*T*, based on the area-difference elasticity (ADE) model of membrane bending^39, 40^ (equation 3). For origami Q (*R* ≈ 84 nm), however, the estimated membrane bending cost is ∼11 *k*_B_*T*, ~3.5-fold lower than structure H and comparable with the membrane bending costs expected for a BAR domain protein (amphiphysin: 9 *k*_B_*T*, for *R* ≈ 11 nm and *A* ≈ 23 nm^2^)^41^.

Taken together, our data show a clear connection between the curvature of the membrane-binding interface of our BAR-mimicking DNA-based scaffolds and the resulting membrane deformations (e.g. tubulation, invagination, etc.).

### Hierarchical oligomerization of curved DNA origami scaffolds

Self-assembly of membrane scaffolding proteins into higher-order structures was suggested to play an important role in the mechanism of action of BAR domains^7, 10^. Both lateral and tip-to-tip linear intermolecular interactions were described to stabilize their assembly into protein lattices^7, 10^. To test the influence of such higher-order linkages, we designed variants of curved DNA origami Q that could oligomerize, similar to BAR proteins, tip-to-tip (Supplementary Fig. 18b–d) and laterally (Supplementary Fig. 18e). Overall, four constructs capable of multimerizing in solution were created: origami Q-E5 (Supplementary Fig. 18b), Q-E7 (Supplementary Fig. 18c) and Q-E13 (Supplementary Fig. 18d) able to linearly multimerize from the tips forming arc-like oligomers of tunable size; plus origami Q-S14 (Supplementary Fig. 18e) able to multimerize laterally forming sheet-like oligomers. Constructs Q-E5/7/13 possess 2 × 5, 7 and 13 blunt ends at defined helices, enabling intermolecular stacking at the origami tips. Construct Q-S14, on the other hand, displays 2 × 14 TATATA overhangs, enabling complementary lateral interactions along the origami sides.

Subsequently, we tested whether the inclusion of those polymerizing staples would enhance the ability of origami Q3 with concave membrane-binding interface to produce tubular membrane deformations on GUVs upon deflation. Altogether, no significant differences in terms of total bulk concentration required to induce tubulation of vesicles were observed for constructs with or without tip-to-tip oligomerizing staples (i.e., structures Q3-E5/7/13 vs. Q3, respectively; Supplementary Fig. 18g–i and Supplementary Fig. 20). In contrast, for the construct with lateral oligomerizing staples (Q3-S14), lower bulk concentrations were required for inducing membrane tubulation upon osmotic stress (Supplementary Fig. 19e–h and Supplementary Fig 18j). Indeed, ~70% of the vesicles incubated with Q3-S14 presented tubular deformations at concentrations ≥3 nM (135 out of 193 GUVs). Likewise, inclusion of lateral polymerizing overhangs on origami QI3 with convex membrane-binding interface (i.e., QI3-S14) also affected the generation of membrane deformations (Supplementary Fig. 21). Here while most vesicles displayed evagination-type membrane deformations upon hyperosmotic stress (Supplementary Fig. 21e, f), ~15% of vesicles (36 out of 244 GUVs; at 5 nM QI3-S14) additionally presented inward tubules (Supplementary Fig. 21d, g) resembling protruding nanotubes described for convex I-BAR proteins^42, 43^; which could not be observed for the structure QI3 lacking lateral overhangs. Incubation with lower bulk concentrations of QI3-S14 (i.e., 2 nM), on the other hand, did not promote significant membrane deformations, similar to what was observed for convex structure Q-I3 lacking polymerizing overhangs (Supplementary Fig. 16k).

In summary, our data indicate that in particular the presence of lateral interactions influences the ability of curved membrane-bound DNA origami to deform membranes. However, this effect seems to be of minor significance, as structures having additional membrane anchors but lacking polymerization strands (i.e., Q7; Supplementary Fig. 19i–l), were able to deform lipid vesicles as efficiently (in terms of total origami concentrations required) as the structures with polymerization strands (i.e., Q3-S14).

### Membrane density and binding affinity of curved DNA origami

Our results so far strongly suggest that a critical membrane density of curved nanostructures is required for triggering membrane bending. To test this hypothesis, variable surface densities of our BAR-mimicking DNA-based scaffolds to DOPC GUVs were quantitatively investigated at equilibrium (after overnight incubation), by fluorescence imaging and single molecule detection.

**Fig. 4:**
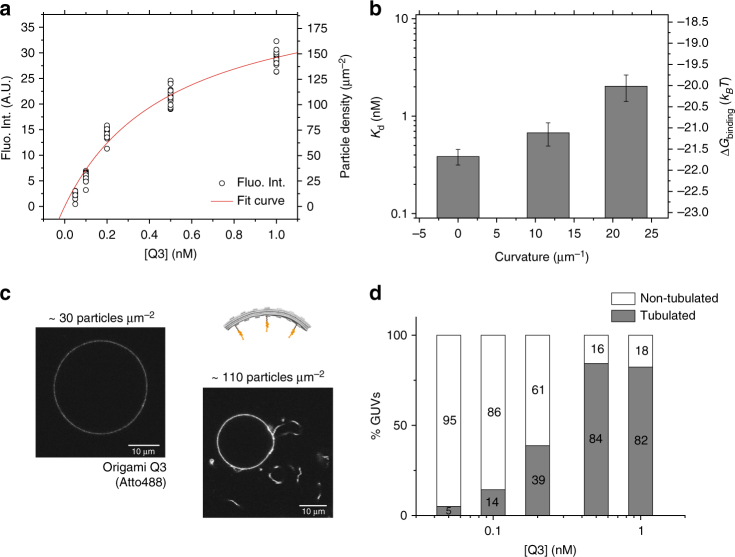
Tubulation of flat membranes depends on surface density of membrane-bound curved origami Q. Fluorescence intensities of membrane-bound DNA origami (labeled with 3 × Atto-488 dyes) at equilibrium (incubated overnight) were extracted using image analysis and represented as a function of total bulk concentration (**a**; here depicted for one independent set of measurements with Q3). Representative confocal images at the equatorial plane for membrane-bound Q3 nanostructures are depicted in **c**. Membrane binding of the DNA nanostructures was quantitatively investigated by fitting the data to a Langmuir isotherm (equation 1), enabling the determination of apparent membrane dissociation constants *K*_d_ ( ± s.d.): L3 (*n*_total_ = 288 GUVs; *n* = 131–157 GUVs per fit, 2 repeats), Q3 (*n*_total_ = 277 GUVs; *n* = 83–100 GUVs per fit, 3 repeats) and H3 (*n*_total_ = 106 GUVs; *n* = 48–58 GUVs per fit, 2 repeats) (**b**). Δ*G*_binding_ was calculated via Δ*G* = *RT*ln*K*_d_. Regarding efficiencies of membrane tubulation (**d**), high yields ( > 80%) were retrieved for Q3 bulk concentrations ≥ 0.5 nM, or, upon conversion to surface densities, for ≥90 membrane-bound DNA origami particles per μm^2^ (as illustrated in **c**). Scale bars: 10 µm. Error bars in **b** correspond to s.d.

Apparent membrane dissociation constants at equilibrium (*K*_d_ ± s.d.) were obtained for L3, Q3, and H3 structures by fitting the fluorescence intensity values on the surface of GUVs^44^ as a function of bulk concentration to a Langmuir isotherm (equation 1): *K*_d_ (L3) = 0.39 ± 0.07 nM (*n*_total_ = 288 GUVs; *n* = 131–157 GUVs per fit, 2 repeats), *K*_d_ (Q3) = 0.68 ± 0.18 nM (*n*_total_ = 277 GUVs; *n* = 83–100 GUVs per fit, 3 repeats) and *K*_d_ (H3) = 2.0 ± 0.6 nM (*n*_total_ = 106 GUVs; *n* = 48–58 GUVs per fit, 2 repeats). Thus, for the same combination of cholesteryl anchors, increasing curvature of the DNA nanoscaffolds from flat (*C* ≈ 0) to highly curved (*C* ≈ 21.7 μm^−1^) prompted a fivefold weaker binding to flat freestanding membranes (Fig. 4b and Supplementary Fig. 24).

By image analysis, we further quantified the efficiencies of vesicle tubulation by the curved DNA origami Q3 nanostructures (Fig. 4d; *n*_total_ = 445 GUVs, *n* = 78–108 GUVs per origami concentration). When compared to the results obtained at a shorter incubation period (Fig. 2c, d and Fig. 3), after overnight incubation lower origami bulk concentrations and no additional osmotic perturbation were required to achieve high yields of membrane tubulation. At equilibrium, >80% of vesicles (154 out of 185 GUVs) presented tubular deformation for Q3 bulk concentrations ≥0.5 nM (value close to *K*_d_). Despite slightly increased membrane affinities (lower *K*_d_ values; Supplementary Fig. 24) when compared to structure Q3, structures with increased numbers of anchors (Q7) or with polymerizing overhangs (Q3–S14) yielded similar membrane tubulation efficiencies (Supplementary Fig. 25).

As the number of fluorescent particles is proportional to the fluorescence intensity, we performed additional FCS measurements in order to calibrate the measured fluorescence values and recover the corresponding densities of membrane-bound DNA origami at the surface of GUVs^44^ (see calibration curve in Supplementary Fig. 22). Considering the average fluorescence intensities of single DNA origami structures, for Q3 with moderate curvature, we estimated 50 ± 20 particles per μm^2^ bound to GUVs (*n* = 51) to be sufficient for initiating tubulation, and 90 ± 20 particles per μm^2^ (*n* = 50 GUVs) for almost all vesicles (>80%) to present tubules (representative curve depicted in Fig. 4a and confocal images in Fig. 4c). At these surface densities, our curved nanoscaffolds cover 9–16 % of the total membrane surface area. Interestingly, this surface fraction matches the previously reported coverage required for BAR domains to induce membrane deformations on model membranes (2–4× higher than for amphiphysin^9^). Flat structure L and highly curved structure H, on the contrary, were not capable of inducing membrane tubulation on GUVs even at surface coverages ≥100 particles per µm^2^ (Supplementary Fig. 23), promoting at best flaccid membrane deformations analogous to the non-spherical shapes previously reported for flat PinkBAR domains^45^. For structure L3, due to its ‘zero’ curvature, no tubulation was to be expected. For highly curved structures H3 and H7, a simple energetic cost-benefit analysis estimates the apparent free energies of membrane adhesion (Δ*G = RT*ln*K*_d_, –20 *k*_B_*T* and –21.5 *k*_B_*T*, respectively) to be clearly insufficient to allow for membrane bending (38 *k*_B_*T*). For the moderately curved origami structure Q3, to the contrary, membrane adhesion (–21.1 *k*_B_*T*) is strong enough to compensate for the energetic cost of membrane bending (11 *k*_B_*T*), hence enabling tubular deformations to be generated.

**Fig. 5:**
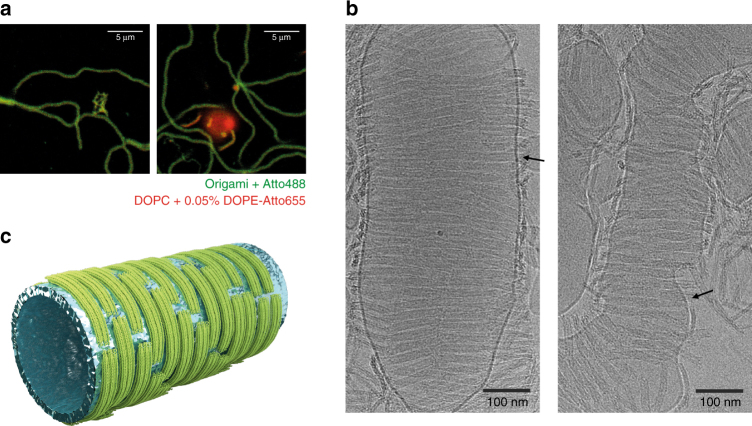
Ultrastructure of lipid nanotubes decorated with DNA origami Q. **a** From confocal images, the membrane tubules obtained from GUVs (labeled with DOPE-Atto655; red) upon overnight incubation with structure Q3 (labeled with Atto488) appeared homogeneously covered with membrane-bound DNA origami. **b** Further cryo-EM imaging confirmed that the surface of the membrane tubules (black arrows) is densely covered with curved DNA nanostructures perpendicularly aligned along the long axis. **c** Based on the cryo-EM electron microscopy observations and radius of curvature of nanostructure Q3, a schematic representation of a lipid nanotube decorated with DNA origami Q is here depicted. Scale bars: (**a**) 5 µm; (**b**)100 nm

Finally, we investigated the ultrastructure of membrane tubules decorated with origami Q3 at high surface densities (i.e., after overnight incubation of GUVs with 5 nM Q3), using cryo-electron microscopy (cryo-EM). From the confocal images (Fig. 5a), the grown tubules appeared homogenously covered with membrane-bound fluorescently labeled Q3. Further cryo-EM imaging (Fig. 5b) confirmed that the surface of the membrane tubules was densely covered with DNA nanostructures, preferentially aligning perpendicularly to the long axis of the tubular structures. Additionally, the recovered tubular diameter (220 ± 70 nm; *n*_total_ = 35 parallel cross-sections, 4 membrane tubules) was in good agreement with the predictions based on the objects curvature (~170 nm; Fig. 1a–c).

## Discussion

This work demonstrates that curvature generation and topological transformation of biological membranes, as required for many cellular functions, can be achieved in a well-controlled fashion by curved synthetic scaffolds made of DNA. The action of these scaffolds may be tuned by varying shape, density, membrane affinity, and the propensity for self-assembly of the scaffolds on membrane surfaces. In contrast to earlier work exploring the deformation of membranes by flat nanostructures^46, 47^, concerted lateral oligomerization by self-assembly plays only a minor role for the specific membrane transforming activity by curved DNA-based scaffolds. Moreover, in spite of producing larger tubular deformations than BAR domains, our curved structures operate at similar membrane bending energy levels. We have established three main requirements for the induction of tubular membrane deformations (Fig. 5c) by scaffolding elements: curvature, membrane affinity and surface density. Remarkably, we provide direct proof that the curvature of membrane associating macromolecular objects plays a decisive role, helping us understand the minimal physical–chemical laws underlying membrane deformations.

In this manuscript, we validate the usage of custom-designed DNA origami as a tool to overcome the limited predictability of engineered proteins. The ability of our developed structures to precisely control local membrane curvature will have great impact in the investigation of all kinds of biological membrane shaping phenomena. For example, sequential binding of proteins involved in deformation cascades (e.g., clathrin-mediated endocytosis^48^, FtsZ-mediated bacterial division^49^) depends on the degree of curvature locally displayed by membranes. In this regard, BAR-mimicking DNA origami scaffoldings could allow detailed investigation of such proteins on model membranes or even cells^50^, as a function of local curvature.

Altogether, our work has great significance for the growing field of bionanoengineering, opening up an avenue of research in synthetic biology. Our present achievements add exciting perspectives towards minimal biomimetic cellular machineries, involved in membrane shaping and beyond; pushing the limits of nanotechnology into cellular biology. As we laid down new foundations on manipulating DNA origami in lipid environments, design of even more elaborate DNA origami supramolecular assemblies targeting lipid membranes (e.g., artificial clathrin coated pits, enzymatic membrane complexes), and novel approaches for developing hybrid DNA-lipid-based drug delivery vehicles directed towards biological membrane barriers, will hence likely emerge in the near-future.

## Methods

### Materials

1,2-dioleoyl-*sn*-glycero-3-phosphocholine (DOPC), 1,2-distearoyl-*sn*-glycero-3-phosphocholine (DSPC), cholesterol from ovine wool, 1,2-dioleoyl-*sn*-glycero-3-phosphoethanolamine-*N*-[4-(*p*-maleimidophenyl)butyramide] (MPB-DOPE) and 1,2-dioleoyl-*sn*-glycero-3-phosphoethanolamine-*N*-(cap biotinyl) (Biotin-DOPE) were purchased from Avanti Polar Lipids (Alabaster, AL, USA). Atto655-DOPE was acquired from AttoTEC GmbH (Siegen, Germany) and DiIC18(5) (DiD) from Thermo Fischer Scientifics (Waltham, MA, USA). Single-stranded M13mp18 scaffold plasmid (p7249) was supplied from Bayou Biolabs (Metairie, LA, USA), as well prepared by Florian Praetorius using high-cell-density fermentation of *Escherichia coli* in stirred-tank bioreactors according to reference^51^. High purity salt free (HPSF) purified staple oligonucleotides for origami preparation, as well as 5′-Atto488, 5′-Alexa488 and 3′Biotin-TEG-functionalized oligonucleotides (all HLPC-purified) were purchased from Eurofins Genomics (Ebersberg, Germany). 5′/3′-Chol-TEG and 3′-Thiol-Modifier-C3 S-S-functionalized oligonucleotides (all HPLC purified) were acquired from Sigma-Aldrich (Taufkirchen, Germany).

### Design and production of the DNA origami nanoscaffolds

The DNA origami structures employed throughout this work consisted in a 20-helix bundle with hexagonal lattice. As described in the main text, three curved designs were here developed: origami H (curvature *C* ≈ 21.7 μm^−1^; curvature angle *θ* ≈ 131°; radius of curvature *R* ≈ 46 nm; Supplementary Fig. 4; Supplementary Table 3), origami Q (*C* ≈ 11.6 μm^−1^; *θ* ≈ 73°; *R* ≈ 84 nm; Supplementary Fig. 3; Supplementary Table 3) and origami L (*C* ≈ 0; Supplementary Fig. 2; Supplementary Table 1). Those structures were based on the M13 p7249 plasmid and designed using CaDNAno^52^ (Supplementary Figs. 2–4). Initial 3D models (Supplementary Fig. 1) were predicted using CanDo^17, 19^. Each design further included marked positions for attaching fluorophores, membrane-anchoring moieties or oligomerizing staples (Fig. 1d). More precisely, 7 sites on the bottom (concave) and top (convex) facets of the DNA origami (B0-B6 and T0-T6, respectively), plus 14 sites on the left and right facets (L0-L13 and R0-R13, respectively) were defined. This strategy allowed us to manipulate the functionality of the origami structures by exchanging the staple sequences at those defined external positions with functionalized counterparts (Supplementary Notes), without compromising the shape of the nanostructures stabilized by the core staples. The edges of each of the 20 helical bundles, usually kept as single-stranded segments to avoid blunt end interactions, could be similarly hybridized with functionalized staples. Folding of all the DNA origami structures was performed in a one-pot reaction mix^33^. Briefly, 20 nM p7249 plasmid and 200 nM staple oligonucleotides were mixed in a 5 mM Tris-HCl, 1 mM EDTA, 20 mM MgCl_2_, pH 8.0 buffer (folding buffer). Thermal annealing was performed over a cooling cycle scheme from 65 to 60 °C in 1 h and from 59 to 40 °C in 40 h, on a Eppendorf Mastercycle Pro (Hamburg, Germany) or Bio-Rad Tetrad 2 (München, Germany) thermal cycler. Purification of the folded structures from the excess of staple strands was performed using size-exclusion centrifugal filtration with Amicon Ultra 100 kDa MWCO filters (Merck Millipore, Darmstadt, Germany) or PEG precipitation^53^ using a buffer consisting of 5 mM Tris-HCl, 1 mM EDTA, 5 mM MgCl_2_, 300 mM NaCl, pH 8.0 (imaging buffer). Bulk concentrations of DNA origami were determined via fluorescence spectroscopy using a Jasco FP-8500 spectrofluorometer (Tokyo, Japan)^33^. Correct assembly of the folded nanostructures was evaluated by agarose gel electrophoresis^17, 33^ (Supplementary Fig. 5), negative-stain transmission electron microscopy (TEM)^17, 54^ (Fig. 1b and Supplementary Fig. 5) and atomic force microscopy (AFM)^33^ (Supplementary Fig. 5).

### Preparation of lipid membranes for fluorescence microscopy

Supported lipid bilayers (SLBs) were obtained via fusion of small unilamellar vesicles deposited on top of freshly cleaved mica, as described elsewhere^55^. Giant unilamellar vesicles (GUVs), the preferred membrane model system utilized throughout this work, were produced by electroformation in PTFE chambers with Pt electrodes^33, 56^. Six microliter of lipid mixture (2 mg mL^−1^ in chloroform) was spread onto two Pt wires and dried in a desiccator for 30 min. The chamber was filled with 350 μL of an aqueous solution of sucrose. An AC electric field of 2 V (RMS) was applied at a frequency of 10 Hz for 1.5 h, followed by 2 Hz for 0.75 h. Unless otherwise stated, vesicles composed of DOPC, containing additional 0.005 mol% (for FCS experiments) or 0.05 mol% (for confocal imaging) Atto655-DOPE, were electroformed in an aqueous solution of sucrose iso-osmolar compared to imaging buffer (~ 575 mOsm kg^−1^). Experiments were carried out in 40 µL MatriCal 384-multiwell plates with # 1.5 glass bottom thickness (Brooks Life Science Systems, Spokane, WA, USA). Prior usage, wells were freshly plasma cleaned, then passivated with bovine serum albumin (Sigma-Aldrich) or PLL(20)-g[3.5]-PEG(2) (SuSoS AG, Dübendorf, Switzerland). Typically, 3 µL of the GUV suspension (pre-diluted at least 1:10 in iso-osmolar sucrose solution) were mixed with 18 µL DNA origami solution at a final 0.5–10 nM concentration diluted in imaging buffer. Unless otherwise stated, samples were incubated for at least 1 h at room temperature. Hyperosmotic stress of GUVs incubated with DNA origami structures was achieved by gently adding 3 µL of a glucose solution diluted in imaging buffer (1000 mOsm kg^−1^) into the imaging chambers.

Typically, at least two independent sets of measurements were performed for evaluating a specific experimental condition under confocal microscopy (see following section). Overall, for the characterization of the type of membrane anchor (Supplementary Figs. 8–10), an average *n* ≈ 15 vesicles was analyzed per each sample (*n*_total_ = 277 GUVs). For the characterization of the number, position and linker length required for cholesteryl-functionalized DNA origami structures (Fig. 2a, c, d and Supplementary Figs. 11–14), an average *n* ≈ 26 vesicles was analyzed per each sample (*n*_total_ = 1023 GUVs). For the membrane deformation assays triggered upon hyperosmotic stress (Fig. 3 and Supplementary Figs 15–21), an average *n* ≈ 42 vesicles was analyzed per each sample concentration (*n*_total_ = 2352 GUVs). For the determination of the binding coefficients (Fig. 4a, b and Supplementary Figs. 23, 24), an average *n* ≈ 13 vesicles was analyzed per each sample concentration (*n*_total_ = 975 GUVs). Finally, for the determination of the tubulation efficiencies after overnight incubation (Fig. 4c, d and Supplementary Fig. 25), an average *n* ≈ 22 vesicles was analyzed per each sample concentration (*n*_total_ = 860 GUVs).

### Laser scanning confocal fluorescence microscopy

Confocal imaging was performed on a commercial laser scanning microscope LSM 780 with a ConfoCor3 unit (Zeiss, Jena, Germany) using a water immersion objective (C-Apochromat, 40 × /1.2 W UV–VIS–IR, Zeiss, Jena, Germany). Samples were excited with the 488 nm line of an Ar-ion-laser (for Atto488 and Alexa488 excitation) or with the 633 nm line of a He–Ne laser (for Atto655 and DiD excitation). To avoid the effect of polarization selection in excitation of the GUVs, an achromatic *λ*/4 plate (Edmund Optics, Barrington, NJ, USA) was installed in the excitation beam path. Images were typically recorded at the equatorial planes of GUVs, utilizing a 1 Airy unit pinhole, 512 × 512 pixel resolution and a scan rate of 3.15 μs per pixel. Further image analysis was performed using the ImageJ software (http:// rsb.info.nih.gov/ij/).

As fluorescence signal measured using confocal microscopy is proportional to the number of fluorescent molecules in the confocal volume, fluorescence intensity of membrane-bound DNA origami was determined in order to infer membrane affinities of different nanostructures and assess particle densities on membranes (see FCS section). For this purpose, GUVs incubated overnight (4 °C) with different bulk concentrations of DNA origami, ranging from 0.01 to 50 nM, were imaged at the equatorial plane and the corresponding fluorescence intensities extracted from the confocal images using a semi-automated Matlab-based software^44^. As illustrated in Fig. 4a and Supplementary Fig. 23, apparent membrane dissociation constants at equilibrium (*K*_d_; Fig. 4b and Supplementary Fig. 24) for the different DNA origami nanostructures were then determined by fitting the fluorescence intensities of membrane-bound origami (*I*) as a function of total DNA origami concentrations in bulk (*C*_bulk_) to a Langmuir isotherm^9^:

1 $$I = I_{{\mathrm{max}}}/\left( {1 + K_{\mathrm{d}}/C_{{\mathrm{bulk}}}} \right),$$

### Fluorescence correlation spectroscopy

Fluorescence correlation spectroscopy (FCS) measurements were carried out as described in our recent publication^33^, using the LSM 780/ConfoCor 3 system mentioned above. Briefly, the laser line with wavelength of 488 nm for Atto488 excitation was used at low laser power (<1.2 μW) to avoid photobleaching and fluorescence saturation effect^57^. The radius of the waist of the FCS detection volume, *r*_0_ (207 ± 7 nm), was calibrated using a fluorescent dye (Alexa488) with known diffusion coefficient (*D*) in water (*D* (Alexa488) = 414 μm^2^ s^−1^ at 25.0 ± 0.5 °C)^58^ and corrected for the working temperature at the objective (27.5 ± 1.0 °C)^57,59,60^. FCS on membranes was performed at the upper pole of a GUV with a diameter of at least 20 μm (which is large enough to neglect membrane curvature within the detection spot size). Particle numbers, *N*, (and consequently, surface densities, *σ*) of the BAR-mimicking DNA nanostructures were obtained from the analysis of the autocorrelation functions, using the freely available data analysis software PyCorrFit version 0.8.2^61^. In order to eliminate the contribution of rotational diffusion to the correlation curves, DNA origami structures labeled at positions T2-4 were used^33^. Furthermore, as virtually no unbound DNA origami was detected in solution, and its potential contribution to FCS curves was negligible, a one-component two-dimensional diffusion model^56, 57^ was used (equation 2) to analyze the obtained correlation curves, as it was done in previous studies of membrane-bound DNA origami particles^33, 62, 63^.

2 $${\it{G}}({\it{\tau }}) = \frac{1}{N}\frac{1}{{1 + \frac{\tau }{{\tau _{\mathrm{D}}}}}},$$

Here *N* is the number of particles in the 2D detection volume, and *τ*_D_ is the FCS diffusion time, which is determined by the translational diffusion coefficient *D* and the size of the 2D Gaussian detection volume *r*_0_ as follows: $\tau _D = r_0^2/(4D)$.

Knowing the origami length, *L* = 110 nm, surface densities of membrane-bound particles *σ* ($\sigma = N/({\mathrm{\pi }}r_0^2)$; expressed in particles per µm^2^) could be easily converted to the reduced surface densities *ρ = σL*^2^ ^62^. At higher surface densities (*ρ* > 0.2), crowding effects resulted in progressively stronger deviations from the one-component 2D diffusion model used to describe the translational Brownian motion of the Atto488-labeled DNA origami particles^62^. As particle density is proportional to the fluorescence intensity, average surface densities of membrane-bound DNA origami could be estimated at a high-density regime (*ρ* > 0.2) from the fluorescence intensity data obtained via confocal microscopy. Shortly, a calibration curve was obtained (Supplementary Fig. 22b) from the linear fit of the fluorescence intensity of membrane-bound DNA origami determined by confocal microscopy for single GUVs (*n* = 45) and the respective surface densities of membrane-bound DNA origami determined by FCS in the valid density regime (*ρ* < 0.2—Supplementary Fig. 22a).

### Atomic force and transmission electron microscopies

Atomic force microscopy (AFM) imaging of structures L0, Q0, and H0, deposited on top of freshly cleaved mica, was performed on a Nanowizard Ultra (JPK, Berlin, Germany) using the high-speed AC mode with USC-F0.3-k0.3 cantilevers (Nanoworld, Neuchâtel, Switzerland)^33^. The cantilever oscillation was turned to a frequency of 100–150 kHz, the amplitude kept below 10 nm. Scan rate was set to 5–25 Hz and setpoints close to 7-8 nm were utilized. Analysis of the AFM images was performed using JPK SPM Data Processing (version 5.1.4) and Gwyddion (version 2.30).

Negative-stain transmission electron microcopy (TEM) imaging was performed on a Philips CM100 transmission electron microscope operated at 100 kV^17, 54^. Images were recorded with an AMT 4 × 4 Megapixel CCD camera. Typically, 3 µL of folded DNA origami nanostructures were adsorbed on glow-discharged formvar-supported carbon coated Cu400 TEM grids (Science Services, Munich, Germany) and stained using a 2% aqueous uranyl formate solution containing 25 mM sodium hydroxide. For the experiments involving multimellar vesicles (MLV), 4 nM of origami Q0 or Q3 were pre-incubated for 30 min with DOPC MLV (at 0.5 mM lipid concentration) before deposition on the EM grids and negative staining.

For cryo-electron microscopy (cryo-EM), 5 nM Q3 was pre-incubated overnight in a tube with DOPC GUVs. Samples were then adsorbed for 4 min on glow-discharged lacey carbon grids (Plano, Wetzlar, Germany) and vitrified by plunge freezing the grid in liquid ethane. Imaging was performed on a Titan Halo electron microscope (FEI, Eindhoven, Netherlands), equipped with a Falcon II camera and a Gatan 626 cryo holder (Pleasanton, CA, USA). The microscope was operated at 300 kV, with a magnification of ×45,000, giving a pixel size of 0.237 nm at the specimen level. Data were collected using SerialEM, at nominal −3 µm target defocus with an electron dose of 20 e^−^ Å^−2^. Tubular diameter (average ± s.d.) was obtained analyzing *n*_total_ = 35 parallel cross-sections along four Q3-decorated membrane tubules.

### Estimation of the energetic costs for membrane bending

The energy required for membrane bending by curved DNA origami scaffolds Q and H and a BAR domain protein were calculated using the Area-difference Elasticity (ADE) model^39, 40^. This model, based on the classical Helfrich-Canham-Evans elastic membrane model (spontaneous curvature model)^64^, takes into consideration the finite thickness of the lipid bilayer and consequent additional penalty arising from the area difference between its two leaflets upon bending (i.e., negatively curved leaflet being compressed, while positively curved leaflet being expanded). The ADE model describes bending energy ($\varepsilon _{{\mathrm{be}}}$) as:

3 $$\varepsilon _{{\mathrm{be}}} = \kappa \left( {\frac{1}{2}{\int} {{\mathrm{d}}A\left( {C_1 + C_2 - C_0} \right)^2} + \frac{\alpha }{2}\frac{{\mathrm{\pi }}}{{AD^2}}\left( {{\mathrm{\Delta }}A - {\mathrm{\Delta }}A_0} \right)^2} \right),$$

where $\kappa$ is the bending modulus of DOPC bilayers (23.1 *k*_*B*_*T*)^65^, *A* is the area of the membrane segment, *C*_1_ and *C*_2_ are the principal curvatures (for a membrane tube, $C_1 = 1/R$ and $C_2 = 0$). *C*_0_ is spontaneous curvature of the membrane, which relates to the intrinsic curvature of the lipid molecules. For a homogenous non-asymmetric bilayer, *C*_0_ = 0. In the second term, ${\mathrm{\Delta }}A$ is the differential monolayer area (determined by the difference in number of molecules of the outer and the inner monolayers) and ${\mathrm{\Delta }}A_0$ its value at equilibrium. *D* corresponds to the membrane thickness. $\alpha = \bar \kappa /\kappa$, with $\bar \kappa$ being the non-local bending rigidity modulus. *α* is estimated to be in the order of unity and the approximation $\alpha = 3/{\mathrm{\pi }}$^66^ was used.

### Data availability

Data supporting the findings of this manuscript are available from the corresponding author upon reasonable request.

## Electronic supplementary material

Supplementary Information

Peer Review File

Description of Additional Supplementary Files

Supplementary Movie 1

Supplementary Movie 2

Supplementary Movie 3

Supplementary Movie 4
