# Supplementary material for: Membrane sculpting by curved DNA origami scaffolds
Source: Nat Commun. 2018 Feb 23;9:811. doi: 10.1038/s41467-018-03198-9 (PMC5824810; doi:10.1038/s41467-018-03198-9)
Supplement: Supplementary file 1 — Supplementary Information [file 41467_2018_3198_MOESM1_ESM.pdf]

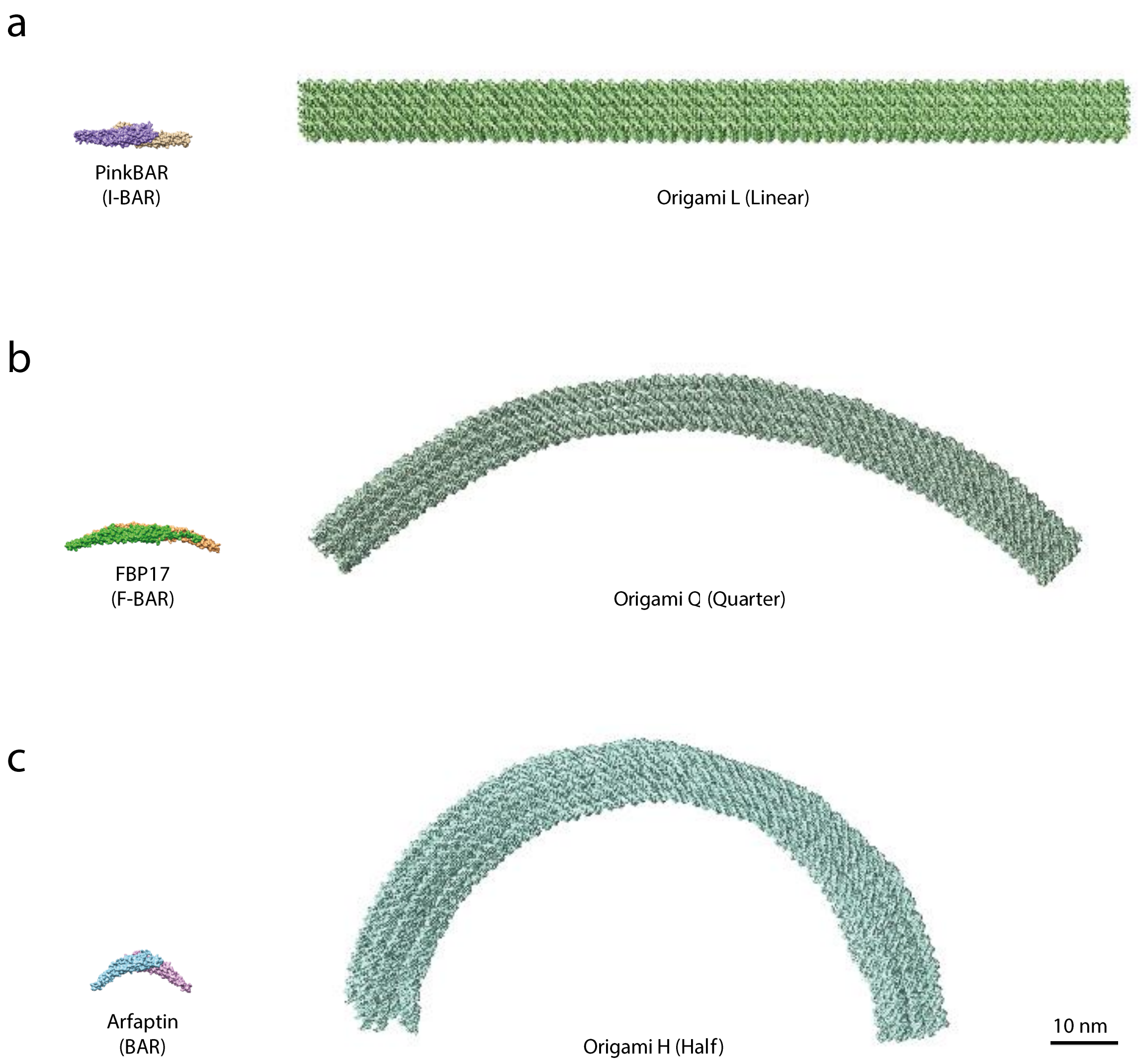

Supplementary Figure 1

Size comparison between BAR proteins and the designed BAR-mimicking DNA origami nanoscaffolds. (**a**) DNA origami L aims to mimic the shape of flat I-BAR domain proteins, such as PinkBAR; (**b**) origami Q aims to mimic the shape of moderately-curved F-BAR domain proteins, such as FBP17; and (**c**) origami H aims to mimic the shape of highly-curved BAR domain proteins, such as Arfaptin. When compared to BAR dimers, our DNA origami structures have at least a 5-fold increased length and 20-fold increased concave surface area. Scale bar: 10 nm.

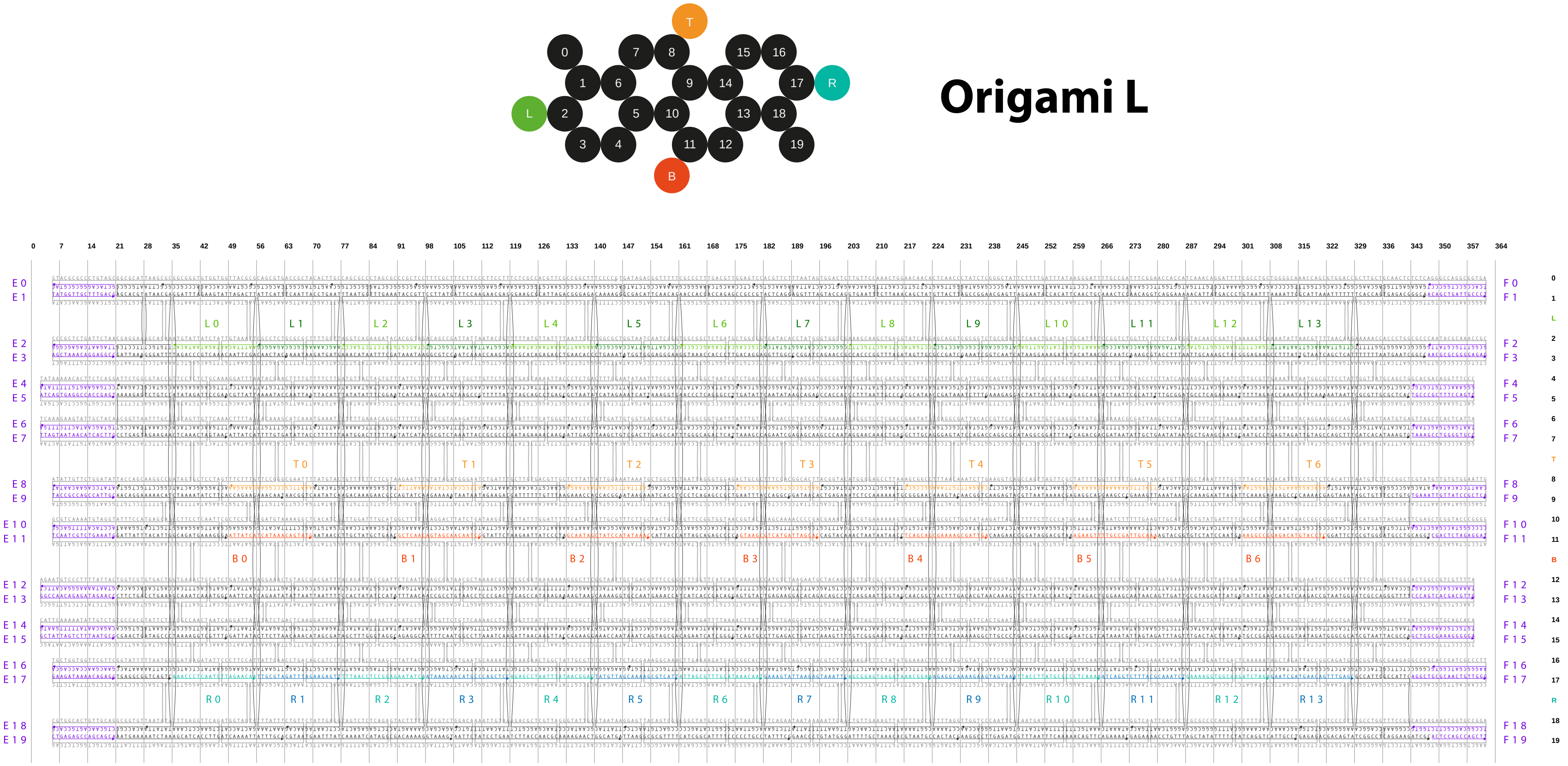

Supplementary Figure 2

Cadnano design and oligonucleotide sequences of the non-curved DNA origami structure L. Top positions T0-T7 are colored in gold, while bottom positions B0-B7 are colored in dark orange. Lateral positions L0-L13 and R0-R13 are colored in green and blue, respectively. Edge positions E0-E19 and F0-F19 are colored in purple. Core staples are colored in black; M13 p7249 scaffold is colored in grey. List of DNA staples can be found in Supplementary Table 1. Additional list of functional staples utilized can be found in appendix (see Supplementary Notes).

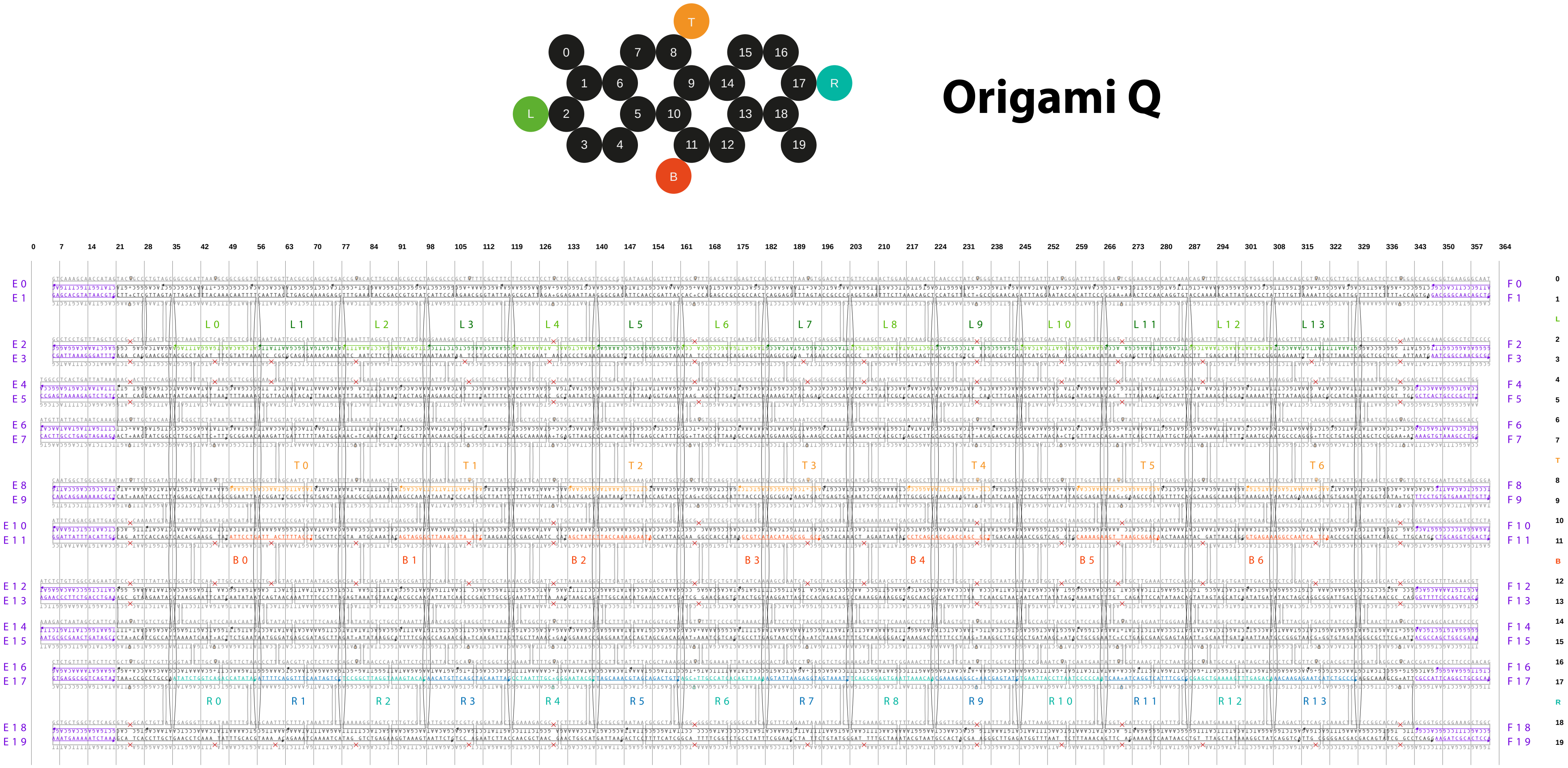

Supplementary Figure 3

Cadnano design and oligonucleotide sequences of the moderately-curved DNA origami structure Q. Top positions T0-T7 are colored in gold, while bottom positions B0-B7 are colored in dark orange. Lateral positions L0-L13 and R0-R13 are colored in green and blue, respectively. Edge positions E0-E19 and F0-F19 are colored in purple. Core staples are colored in black; M13 p7249 scaffold is colored in grey. List of DNA staples can be found in Supplementary Table 2. Additional list of functional staples utilized can be found in appendix (see Supplementary Notes).

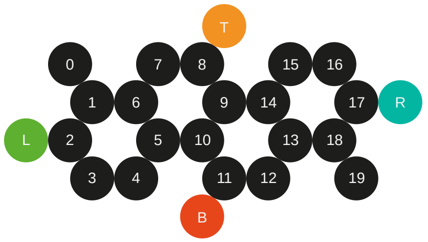

Origami H

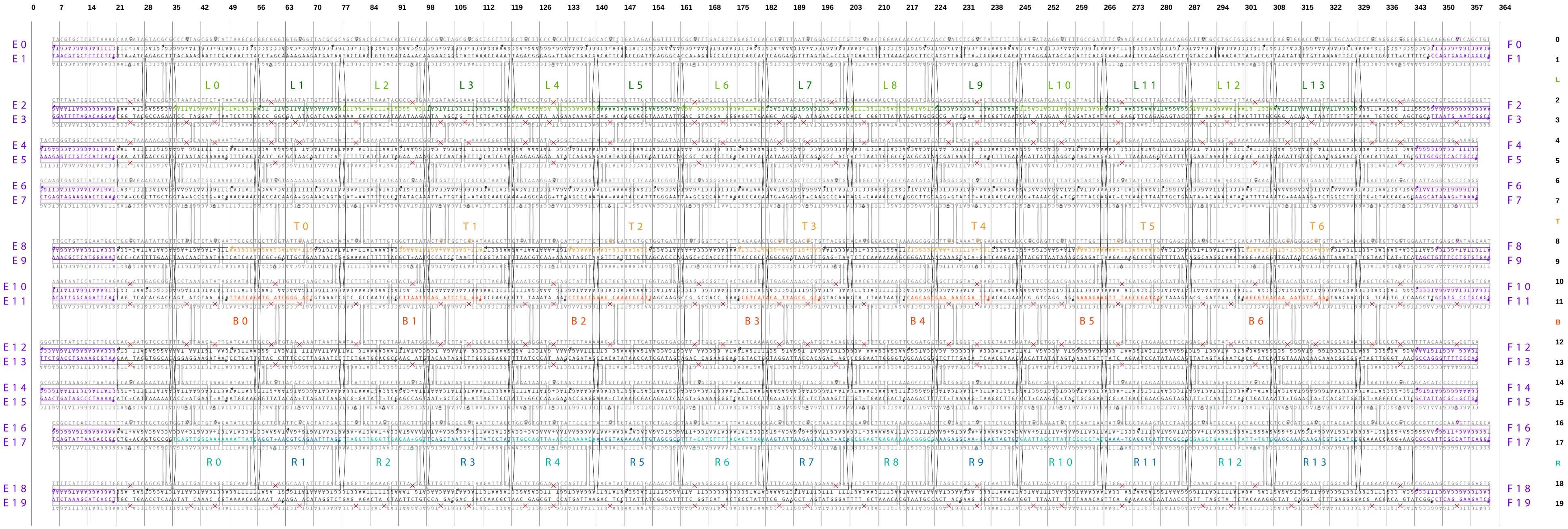

Supplementary Figure 4

Cadnano design and oligonucleotide sequences of the highly-curved DNA origami structure H. Top positions T0-T7 are colored in gold, while bottom positions B0-B7 are colored in dark orange. Lateral positions L0-L13 and R0-R13 are colored in green and blue, respectively. Edge positions E0-E19 and F0-F19 are colored in purple. Core staples are colored in black; M13 p7249 scaffold is colored in grey. List of DNA staples can be found in Supplementary Table 3. Additional list of functional staples utilized can be found in appendix (see Supplementary Notes).

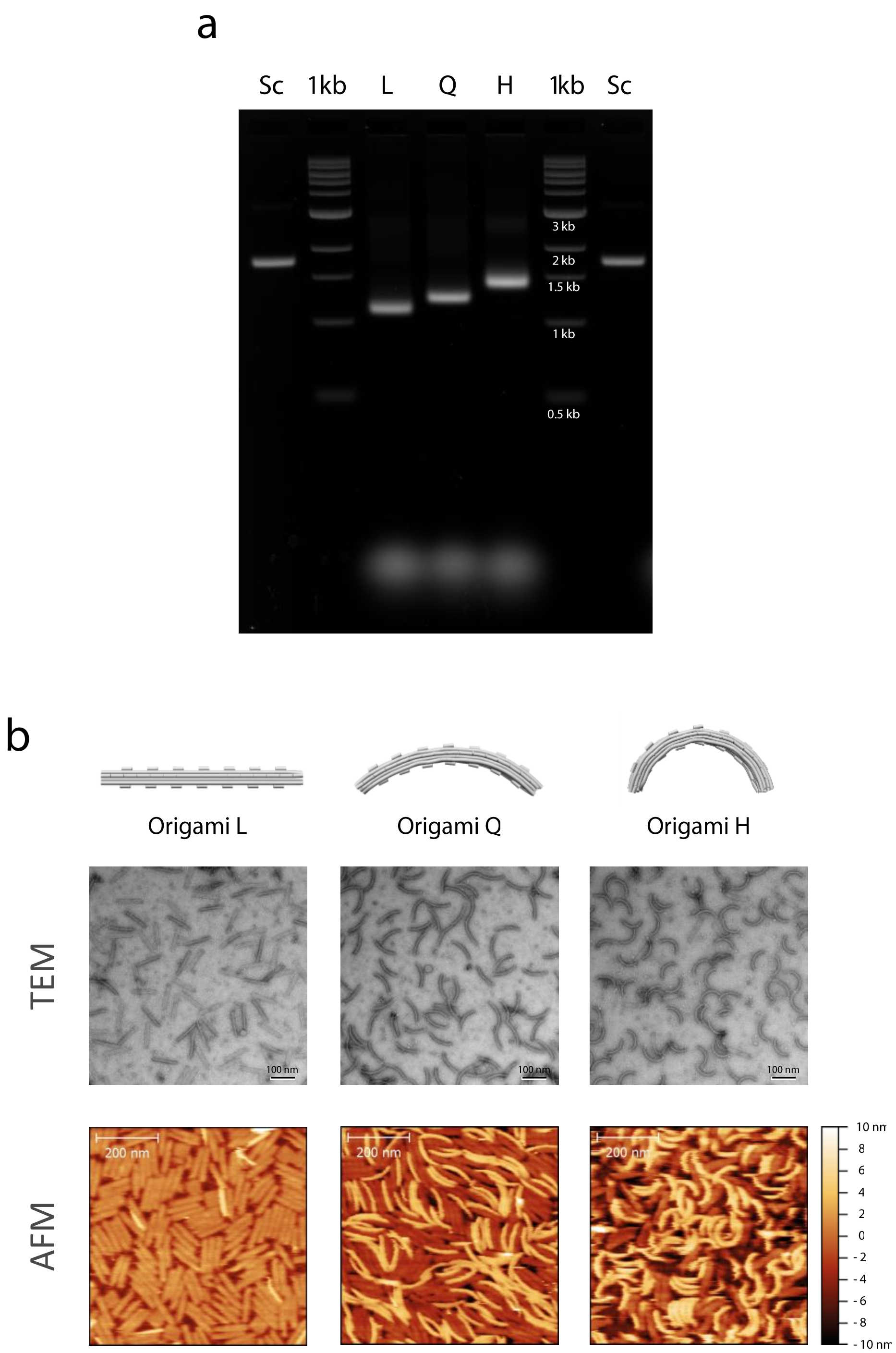

Supplementary Figure 5

Characterization of folded DNA origami nanoscaffolds. (**a**) Assembly of the folded bare origami structures L, Q, H was initially assessed via agarose gel (2%) electrophoresis analysis. Lanes containing marker DNA ladder (1kb) and M13 single-stranded p7249 scaffold (Sc) were also included. (**b**) Structure of folded bare origami L, Q and H was further validated using negative-stain transmission electron microscopy (TEM; scale bars: 100 nm) and atomic force microscopy (AFM; scale bars: 200nm).

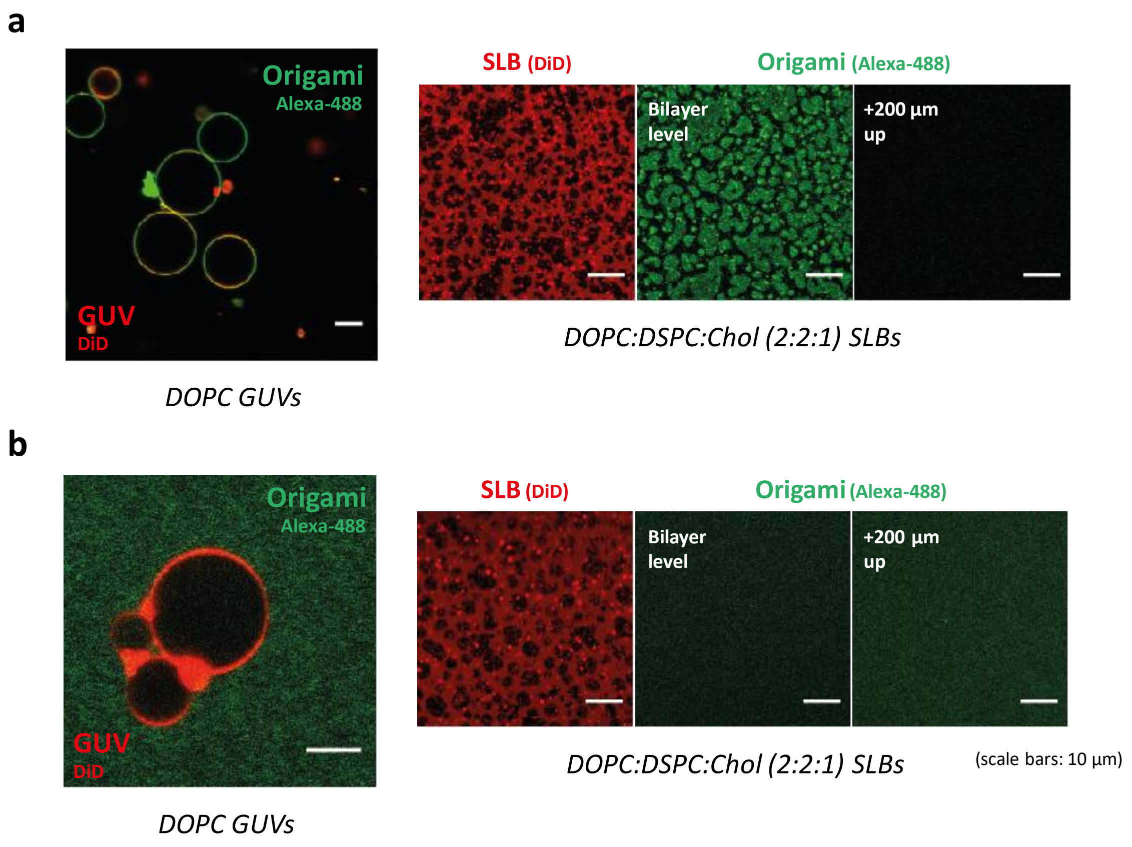

Supplementary Figure 6

Unspecific interaction of curved DNA origami with model lipid membranes, mediated by Mg^{2+}. Binding of bare origami H (labeled with Alexa488, green) to the surface of DOPC GUVs and DOPC:DSPC:Chol (2:2:1 mol ratio) supported lipid bilayers (SLBs) in a 5 mM Tris-HCl, 1 mM EDTA, pH 8.0 buffer containing (**a**) 20mM $MgCl_2$ and no NaCl or (**b**) a buffer with no $MgCl_2$ and 150mM NaCl. DOPC GUVs were electroformed in sucrose solutions iso-osmolar to the buffers utilized ((**a**) 70 mOsm kg^{-1} and (**b**) 300 mOsm kg^{-1}). For fluorescence detection, membranes were labeled with 0.1 % DiD (far-red fluorescent dye). As seen in panel (**a**), presence of a buffer containing 20 mM $MgCl_2$ and no NaCl led to extensive binding of DNA origami (labeled with green fluorescent dye Alexa488) to DOPC GUVs and to the liquid-ordered phase of DOPC:DSPC:Chol SLBs (dark regions). On the other hand as seen in panel (**b**), in the presence of a buffer containing no $MgCl_2$ and 150 mM NaCl, binding of DNA origami to the surface of membranes was not observed, as green fluorescence can be only found in solution and not at the membrane level. Scale bars:10 µm.

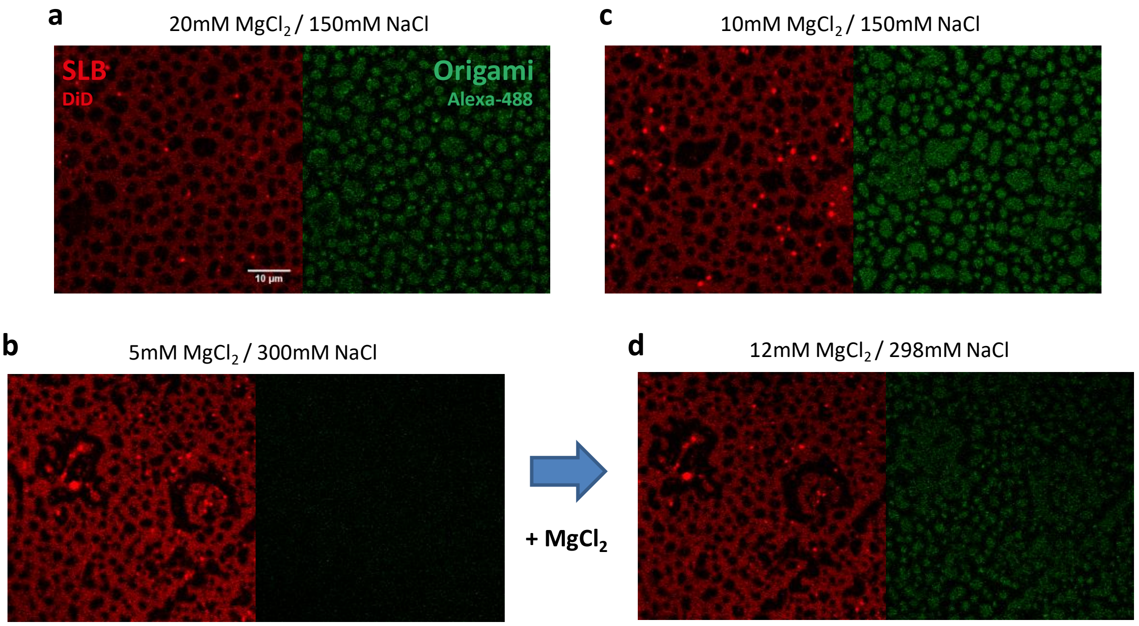

Supplementary Figure 7

Distinct amounts of $MgCl_2$ and NaCl in solution modulate the attachment of bare DNA origami to membranes. Varying amounts of $MgCl_2$ and NaCl in solution allowed (**a-b**) or prevented (**c**) unspecific attachment of bare DNA origami H (labeled with Alexa488, green) to lipid bilayers (DOPC:DSPC:Chol (2:2:1) SLBs; labeled with DiD, red). Upon addition of $MgCl_2$ to the lipid bilayer displayed in panel (**c**), binding of bare origami H to the membrane could be triggered (**d**). Scale bar: 10 µm.

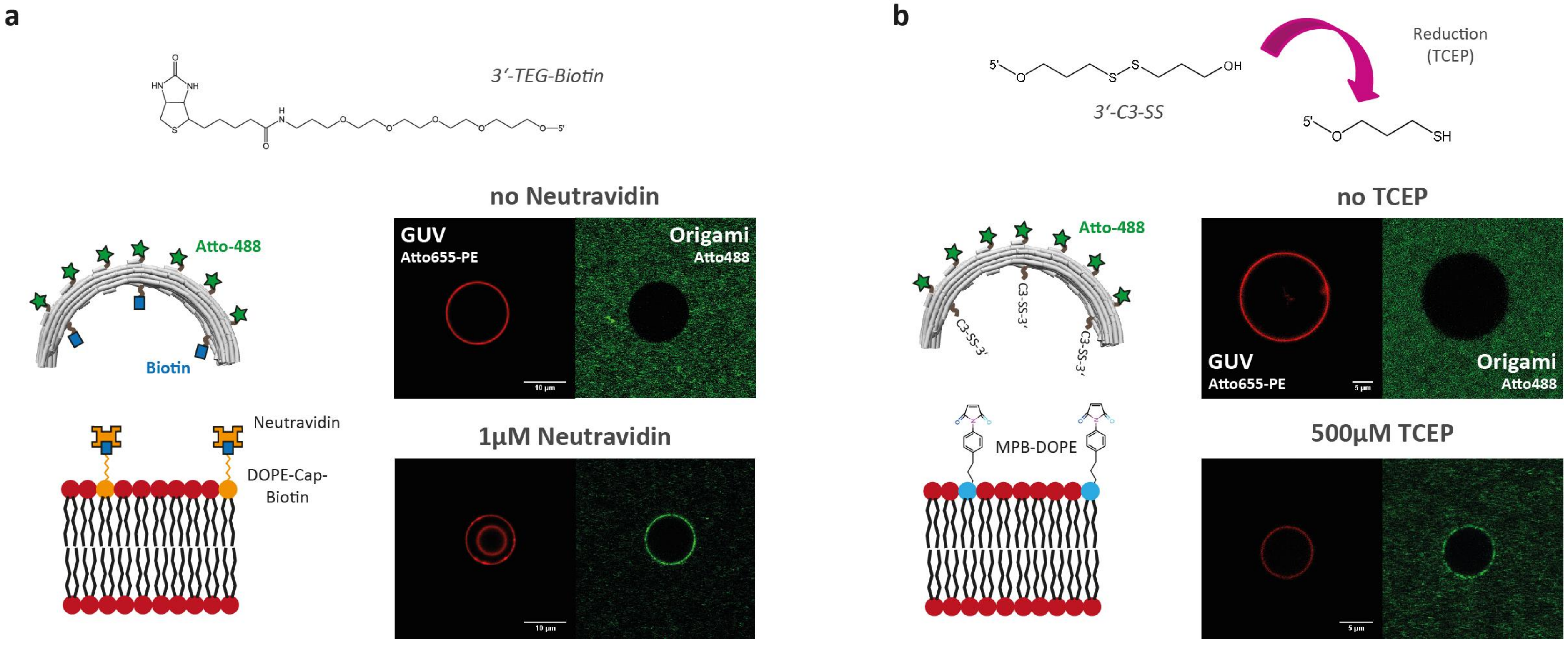

Supplementary Figure 8

Specific attachment of curved origami H to lipid membranes via (a) high affinity polyvalent neutravidin-biotin interaction and (b) covalent attachment. (a) DOPC GUVs containing 2 mol% of biotin-modified DOPE molecules (DOPE-Cap-Biotin) were incubated with DNA nanostructures H containing 3'-TEG-biotin functionalized oligonucleotide staples at origami positions B0, B3 and B6. Upon addition of neutravidin (1 µM) and after less than 1h incubation, extensive binding of the curved nanostructure to the surface of GUVs was observed. **(b)** DOPC GUVs containing 10 mol% of maleimide-modified DOPE lipid molecules (MPB-DOPE) were incubated with DNA origami nanostructure H containing protected 3'-thiol-modified strands (3'-C3-SS at positions B0, B3 and B6). Upon addition of excess concentration of reducing agent (TCEP), which would cleave the protecting disulfide bonds, and after overnight incubation, the thiolated DNA origami nanostructure H was able to be crosslinked to GUVs, although not as efficiently as with the biotin-neutravidin strategy. For these experiments, GUVs were fluorescently-labeled with 0.05 mol% Atto655-DOPE (red) and the DNA origami nanostructures with Atto488-modified strands (green). Moreover, while for strategy **(a)** standard imaging buffer (5 mM Tris-HCl, 1 mM EDTA, 5 mM $MgCl_2$, 300 mM NaCl, pH 8.0) was utilized, in case of strategy **(b)** we utilized a 10 mM Hepes, 5 mM $MgCl_2$, 300 mM NaCl, pH 7.4 buffer, as the thiol-maleimide crosslinking requires a lower pH and a non-amine buffer. Scale bars: **(a)** 10 µm; **(b)** 5 µm.

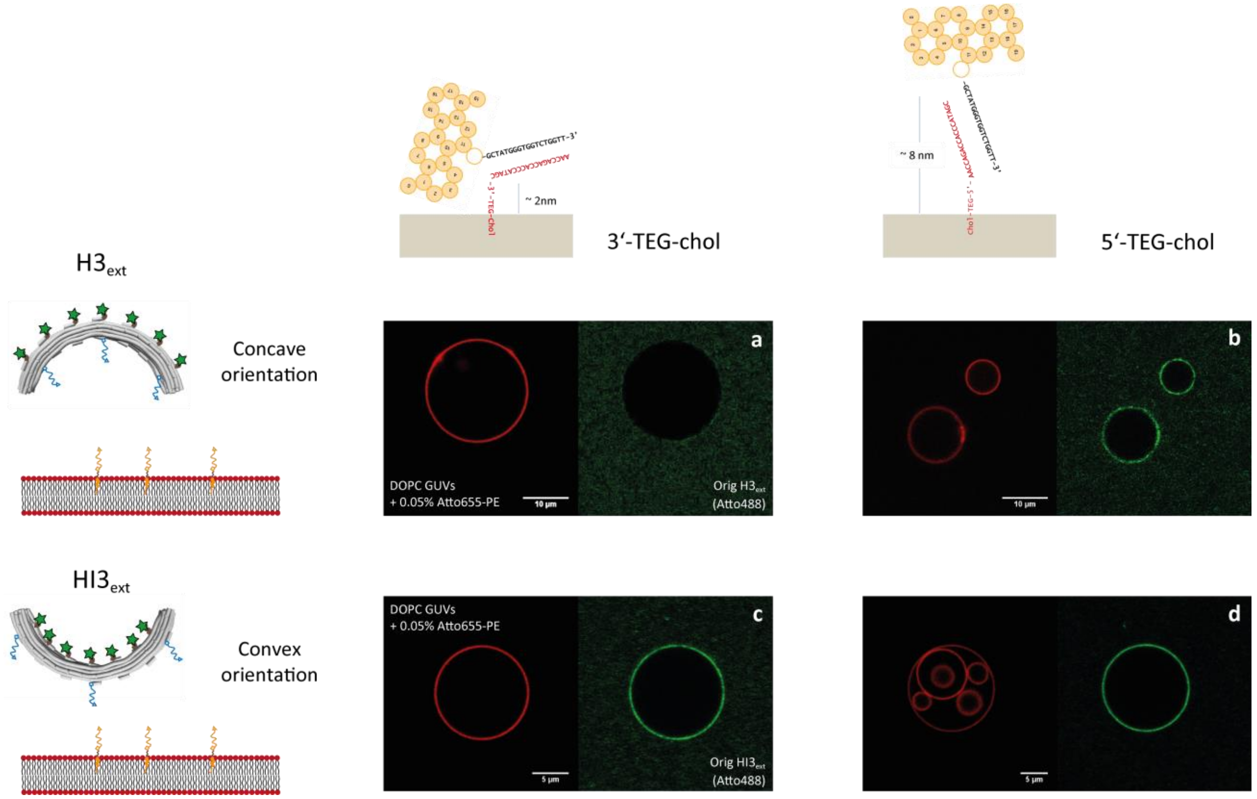

Supplementary Figure 9

Side-specific attachment of origami H to GUVs through hybridization with TEG-chol modified oligonucleotides. Origami H nanostructures (labeled with Atto488, green) presenting 18 nucleotide-long single-stranded overhangs (**a-b**) on the concave surface – origami $H3_{ext}$ (overhangs extending form the 3'-end of bottom positions B0, B3 and B6) or (**c-d**) on the convex surface – origami $HI3_{ext}$ (overhangs extending form the 3'-end of top positions T0, T3 and T6), were added to DOPC GUVs (labeled with Atto655-DOPE, red) previously pre-incubated with 2 µM of 3'/5'-modified TEG-chol modified complementary oligonucleotide strands. **Panels (a,c):** when 3'-end TEG-chol functionalized oligonucleotides were used (proximal orientation), hybridization between the DNA overhangs on origami H and membrane-bound complementary strands will occur in tight proximity to lipid bilayer surface. Due to the bulkiness and curvature of this nanostructure, membrane attachment of structure $H3_{ext}$ via its concave surface, will be hindered (**a**). On the other hand, significant binding of structure $HI3_{ext}$ to membranes via its more accessible convex surface was still observed (**c**). **Panels (b,d):** when 5'-end TEG-chol functionalized oligonucleotides are used (distal orientation), hybridization between the DNA overhangs on origami H and membrane-bound complementary strands will be more accessible. Independently of the concave ($H3_{ext}$; (**b**)) or convex ($HI3_{ext}$; (**d**)) binding orientation, strong attachment of the curved origami H could be achieved. Scale bars: **(a, b)** 10 µm; **(c, d)** 5 µm.

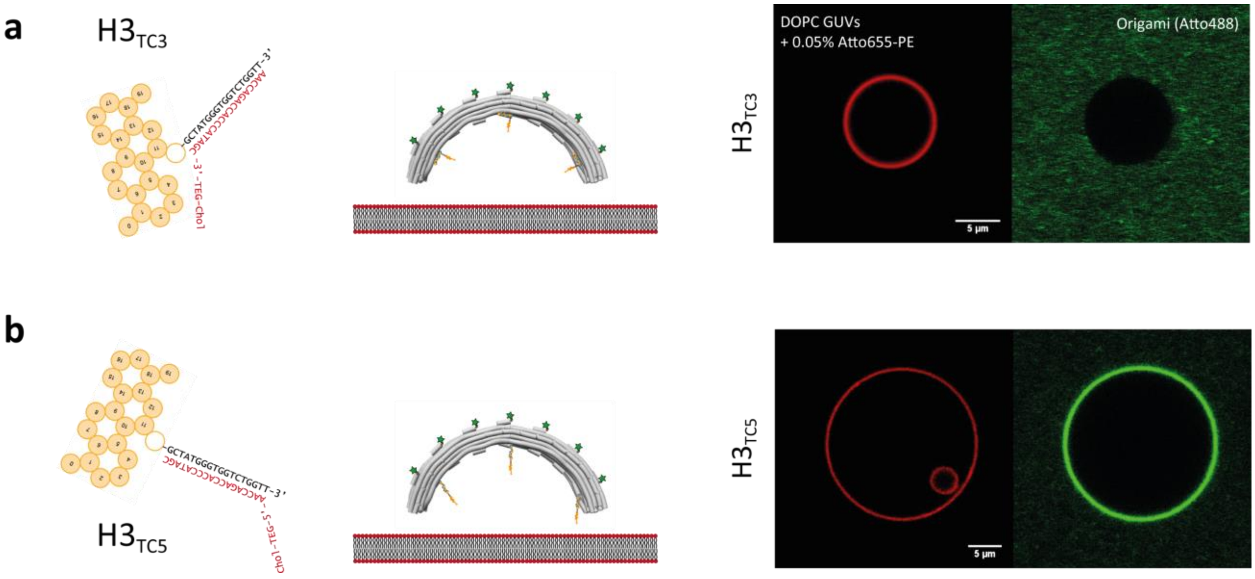

Supplementary Figure 10

Attachment of origami H to GUVs through TEG-chol modified 18bps overhangs. Nanostructures H (labeled with Atto488, green) displaying 3'/5'-TEG-chol modified 18 nucleotide-long DNA strands, now directly hybridized during origami folding with single-stranded overhangs at the bottom positions B0, B3 and B6, were used. **(a)** Proximal positioning of the three TEG-chol modifications on the concave surface (H3$_{TC3}$; TEG-Chol at 3'-end) resulted in virtually no attachment of origami H to DOPC GUVs (labeled with Atto655-DOPE, red), even after longer incubation times (i.e. overnight). **(b)** On the other hand, by placing the three TEG-Chol moieties at the distal end of the 18 bps overhangs (H3$_{TC5}$; TEG-Chol at 5'-end) strong membrane binding of origami H after 1h of incubation was observed. Hence, the increase of the linker length from ~2 nm (length of the TEG moiety) to ~8 nm (combined length of the TEG moiety and the 18 bps overhang) rendered the cholesteryl moieties on the curved and bulky origami H nanostructures less sterically hindered and more accessible for binding macroscopically flat lipid membranes, such as GUVs. Scale bars: 5 µm.

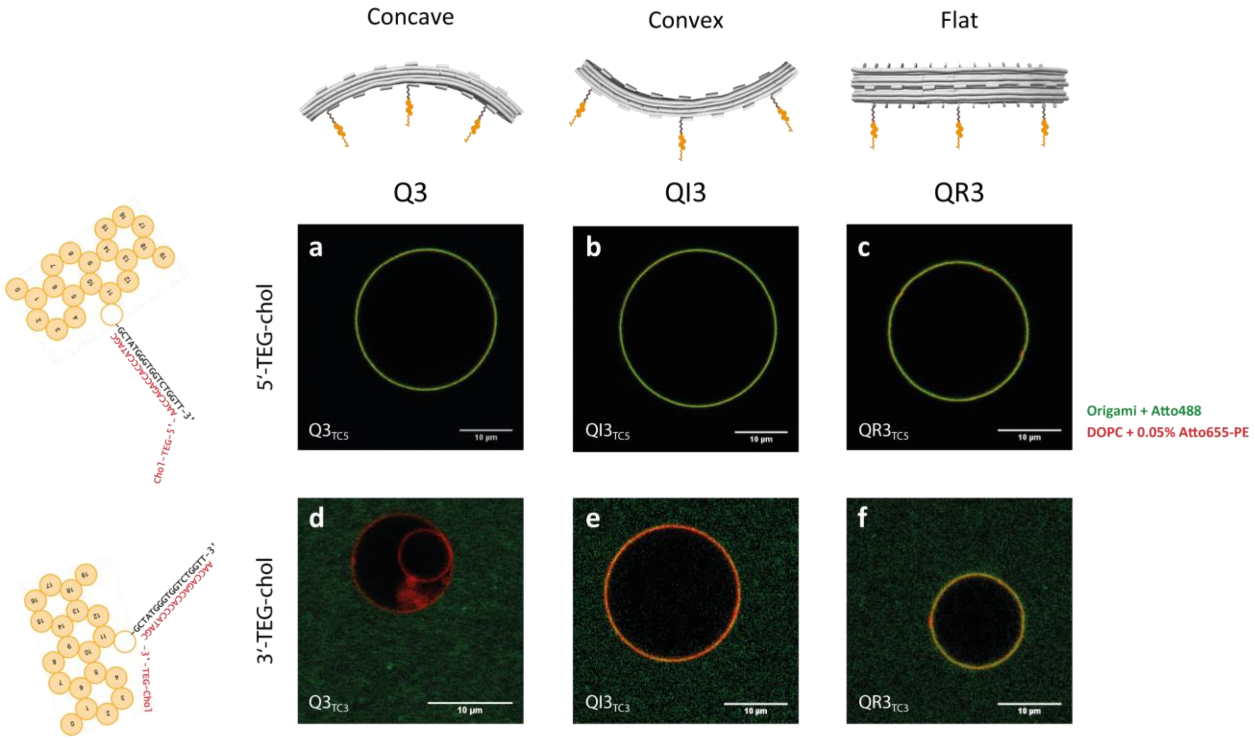

Supplementary Figure 11

Side-specific attachment of origami Q to GUVs through TEG-chol modified 18bps overhangs. We further assessed binding to DOPC GUVs (labeled with Atto655-DOPE, red) of origami Q nanostructures (labeled with Atto488, green), previously functionalized with three TEG-chol moieties at the distal 5' (TC5) and proximal 3' (TC3) ends of 18 bps overhangs at the concave (bottom positions B0, B3 and B6; origami $Q3_{TC\underline{X}}$), convex (top positions T0, T3 and T6; origami $QI3_{TC\underline{X}}$) or lateral non-curved (right positions R0, R6 and R12; origami $QR3_{TC\underline{X}}$) origami surfaces. **(a-c)**: Independently of the surface of the nanostructures carrying the modifications, distal positioning of the TEG-chol moieties (TC5) resulted in strong binding of the DNA origami Q nanostructures to GUVs. **(d-f)**: When the TEG-chol moieties were positioned in close proximity to the DNA origami core (TC3), membrane binding of those nanostructures depended on which surface was carrying hydrophobic modifications. If the cholesteryl groups are localized on the concave surface of origami Q (origami $Q3_{TC3}$, (**d**)), no significant binding to GUVs was reported. On the other hand, if the cholesteryl groups were localized on the convex surface of origami Q ($QI3_{TC3}$, (**e**)) and mainly lateral surface with essentially zero curvature ($QR3_{TC3}$, (**f**)), significant (yet weaker) membrane binding was observed. Scale bars: 10 µm.

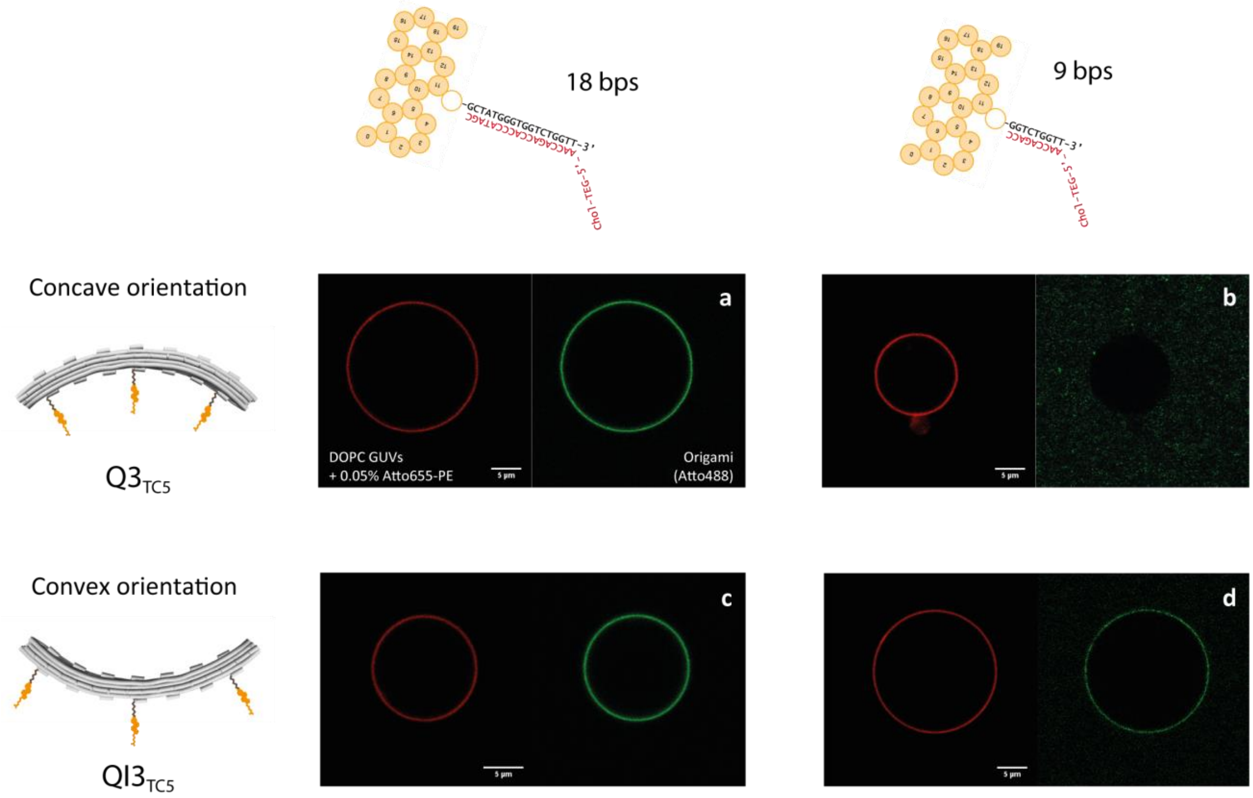

Supplementary Figure 12

Dependence on the length of the TEG-chol modified linkers/overhangs, for attaching origami Q to GUVs. Independently of the origami surface on which the distal 5'-TEG-chol moieties localized, for structure $Q3_{TC5}$ (cholesteryl anchors at the concave facet of origami Q) and $QI3_{TC5}$ (cholesteryl anchors at the convex facet of origami Q) with 18 bps overhangs (linker length ~8 nm), a strong attachment to lipid membranes was observed ((**a**) and (**c**), respectively). However, shortening those overhangs to 9 bps (linker length ~5 nm) resulted in a strong reduction of the binding of curved nanostructures Q to the GUVs. For such linker length, weak binding to lipid membranes was still observed for structure $QI3_{TC5}$ with hydrophobic anchors at the convex origami facet (**d**); yet no membrane binding of structure $Q3_{TC5}$ with anchors at the concave origami surface (**b**) was reported. Overall, placing the TEG-chol moieties at the distal end of 18 bps overhangs provided an appropriate linker length for overcoming steric hindrance during the attachment of our nanostructures to GUVs. Scale bars: 5 µm. Green fluorescence: origami labeled with Atto488; red fluorescence: membranes labeled with Atto655-DOPE.

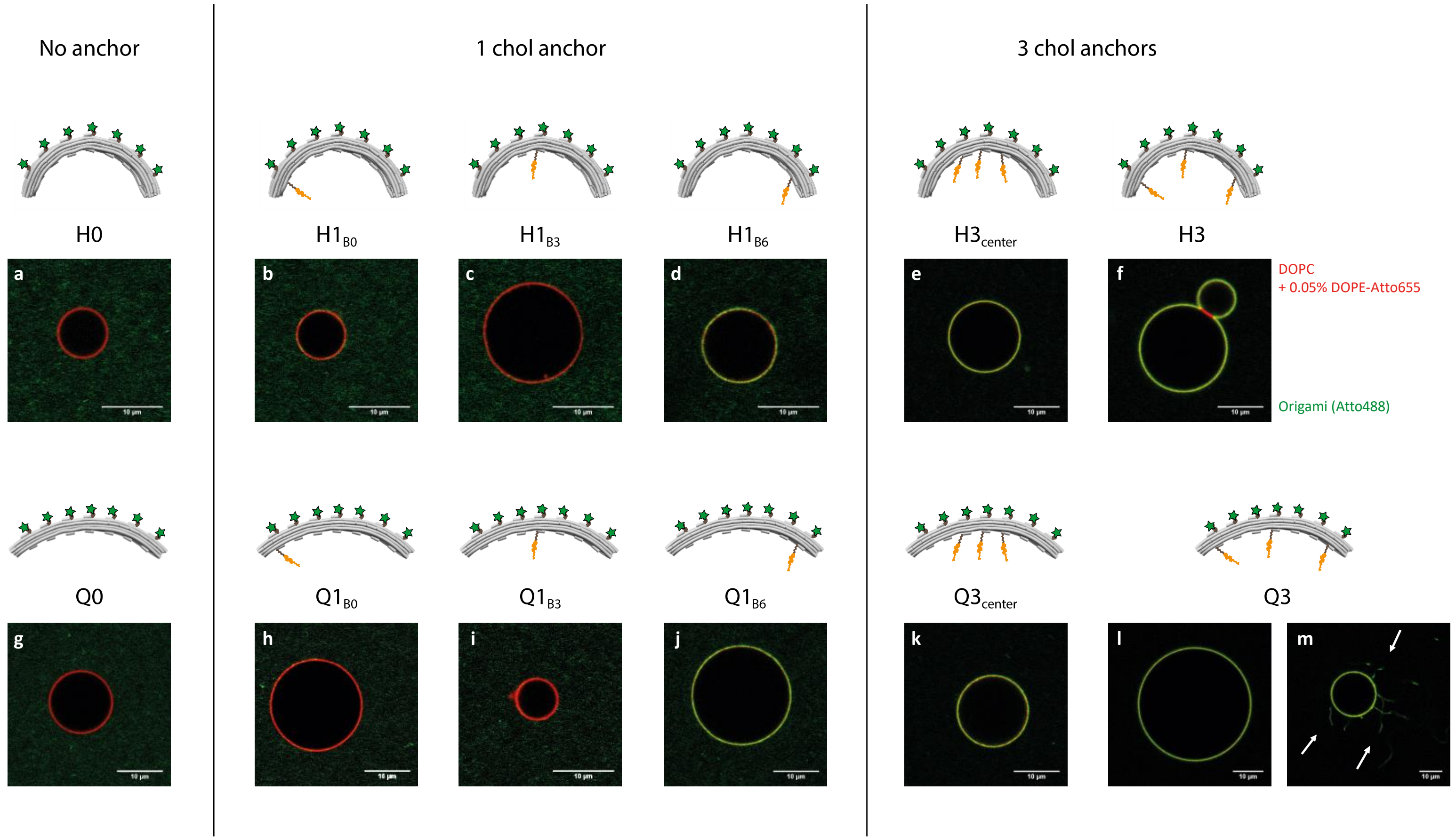

Supplementary Figure 13

Effect of the number and positioning of TC5 anchors on the binding of curved DNA origami nanostructures to freestanding lipid bilayers via their concave surface. TEG-chol moieties placed at the distal 5'-end of 18 bps overhangs (from here on called TC5) were used at the bottom concave origami positions. In the absence of TC5 anchors and using imaging buffer containing 5 mM $MgCl_2$ and 300 mM NaCl, bare origami structures H0 and Q0 did not attach to GUVs (**a**, **g**). Use of single TC5 anchors on structures H1 and Q1 did not promote efficient membrane binding of the nanostructures (**b**, **c**, **h**, **i**), unless the TC5 anchor was placed at the most accessible edge position B6 (**d**, **j**), indicating that steric hindrance clearly affected membrane binding. Strong binding to GUVs was however achieved upon incorporation of 3× TC5 anchors; independently of the anchors being localized at the central positions B2, B3 and B4 ($H3_{center}$, (**e**); $Q3_{center}$, (**k**)) or distributed across the concave facet at positions B0, B3 and B6 (H3, (**f**); Q3, (**l**)). Although membrane attachment did not greatly depend on the degree of curvature of our nanostructures, only in the presence of the moderately-curved structure Q3 a significant amount of GUVs (~ 18%) displayed tubular-like outwards deformations (arrows, (**m**)). Scale bars: 10 µm. Green fluorescence: origami labeled with Atto488; red fluorescence: membranes labeled with DOPE-Atto655.

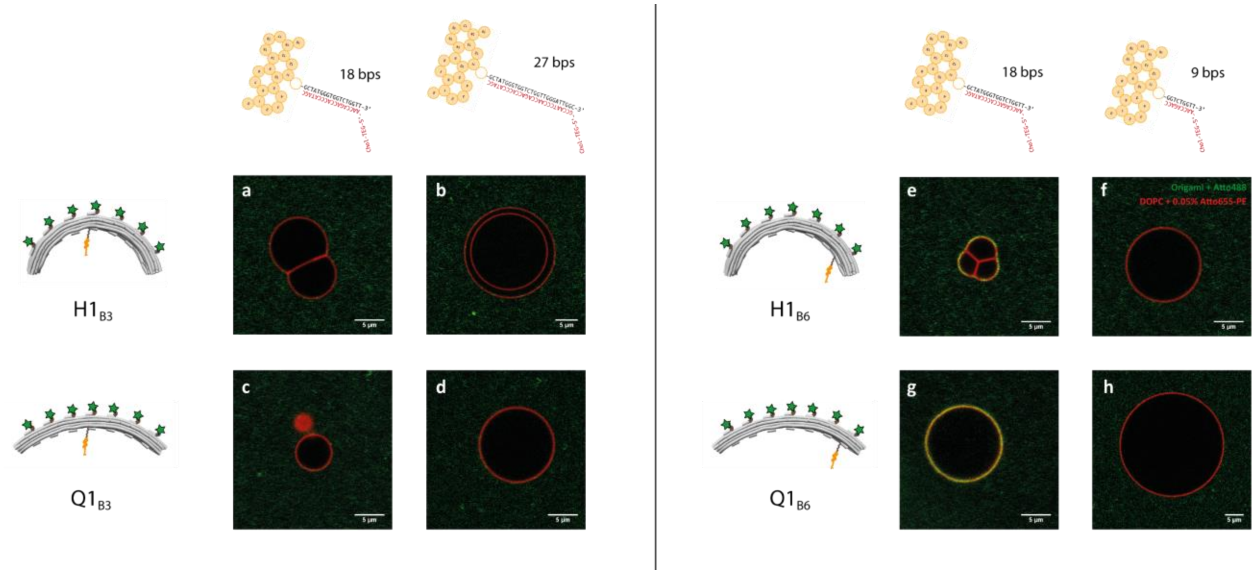

Supplementary Figure 14

Membrane binding of curved DNA nanostructures with single TC5 anchors is dependent on the linker/overhang length. The use of a longer linker/overhang (27 bps, instead of the typical 18 bps) did not improve membrane binding of nanostructures $H1_{B3}$ and $Q1_{B3}$ containing a single TC5 anchor at the central concave position B3 (**a-d**). On the other hand, shortening the linker/overhang from 18 to 9 bps for single TC5 anchors placed at the most accessible edge position B6, fully abolished the weak membrane binding observed for both DNA origami $H1_{B6}$ and $Q1_{B6}$ nanostructures (**e-h**). Scale bars: 5 µm. Green fluorescence: origami labeled with Atto488; red fluorescence: membranes labeled with Atto655-DOPE.

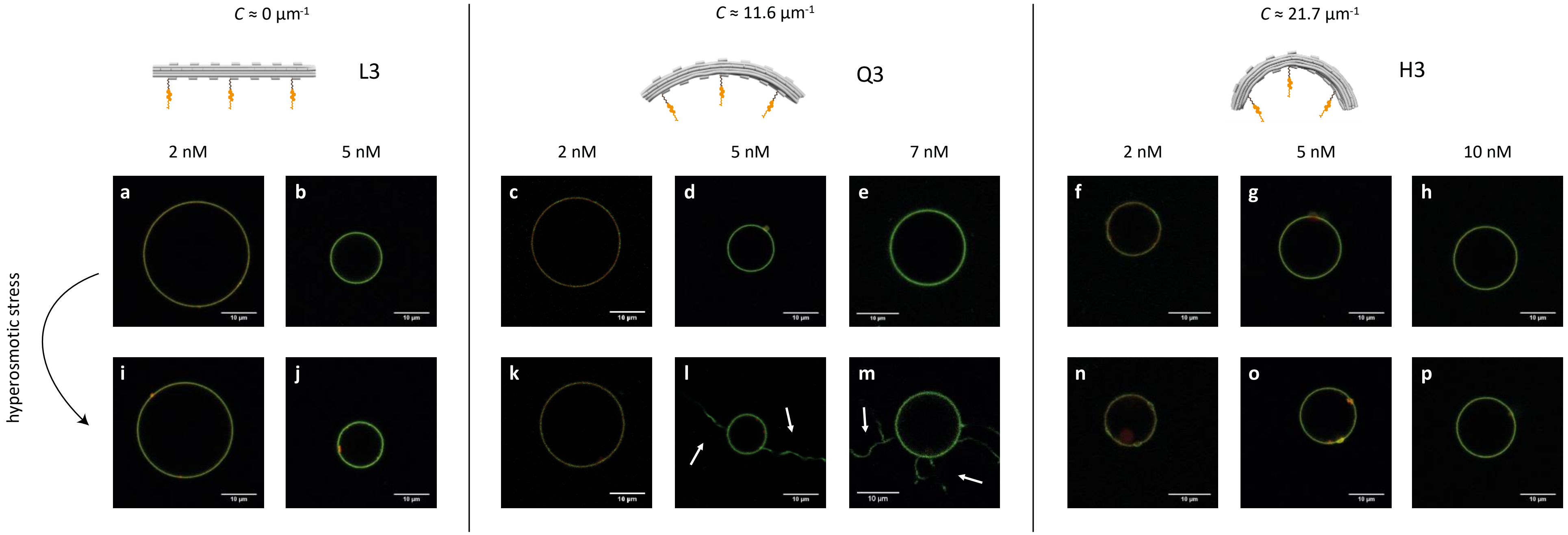

Supplementary Figure 15

Dependence on DNA origami bulk concentration and degree of curvature of DNA nanostructures with concave membrane-binding interface, for triggering membrane tubulation upon hyperosmotic stress. Structures L3, Q3 and H3 (possessing 3× TC5 anchors at positions B0, B3 and B6) bound to DOPC GUVs were imagined after at least 1h incubation period. Osmotic stress was achieved by increasing outer osmolarity in 10%. For origami structure Q3 (moderate curvature; **(c-e, k-m)**), triggering the generation of outwards positively-curved membrane tubules on GUVs upon hyperosmotic stress was dependent on the overall bulk concentration of origami utilized (at least 5 nM bulk concentration was required; deformations marked with arrows). On the other hand, origami structures L3 (no curvature; **(a-b, i-j)**) and H3 (strong curvature; **(f-h, n-p**) were not able to significantly affect the shape of vesicles upon hyperosmotic stress. Note that even at increased bulk concentrations (10 nM), highly-curved origami H3 was not able to trigger significant membrane deformation on GUVs, e.g. tubulation (panels **(h, p)**). Scale bars: 10 µm. Green fluorescence: origami labeled with Atto488; red fluorescence: membranes labeled with DOPE-Atto655.

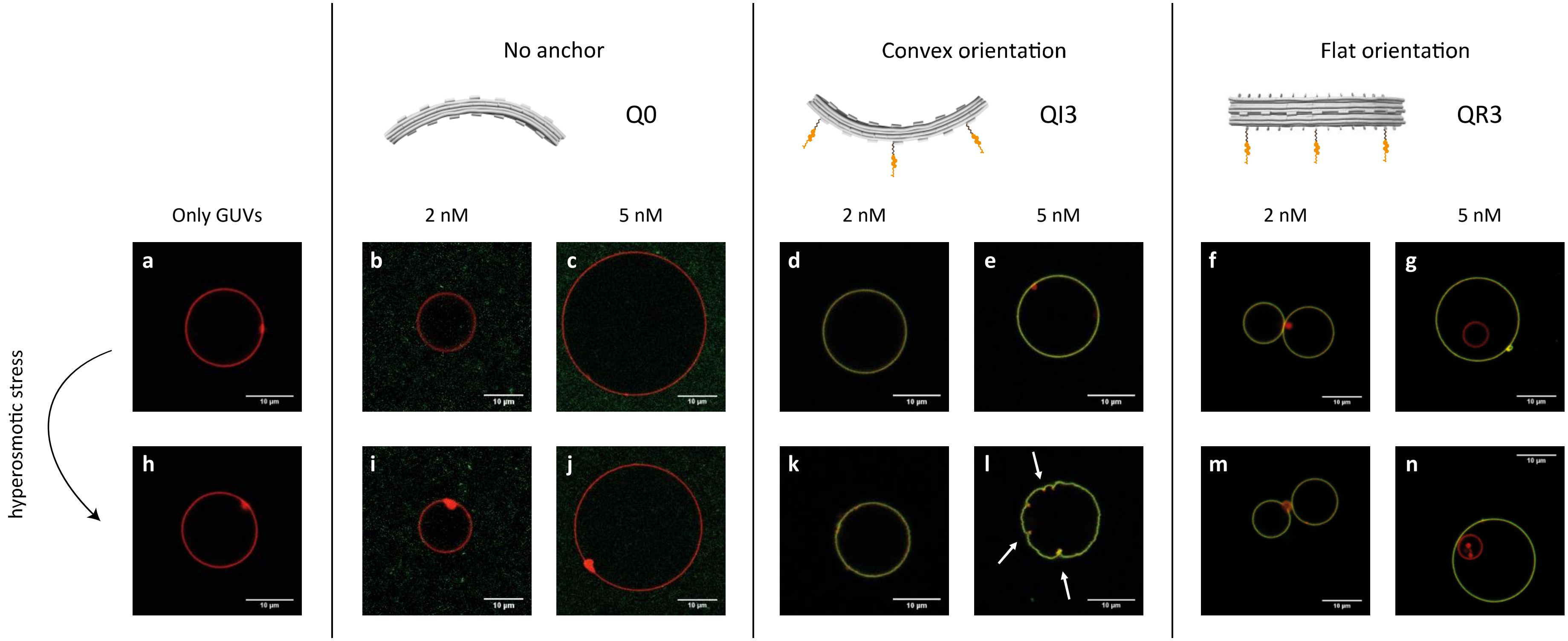

Supplementary Figure 16

Dependence on DNA origami bulk concentration and curvature of membrane anchoring origami facet, for triggering membrane deformations upon hyperosmotic stress with origami Q nanostructures. Data presented in this figure complements the previous data presented in Supplementary Fig. 15, being acquired under the same conditions. When compared with control vesicles in the absence of origami (**a, h**), no significant effect on vesicle shape was observed upon hyperosmotic stress when origami Q with no hydrophobic anchor (structure Q0) was present in solution, independently of the bulk concentration assayed (**b-c, i-j**). Regarding membrane-bound nanostructure Q variants with three TC5 anchors, for structure QR3 (displaying anchors at the non-curved origami lateral surface) no significant membrane deformations on GUVs were observed even at increased QR3 bulk concentrations (**f-g, m-n**). However, evagination/invagination-type of membranes deformations (marked with arrows) were observed for the membrane-bound convex QI3 structure (**d-e, k-l**). Similarly to what was reported for the concave Q3 structure (Supplementary Fig. 15), we also observed a dependence on the total bulk concentration of QI3 required for triggering deformations upon vesicle deflation; as shape changes only significantly happened at higher concentrations (5 nM). Scale bars: 10 µm. Green fluorescence: origami labeled with Atto488; red fluorescence: membranes labeled with DOPE-Atto655.

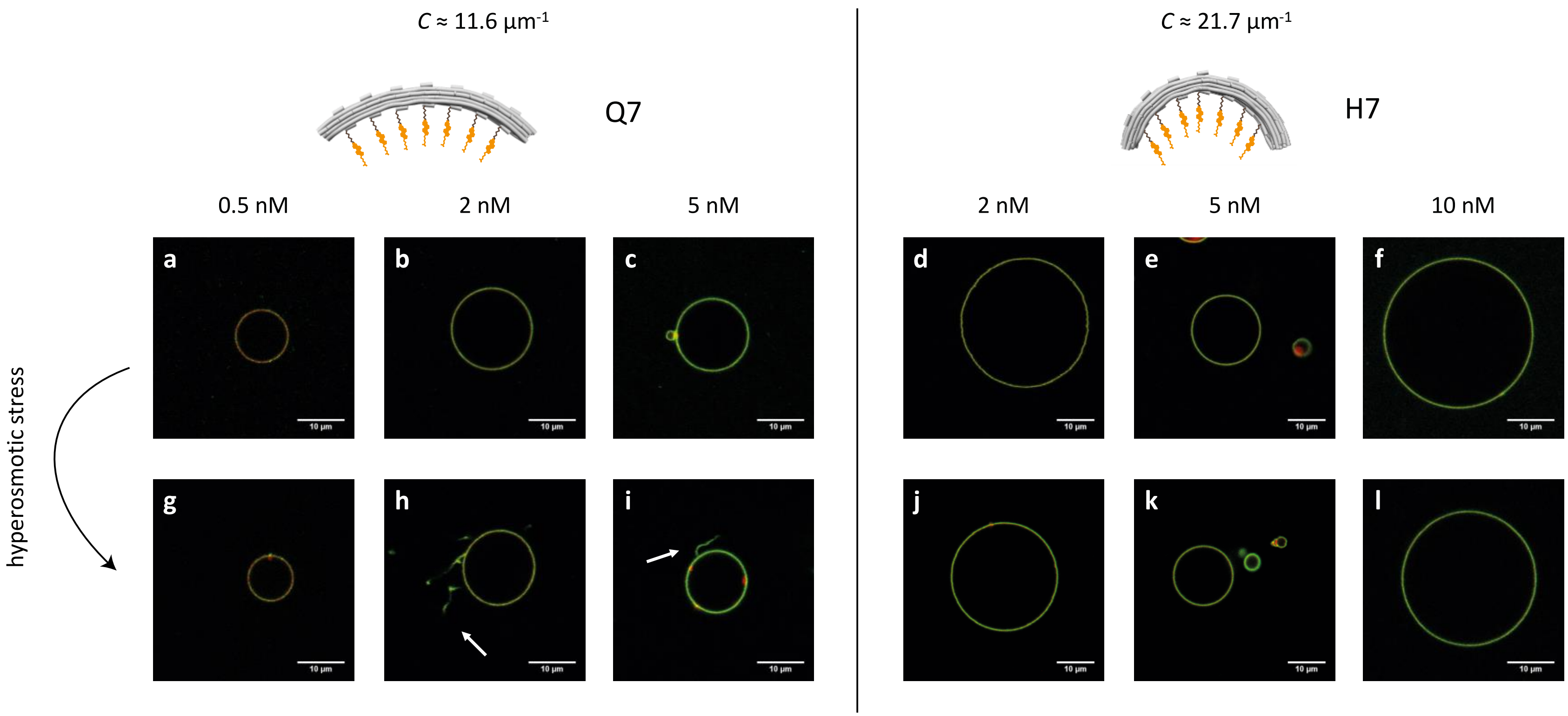

Supplementary Figure 17

Generation of membrane deformations upon hyperosmotic stress by concave nanostructures Q and H bearing increased number of TC5. Data presented in this figure complements previous data presented in Supplementary Fig. 15 and Supplementary Fig. 19, being acquired under the same conditions. The moderately-curved structure Q7 bearing 7× TC5 anchors required lower bulk concentrations for triggering membrane tubulation (marked with arrows) on GUVs (**a-c, g-i**), when compared with its counterpart bearing only 3× TC5 anchors (Supplementary Fig. 15c-e and k-m). For the highly curved DNA origami H7 bearing 7× TC5 anchors on their concave surface, on the other hand, despite the increase in the number of hydrophobic anchors resulting in a stronger membrane attachment, we were still not able to observe significant membrane deformations upon hyperosmotic stress (i.e. tubulation) on GUVs (**d-f, j-l**). Scale bars: 10 µm. Green fluorescence: origami labeled with Atto488; red fluorescence: membranes labeled with DOPE-Atto655.

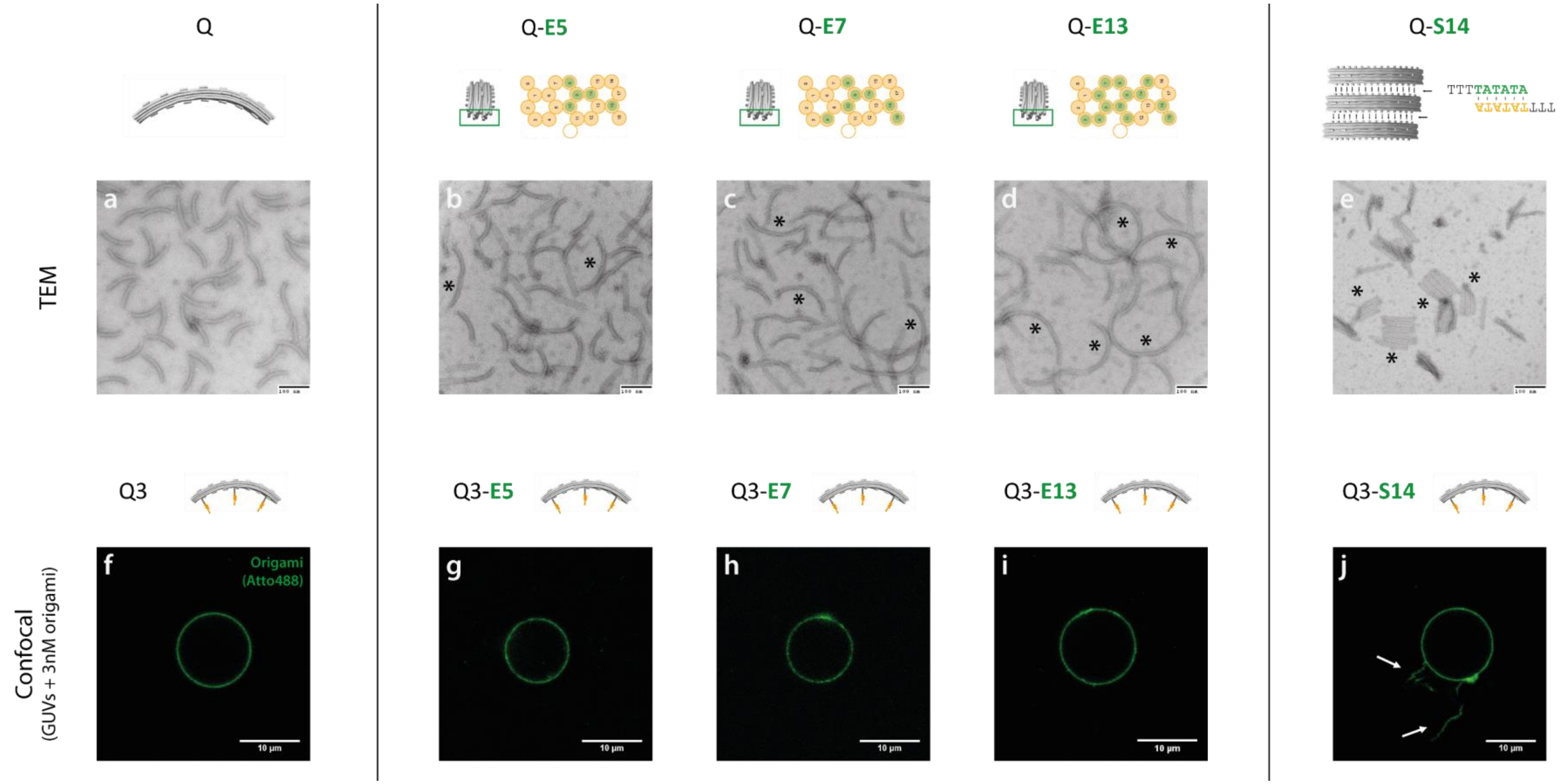

Supplementary Figure 18

Incorporation of polymerizing moieties and its effect on the ability of moderately-curved origami Q nanostructures to deform lipid vesicles. (a-e): Negative-stain TEM images of bare origami Q structure variants displaying different types and number of polymerizing moieties. Addition of polymerizing staples at the marked ends (in green; upper schemes) of monomeric structure Q **(a)** allowed for tip-to-tip blunt end stacking interactions **(b-d)**. By varying the number of blunt ends from 5 to 7 and 13, it was possible to control the size of the resulting oligomers – dimers, trimers and even tetramers (marked as stars). Polymerization could also be triggered via lateral sticky interactions **(e)**. Incorporation of 14 protrusions capable of hybridizing with their counterparts on each side of the origami Q, resulted in the formation of sheet like polymers of origami Q in solution (marked with asterisks). **(f-j)**: Variants of structure Q3 (labeled with Atto488, green) with and without corresponding polymerizing moieties were pre-incubated at 3 nM with DOPC GUVs for at least 1h and generation of membrane tubulations was followed after hyperosmotic stress. Altogether, at such intermediate DNA origami bulk concentration, we could only report significant tubulation events (marked with arrows) on GUVs upon osmotic shock were for the membrane-bound variant Q3-S14 bearing lateral polymerization strands **(j)**. Scale bars: **(a-e)** 100 nm; **(f-j)** 10 µm.

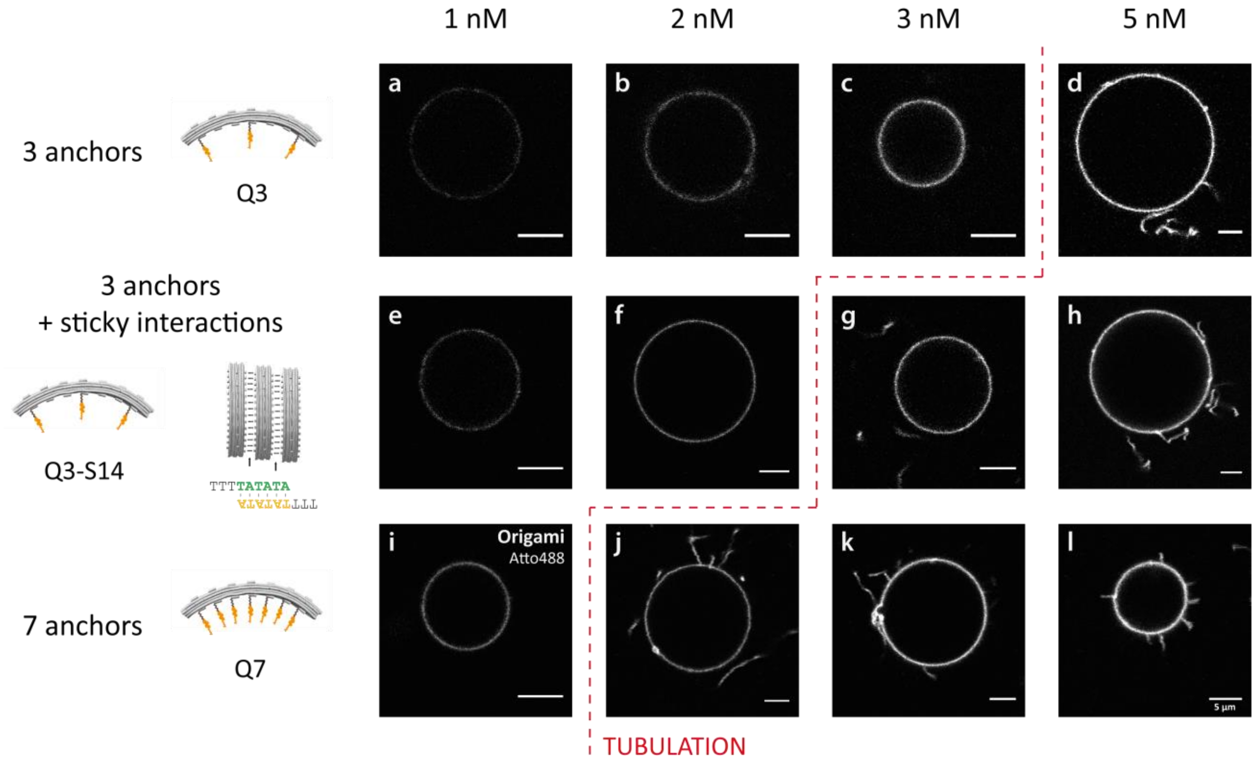

Supplementary Figure 19

Requirements for induction of membrane deformations by BAR-mimicking origami structure Q can be tuned via number of cholesteryl anchors or lateral polymerization. For structure Q3 (**a-d**), only at high bulk concentrations (5 nM) tubulation of almost all GUVs was triggered. For structure Q7 with additional cholesteryl anchors (**i-j**), the minimal bulk concentration required to trigger membrane tubulation was shifted to lower values (2 nM). Similarly for structure Q3-S14 displaying lateral sticky overhangs (**e-h**), lower total bulk concentration were necessary for triggering vesicle tubulation when compared to structure Q3 (**a-d**). Representative confocal images at the equatorial plane of GUVs incubated for at least 1 h with varying concentrations of Atto488-labeled DNA nanostructures are shown after hyperosmotic stress. The red dotted line indicates the concentration regime (total bulk concentration of origami Q) required for triggering membrane tubulation on freestanding lipid bilayers. Scale bars: 5µm.

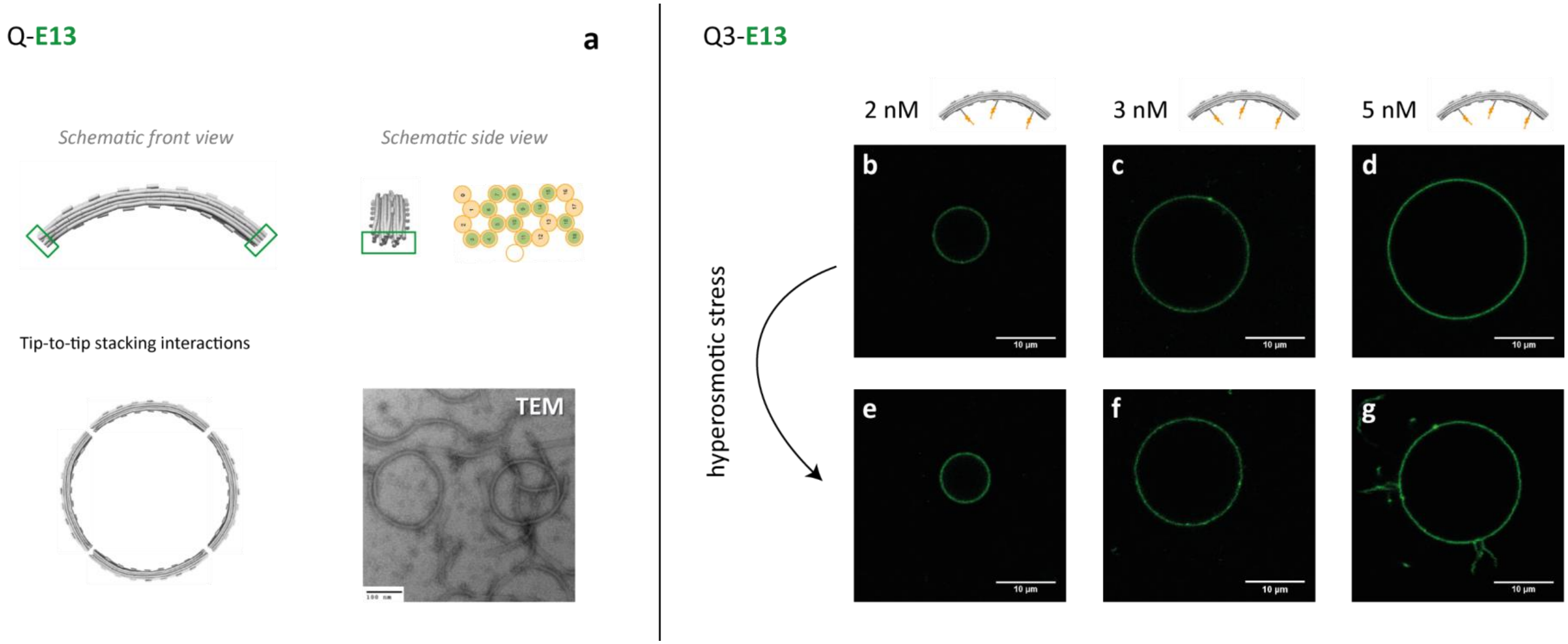

Supplementary Figure 20

Dependence on the total bulk concentration of linearly-polymerizing concave structure Q3-E13, for triggering membrane shape deformations upon hyperosmotic stress. Data presented in this figure was acquired under the same conditions as the one displayed in Supplementary Fig. 19 and Supplementary Fig. 15. **(a)** Schematic representations of the polymerization strategy based on tip-to-tip stacking interactions via 2× 13 blunt ends, based on the polymers observed by negative-stain TEM with bare structure Q-E13. **(b-g)** Similar to what we observed for the monomeric membrane-bound structure Q3 (Supplementary Fig. 19 and Supplementary Fig. 15), membrane-bound structure Q3-E13 (labeled with Atto488, green) only triggered significant membrane deformations (i.e. tubulation) upon hyperosmotic stress at high bulk concentrations (5 nM). Scale bars: **(a)** 100 nm; **(b-g)** 10 µm.

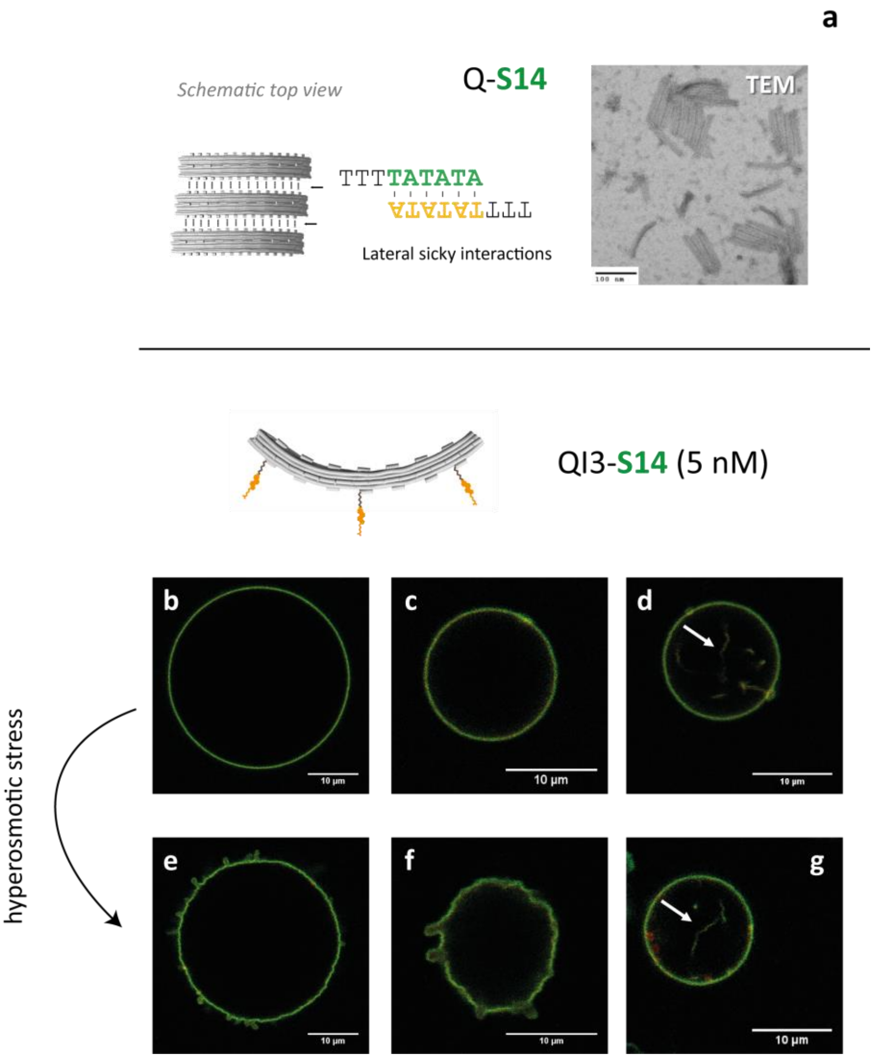

Supplementary Figure 21

GUV shape deformations triggered by membrane-bound convex variants of origami Q with lateral polymerization strands. Data presented in this figure complements previous data presented in Fig. 3 and Supplementary Fig. 16, being acquired under the same conditions. **(a)** Schematic representation of the formation of origami sheets by sticky interactions between neighboring origami structures displaying 2× 14 lateral TATATA overhangs, based on the observations by negative-stain TEM imaging of origami Q-S14. **(b-g)** For vesicles incubated with high DNA origami bulk concentrations (i.e. 5 nM) of convex polymerizing structure QI3-S14, stable evagination-like deformations were reported upon hypersosmotic (**e, f**). Additionally, a small percentage of vesicles (~15%) presented inwards tubules decorated with DNA origami (arrows), even in the absence of osmotic shock (**d, g**). Scale bars: **(a)** 100 nm; **(b-g)** 10 µm. Green fluorescence: origami labeled with Atto488; red fluorescence: membranes labeled with DOPE-Atto655.

a

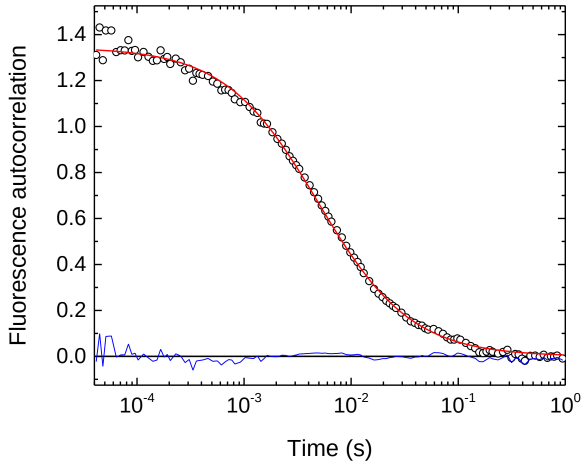

b

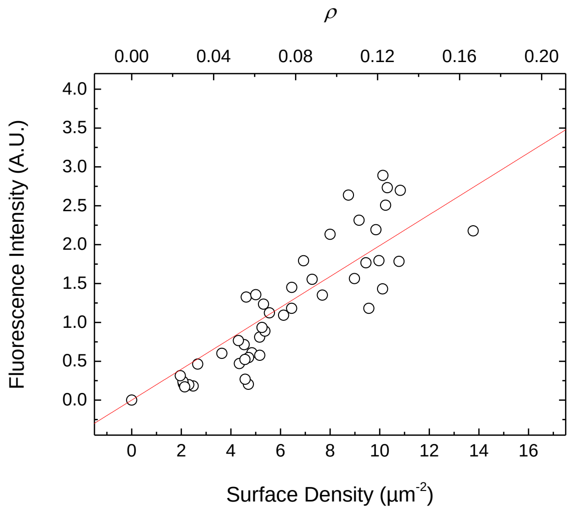

Supplementary Figure 22

Determination of DNA origami particle density at the surface of GUVs by point FCS and fluorescence intensity calibration. **(a)** Representative auto-correlation curve obtained for structure Q3 on the upper pole of a GUV at low (ρ = 0.06) surface densities. **(b)** Linear relationship (calibration curve in red) between fluorescence intensities (obtained via confocal microscopy) and surface densities (determined for $\rho < 0.2$, where the one-component two-dimensional diffusion model (equation 2) is valid) recovered for membrane-bound DNA origami on individual GUVs by FCS (open circles).

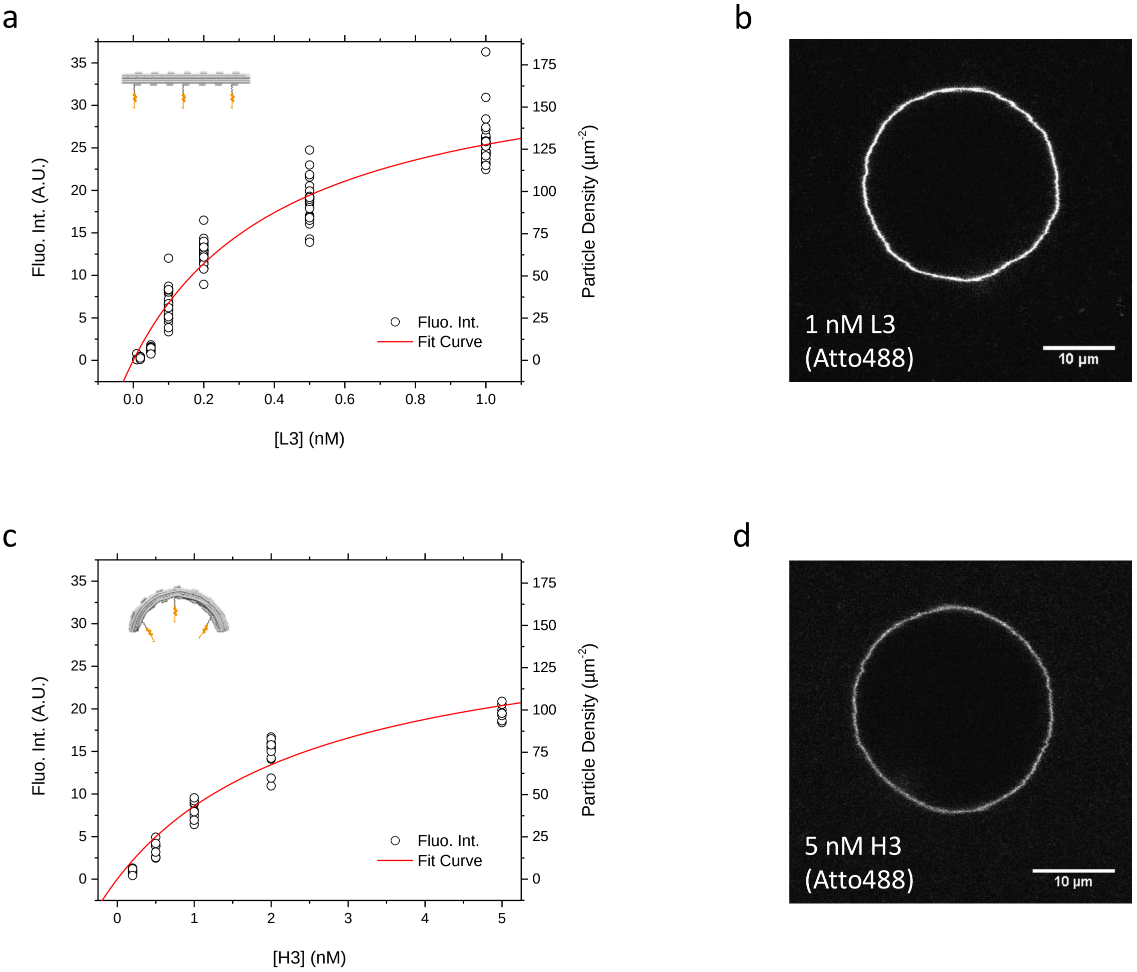

Supplementary Figure 23

Attachment of origami structures L3 and H3 to GUVs upon overnight incubation. (a, c) Representative membrane binding curves obtained using a Langmuir isotherm fit (equation 1) for the flat origami structure L3 (**a**) and the highly-curved origami structure H3 (**c**). Each data point corresponds to the fluorescence intensity at the membrane level of DNA origami structures (labeled with 3× Atto-488 dyes) for single GUVs. **(b, d)** Shape variations observed for GUVs incubated overnight with structures L3 and H3. Even at high densities of membrane-bound DNA origami (≥ 100 particles per µm^2, estimated from the fluorescence brightness via calibration curve of Supplementary Figure 22b), no tubulation events were observed for GUVs incubated with the flat structure L3 (**b**) or highly-curved structure H3 (**d**). Unlike the GUVs incubated with moderately-curved structure Q3 (Fig. 4c), GUVs with membrane-bound structures L3 and H3 displayed, at best, increased flaccid membrane deformations. Scale bars: 10 µm.

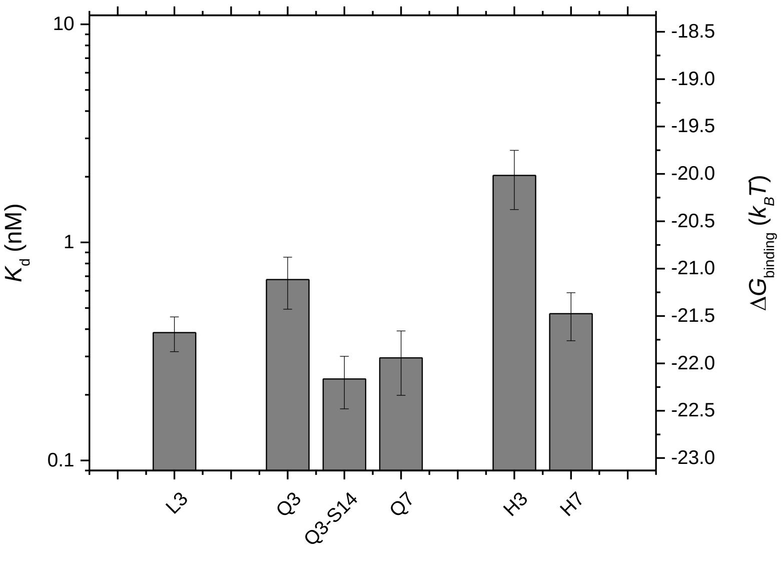

Supplementary Figure 24

Apparent dissociation constants (K_d) recovered from the analysis of membrane binding of different variants of curved DNA origami scaffolds. Affinity of various cholesteryl-modified BAR-mimicking DNA nanostructures to DOPC GUVs was determined after overnight incubation, from the analysis of the fluorescence intensity of membrane-bound DNA origami as a function of total bulk concentration. Representative binding curves obtained and respective Langmuir isotherm fits (equation 1) are depicted in Fig. 4a and Supplementary Fig. 23. Values correspond to the average K_d (± st. dev.): L3 (n_{total} = 288 GUVs; n = 131-157 GUVs per fit, 2 repeats), Q3 (n_{total} = 277 GUVs; n = 83-100 GUVs per fit, 3 repeats), Q3-S14 (n_{total} = 84 GUVs; n = 36-48 GUVs per fit, 2 repeats), Q7 (n_{total} = 117 GUVs; n = 53-64 GUVs per fit, 2 repeats), H3 (n_{total} = 106 GUVs; n = 48-58 GUVs per fit, 2 repeats) and H7 (n_{total} = 103 GUVs; n = 47-56 GUVs per fit, 2 repeats). $\Delta G_{binding}$ was calculated via $\Delta G = RT\ln K_d$.

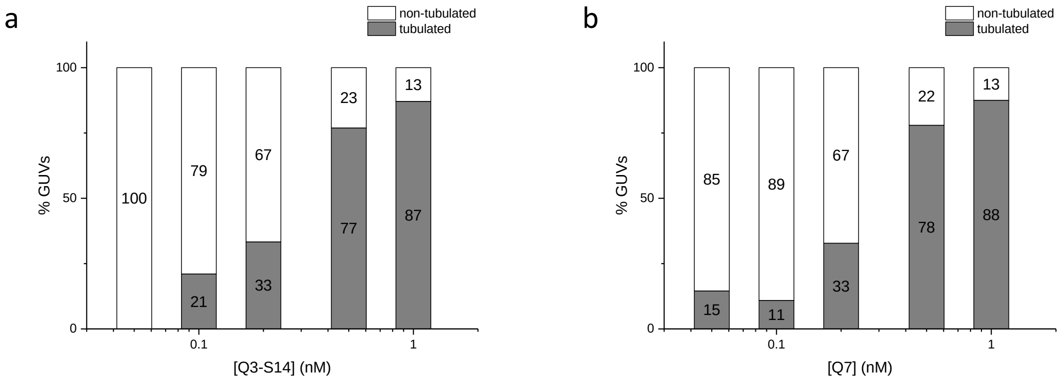

Supplementary Figure 25

Efficiencies of vesicle tubulation for origami structure Q3-S14 bearing lateral polymerization overhangs and origami structure Q7 possessing additional cholesteryl anchors. Percentages of GUVs with (closed bars) and without (open bars) outwards tubules observed after overnight incubation at different total bulk concentrations of DNA origami structures Q3-S14 (**a**; n_{total} = 118 GUVs; n = 13-31 GUVs per origami concentration) and Q7 (**b**; n_{total} = 297 GUVs; n = 55-64 GUVs per origami concentration).

Supplementary Table 1

List of DNA staples - Origami L

Start	*End*	*Sequence*	*Length*	*Type*	*Pos.*
00[031]	03[027]	CTTAATGCGCCAGCACGTTTCCTCGGATTAAA	32	#0_core	
00[053]	06[049]	CGTAACCACCACGAAGTATTAGACTTTTAAAAG	33	#0_core	
00[073]	06[070]	AGTGTAGCGGTTCAATTACCTGAATAATTTCA	32	#0_core	
00[094]	06[091]	GCGGGCGCTAGTTTGAAATACCGTTAGAAAAA	32	#0_core	
00[115]	06[112]	GAAGGGAAGAAATTCCAAGAACGAGATCGTAG	32	#0_core	
00[136]	06[133]	CCGGCGAACGTCATTAGACGGGAGAAGAGATA	32	#0_core	
00[157]	06[154]	AACCGTCTATCGCGACATTCAACAGATCACCG	32	#0_core	
00[178]	06[175]	CTCCAACGTCAACCAGAGCCGCCGTAACAAAT	32	#0_core	
00[199]	06[196]	TCCACTATTAAAGGTTTAGTACCAGATTTTCA	32	#0_core	
00[220]	06[217]	TGTTCCAGTTTTCTTAAACAGCTATTATATTC	32	#0_core	
00[241]	06[238]	GCCCGAGATAGAGCCGGAACGAGTTATGAACG	32	#0_core	
00[262]	06[259]	CCCTTATAAATCCACATTCAACTGCTCATAAC	32	#0_core	
00[283]	06[280]	TGGTTCCGAAAAACAGGTCAGGAAATTAGAGC	32	#0_core	
00[304]	06[301]	GGCGAAAATCCTGACCCTGTAATTTTCATATA	32	#0_core	
00[325]	06[322]	CCACGCTGGTTGCATTAAATTTTTTTCTGGCC	32	#0_core	
00[346]	01[346]	GAGAGAGTTGCTGAGACGGGCA	22	#0_core	
02[034]	07[048]	TTGTGCTATAACGACGCAAATAGAAGAACTCAAACTAGTAAC	42	#0_core	
02[346]	02[329]	GGCGCCAGGGTGGTTTTT	18	#0_core	
03[028]	03[063]	GGGATTTTAGACCCGTCAAACAATTCGACAACTACA	36	#0_core	
03[064]	03[105]	AAATAAGATGATGAAACATAATTTCGATAAATAAGGCGTCCA	42	#0_core	
03[106]	03[147]	ATCAAACCAAGTACCGCACAGAGAGCTGAACACCCTGAAATA	42	#0_core	
03[148]	03[189]	TGGTGGGAGGGAAGGTAAACCACCCTTGACAGGAGGTTGGCC	42	#0_core	
03[190]	03[231]	CGGATCAGAACCGCCACCCGGTTTAGATAGTTGCGCCGATCG	42	#0_core	
03[232]	03[273]	AAATCGGTCAATCATAAGGAAAGATATACATAACGCCAATCA	42	#0_core	
03[274]	03[315]	AAGCGTACCTTTAATTGCAAAGCTACGGGAGAAGCCTTTATT	42	#0_core	
03[316]	03[346]	GTAATCAGCTCATTTTTTTAATGAATCGGCC	31	#0_core	
04[048]	10[028]	TTTGCAGAGAGGAACGGTACGTCTGTCCTTGCTCA	35	#0_core	
04[090]	04[049]	AACACTAGTAACATCAAGAAAACAAAAGTTCGTATTAAATCC	42	#0_core	
04[132]	04[091]	GTAATTTTACTCATCGAGAACAAGCAGAAATAAATAAGAATA	42	#0_core	
04[174]	04[133]	CGATTAGCCATATTGACGGAAATTATATTCCAAAGTCAGAGG	42	#0_core	
04[216]	04[175]	CCATCGTATCTCAGAACCGCCACCCTTATAAGGCAGGTCAGA	42	#0_core	
04[258]	04[217]	GGCATGGTAGGAACCGAACTGACCAATGTGCAATGACAACAA	42	#0_core	
04[300]	04[259]	GGATAGCATTCCTTTTGATAAGAGGTAGCTAAGGAATTACGA	42	#0_core	
04[342]	04[301]	CAGCTGCATAACCAATAGGAACGCCATTAAATTTCAACGCAA	42	#0_core	
05[049]	05[048]	CGTTATTGGAGGAGCGAATTGAGGAAGGATATAGATTCCGAA	42	#0_core	
05[070]	05[069]	TTACATTCGATGTGAGCCTTTTACATCGAAAATACCAATTAA	42	#0_core	
05[091]	05[090]	TCATAATAAAAAGCCATGCAAATCCAATTATATATTTCGGAA	42	#0_core	
05[112]	05[111]	TTTTTATAAGCTTATCAGATAAGTCCTGTAGCATGTAAGCCG	42	#0_core	
05[133]	05[132]	GCTAATAGAAATGAAAAATCCAAATAAGTTAGCAGCCTGAGC	42	#0_core	
05[154]	05[153]	AAAGGTGGCCAGTAGCAAGAAACGCAAATCATAGAAATCATT	42	#0_core	
05[175]	05[174]	TGATATTAGTACCGTTACCACCGGAACCAACCCTCAGGGCCT	42	#0_core	
05[196]	05[195]	CCACCACGATTTCGTCGGGGTTTTGCTCCAATATAAGCAGAG	42	#0_core	
05[217]	05[216]	CGCATAAAATCGTCACTTTTTTCACGTTCCTTTAATTGCCCA	42	#0_core	
05[238]	05[237]	AAAGAGGTCTAATCTTATACCAAGCGCGCCGATAAATCTTTG	42	#0_core	
05[259]	05[258]	GAGCAACAGTTTTGCATGGGAAGAAAAAACTATTACAAGTAA	42	#0_core	
05[280]	05[279]	TTGCGGACATGCAACTTCAAAAAGATTAACTAATTCGCATTT	42	#0_core	
05[301]	05[300]	TTTTAGACAAGGGTGAAAATCATACAGGTGCCTCAGAAAAAT	42	#0_core	
05[322]	05[321]	AATAATTTCCAACCCGCCGGTTGATAATACCAAATATTCAAA	42	#0_core	
06[048]	00[032]	TTTGAACAACTACAGGATTTAACCCGCCGCG	31	#0_core	
06[069]	00[054]	TTTGATGCTTTGTTATTCATTCACGCTGCG	30	#0_core	
06[090]	00[074]	GCCTGTTTCAAAACTTAATGGGGCGCTGGCA	31	#0_core	
06[111]	00[095]	GAATCTTTACGACTCCTTATCAGCGAAAGGA	31	#0_core	
06[132]	00[116]	ACCCATGAAAATTCGGAAGCGGGCGAGAAAG	31	#0_core	
06[153]	00[137]	TCACCACAATCAAGCAAAAGGACGGGGAAAG	31	#0_core	
06[174]	00[158]	AAATCACCGCCATTAACCACCAAGGGCGAAA	31	#0_core	
06[195]	00[179]	GGGATAGGGTTGCAACTCAGGAGAACGTGGA	31	#0_core	
06[216]	00[200]	GGTCGAGGAGCCTCGTGAATTGGAACAAGAG	31	#0_core	

06[237]	00[221]	GTGTAATCGCCTGAGTTACTTGGTTGAGTGT	31	#0_core	
06[258]	00[242]	CCTCGACAACATAGAGGAATACAAAAGAATA	31	#0_core	
06[279]	00[263]	TTAATCGCGTTTTAAAACTCCTCGGCAAAAT	31	#0_core	
06[300]	00[284]	TTTTAAATAAAGGCAACATTATGTTTGATGG	31	#0_core	
06[321]	00[305]	TTCCTGTATAAGCCAAAATTCTGCCCCAGCA	31	#0_core	
06[342]	00[326]	TCACATTAATTGCGTCACCAGAGCAAGCGGT	31	#0_core	
07[021]	04[021]	CCTGAGTTAACCGTAAAAGAGCCAGAAT	28	#0_core	
07[049]	07[090]	ATTATCATTTTGTGATATTACCTTTTTTAATGGACTTTTTAG	42	#0_core	
07[091]	07[132]	TATCATATGCGTCTAAATTACCGCGCCCAATAGAAAACAAGA	42	#0_core	
07[133]	07[174]	ATTGAGTTAAGCTGTCGACTTGAGCCATTTGGGCAGACTCAT	42	#0_core	
07[175]	07[216]	TAAAGCCAGAATCGAGAGCAAGCCCAATAGGAACAAACTGAG	42	#0_core	
07[217]	07[258]	GCTTGCAGGGAGTATCCAGACCAGGCGCATAGGCGGATTTAC	42	#0_core	
07[259]	07[300]	CAGACGACGATAATATTGCTGAATATAATGCTGAAGCAATGC	42	#0_core	
07[301]	07[342]	AATGCCTGAGTAGATTGTAGCCAGCTTTCATCACATAAAGTG	42	#0_core	
08[048]	08[021]	ACTAGGAGCACTATCGGCCTTGCTGGTA	28	#0_core	
08[090]	08[070]	TACGAGAAAAAACAGTACATA	21	#0_core	
08[132]	08[112]	GCAACGTCACAAGCAAATCAG	21	#0_core	
08[174]	08[153]	CTCCACCCTAATTAGAGCCAGC	22	#0_core	
08[216]	08[196]	TTAAGGCTCCCCATGTACCGT	21	#0_core	
08[258]	08[238]	GCGAACTAACTGGCTGACCTT	21	#0_core	
08[300]	08[280]	AGCAAAATTTAGCTCAACATG	21	#0_core	
08[347]	11[347]	ATACGAGCCGGATCCTGTGATTCGTAATGCCTGCAGGT	38	#0_core	
09[021]	15[048]	AACAGGAAAAACATAAGAATAATAGCCCTAAAAGGTCGTTTG	42	#0_core	
09[063]	09[062]	AACGGTCAATATCATAGAATCCTTGAGTCAGATATAAACAAT	42	#0_core	
09[105]	09[104]	TAATAATAGAAGACGGAGGTTTTGAACAGAACGGCAGAAAAA	42	#0_core	
09[147]	09[146]	ATAAGAAATCACCTTAGCAGCACCGTCATACATGACCACGGA	42	#0_core	
09[189]	09[188]	GATAACACTGAGAAAGTTAGCGTAACCCTCAAGGTCCAGGCG	42	#0_core	
09[231]	09[230]	AACGGTCAAGAGTACATTCAGTGAATCACTCATCAAAAGTAC	42	#0_core	
09[273]	09[272]	GAAAGTTAAATAAGATTCTGCGAACGATAGTCAATGAAGCCC	42	#0_core	
09[315]	09[314]	CAAAACGAGTAAATGTCACGTTGGTGAACTAGCGTAAAGCCC	42	#0_core	
10[027]	11[048]	TGGAAATGATTATTTACATTGGCAGATGAAAGGGA	35	#0_core	
11[070]	11[090]	AATAACCTTGCTATGCTGAAC	21	#0_core	
11[112]	11[132]	GTATTCTAAGAATTATCCCTA	21	#0_core	
11[154]	11[175]	CATTACCATTAGCAGAGCCCCA	22	#0_core	
11[196]	11[216]	CAGTACAAACTAATAATAACC	21	#0_core	
11[238]	11[258]	CAAGAACCGGATAGGACGTAA	21	#0_core	
11[280]	11[300]	AGTACGGTGTCTATCCAATGA	21	#0_core	
11[322]	05[342]	GGATTCTCCGTGGCATCATGGCGCGTTGCGCTCAC	35	#0_core	
12[048]	18[028]	AGATGCAGTTTCACCAGTCACCCTGAAATATATTA	35	#0_core	
12[090]	12[049]	AATCGGTAATCTGTAAATCGTCGCTACAGTTTCCTGATTATC	42	#0_core	
12[132]	12[091]	TTTTTTTATCGCGAGGCGTTTTAGCGTTATGGCTTAATTGAG	42	#0_core	
12[174]	12[133]	ATGATGAACCAAGGCCGGAAACGTCAGCAATTACCGAAGCCC	42	#0_core	
12[216]	12[175]	ACGAGGCGACAACGCCTGTAGCATTCTTAGACATGGCTTTTG	42	#0_core	
12[258]	12[217]	AGTAAAGTCATTCATTACCCAAATCACCCCGACAGCATCGGA	42	#0_core	
12[300]	12[259]	ATCAATAACGGAAGTTTCATTCCATAAGCGAGAGGGGGTAAT	42	#0_core	
12[335]	12[301]	TTGGAACAAACGGCGGATTTCATAGTCAAATCACC	35	#0_core	
13[049]	13[048]	AATTCATGACTACCATCTGAACCTCAAAGCAAATCAAATGGC	42	#0_core	
13[091]	13[090]	TTTAACAGAAAAGTACTCTGAGAGACTACCACTATATCCATA	42	#0_core	
13[133]	13[132]	AAGTAAGGCAATACCCTAACGAGCGTCTTGAGCCATAAAGAA	42	#0_core	
13[175]	13[174]	AGTGTACACGTTAATGCGGTCATAGCCCTCATCACCGACAGG	42	#0_core	
13[217]	13[216]	CAACGGCCGGTAAAATAACTTTCAACAGTCAGGAATTGGTAG	42	#0_core	
13[259]	13[258]	TTAGACTAAATGCTTTCTTTAATCATTGTGTTATACCAATGT	42	#0_core	
13[301]	13[300]	TATTCAAAACAAAGGCATTTGGGGCGCGCCGTAGCATTATGA	42	#0_core	
14[048]	14[049]	TGATTAGTTGGCGTCTAAAATATCTTCACCAGAAGATAATCC	42	#0_core	
14[090]	14[091]	TAATTTTATATACTAGACAAAGAACGCCAGTATCACAACATG	42	#0_core	
14[132]	14[133]	ACAAAAATAAACCGGATTTTTTGTTTAAGAAACCATAGCCGA	42	#0_core	
14[174]	14[175]	TTTAAAATCAAAGACCCTCAGAGCCGCTGAATTTAAATAAGT	42	#0_core	
14[216]	14[217]	TGAGGCAACTAAATATCTCCAAAAAATGCGGGAACGAGGCTT	42	#0_core	
14[258]	14[259]	AATACTGGCTCACTCGTTAATAAAACGAGAGGCAGAGCGTCC	42	#0_core	
14[300]	14[301]	GATAAAATAGTACAGCAAAGAATTAGATTCAAAGATCTAGCT	42	#0_core	
14[342]	08[322]	TAAGTTGGGTAAAGAGCTGTTAGACATTAAATGTG	35	#0_core	
15[021]	12[021]	CGAACTGCGTGGCACTTCTGAACGACCA	28	#0_core	
15[049]	15[090]	GATTATACTTCTTAACAAACATAGCGATAGCTTTGGGTAGGC	42	#0_core	
15[091]	15[132]	AGAGGCATTTTCAATGGCCTTAAATCAAGATTAACAAGTTAC	42	#0_core	

15[133]	15[174]	CAGAAGGAAACCAATAAATCAGTAGCGACAGAATCATCGGGG	42	#0_core	
15[175]	15[216]	TCAGTGCCTTGAGACTGATCTAAAGTTTTGTCGGGAAACTAA	42	#0_core	
15[217]	15[258]	AGACTTTTTCATAAAAAAGGCTTGCCCTGACGAGAACTGCGG	42	#0_core	
15[259]	15[300]	AATCGTCATAAATATTAGTAGATTTAGTTTGACTACTATTAA	42	#0_core	
15[301]	15[342]	TGCCGGAGAGGGGTAATAGATGGGCGCATCGTAATTACGCCA	42	#0_core	
16[062]	16[021]	TTGAATAATGGAAGGGAATATCTCATCGCCATTAAAAATACC	42	#0_core	
16[104]	16[063]	CTGAGCCAGTAATAAGCTTAGGTAGATTAAGACGCTGTCAGG	42	#0_core	
16[147]	16[105]	GAAGAGGAAACGCAATAGCCAGTTGTTGCTATTTTGCATTCAG	43	#0_core	
16[189]	16[148]	TGAGTAACAGTGCCCGTCATCTTTTCAAGTTTGCCTTTCGTA	42	#0_core	
16[231]	16[190]	ACTGAGGAAGTTTCCATATAGAAATCTTTCCAGACGTTAGGC	42	#0_core	
16[273]	16[232]	GACTATTCATTGAATCCATTTTAAGAAACACCAGAACGATAC	42	#0_core	
16[315]	16[274]	ATCTAGCTATTTTTGAGTCAATTCCATTAGATACATTTCCCT	42	#0_core	
16[347]	19[347]	CGGGCCTCTTCGGCCATTCCCGGAAACAGGAAGATCGC	38	#0_core	
17[021]	17[034]	TGAGGCGGTCAGTC	14	#0_core	
17[329]	16[316]	GCCATTCCTACCGTGCATCTGCCGGTA	27	#0_core	
18[027]	19[062]	ACACCGCAATGAAAAATCTAAAGCATCACCTTGATCAAAATTATTTGCA	49	#0_core	
18[062]	18[063]	AAATAAACAGAATATATTAATTAATTTTCCTCAATAGAACAG	42	#0_core	
18[104]	18[105]	AGACGACACCGCCTGTAACCTCCCGACTTTACAATTTTGTCC	42	#0_core	
18[146]	18[147]	TTATTACCAAAAGGTGCCAATGAAACCACCGACTGTAACTCC	42	#0_core	
18[188]	18[189]	TTCTGAATGAGAAGGACACAGACAGCCCTTGAATTTTTATTA	42	#0_core	
18[230]	18[231]	CCTAAAATACTTTGACACGTAACAAAGCTGAATTGGGACCAA	42	#0_core	
18[272]	18[273]	ATAAATCGGGAAGCAATAACAGTTGATTAGGGTCAATTGACC	42	#0_core	
18[314]	18[315]	AGCAAACCCATGTCAAGACCGTAATGGGAAGGGGACGTCTGG	42	#0_core	
19[063]	19[104]	CGTAATGAATTTATCAAAATCATAGGCGACAAAAGGTAAAGT	42	#0_core	
19[105]	19[146]	AATTCTATCCTGAATCTTACCAACGCAAAAGAACTGGCATGA	42	#0_core	
19[147]	19[188]	TTAAGGCGCGTTTTCATCGGCATTTTCCCCCTGCCTATTTCG	42	#0_core	
19[189]	19[230]	GAACCCTGTATGGGATTTTGCTAAACACGTAATGCCACTACG	42	#0_core	
19[231]	19[272]	AAGGCCTTGAGATGGTTTAATTTCAAAAACAGTTCAGAAAAC	42	#0_core	
19[273]	19[314]	GAGAAAACCTGTTTAGCTATATTTTCTATCAGGTCATTGCCT	42	#0_core	
19[315]	12[336]	GAGAGACGACAGTATCGGCCTCCAGGCAATCGCCAGGGTTTTGCCAAGC	49	#0_core	
11[049]	11[069]	ATTATCATCATAAACAGTATG	21	#1_bottom	B0
11[091]	11[111]	GCTCAACAGTAGCAACAATCG	21	#1_bottom	B1
11[133]	11[153]	GCAATAGCTATCCATATAAAC	21	#1_bottom	B2
11[176]	11[195]	GTAAGCGTCATGATTAGCAC	20	#1_bottom	B3
11[217]	11[237]	TCAGCAGCGAAAAGCGATTGA	21	#1_bottom	B4
11[259]	11[279]	AGAAGTTTTGCCGATTGCAAA	21	#1_bottom	B5
11[301]	11[321]	AAGGCCGGAGACATGTACCTC	21	#1_bottom	B6
08[069]	08[049]	AAATTCGCCCGGAACAAAGAA	21	#2_top	T0
08[111]	08[091]	ATTCCCATCTATACAAATTCT	21	#2_top	T1
08[152]	08[133]	ATTTATTTCCAATAATAAGA	20	#2_top	T2
08[195]	08[175]	AAGTGCCGTGGAAAGCGCAGT	21	#2_top	T3
08[237]	08[217]	CAAGATTTGTTAAAGGCCGCT	21	#2_top	T4
08[279]	08[259]	TTACTTCAAAAAACCAAAATA	21	#2_top	T5
08[321]	08[301]	AGACAGGAAATGTGTAGGTAA	21	#2_top	T6
02[055]	02[035]	AATTTACAATAGATAATACAT	21	#3_left	L0
02[076]	02[056]	AAGCAAAAGCGCGCAGAGGCG	21	#3_left	L1
02[097]	02[077]	CTACCGTGTATCTTCTGACCT	21	#3_left	L2
02[118]	02[098]	ACGGTATTAATAATCGGCTGT	21	#3_left	L3
02[139]	02[119]	AAGAATTAAAATAACATAAAA	21	#3_left	L4
02[160]	02[140]	CCCGATTGATTACCAGCGCCA	21	#3_left	L5
02[181]	02[161]	CCGCCAGCATCAGAGCCGCCA	21	#3_left	L6
02[202]	02[182]	CGGCCACCCATAGGTGTATCA	21	#3_left	L7
02[223]	02[203]	CCTGATACCTCAGCTTGCTTT	21	#3_left	L8
02[244]	02[224]	ATGCGCAGACCGCGACCTGCT	21	#3_left	L9
02[265]	02[245]	AAAATGCAGTCATCAGTTGAG	21	#3_left	L10
02[286]	02[266]	CATTAGAGAGAACCAGACCGG	21	#3_left	L11
02[307]	02[287]	TGACTTTTGAATCGGTTGTAC	21	#3_left	L12
02[328]	02[308]	CTTGTTAAAACGTTAATATTT	21	#3_left	L13
17[035]	17[055]	AAACCCTCAATCTTAGAACAA	21	#4_right	R0
17[056]	17[076]	TTGCGTAGATTTAGAAGAGTT	21	#4_right	R1
17[077]	17[097]	TTTAACCTCCGGAGAATATCA	21	#4_right	R2
17[098]	17[118]	ATAAACAACATGCCCAGCTCC	21	#4_right	R3
17[119]	17[139]	AGAGCCTAATTTATAACGGAG	21	#4_right	R4
17[140]	17[160]	TATGTTAGCAAAAGCGTCATT	21	#4_right	R5

17[161]	17[181]	ATTAGCGTTTGCATAAACAAT	21	#4_right	R6
17[182]	17[202]	GAAAGTATTAAGAGTAAATTC	21	#4_right	R7
17[203]	17[223]	AGCGGAGTGAGATAAACGGAA	21	#4_right	R8
17[224]	17[244]	AGAGGCAAAAGAAGTAGTAAA	21	#4_right	R9
17[245]	17[265]	TTACCTTATGCGCCCTCAAAA	21	#4_right	R10
17[266]	17[286]	ATCAGGTCTTTACGCAAATCT	21	#4_right	R11
17[287]	17[307]	GAAAAGGTGGCAAGATCTAGA	21	#4_right	R12
17[308]	17[328]	GAATCGATGAACAGTTTGAGC	21	#4_right	R13
00[020]	00[005]	GCTACAGGGCGCGTAC	16	#5_ends	E0
00[361]	00[347]	TCACCGCCTGGCCCT	15	#5_ends	F0
01[005]	01[020]	TATGGTTGCTTTGACG	16	#5_ends	E1
01[347]	01[361]	ACAGCTGATTGCCCT	15	#5_ends	F1
02[020]	02[005]	TTAGAATCAGAGCGGG	16	#5_ends	E2
02[361]	02[347]	GCGGTTTGCGTATTG	15	#5_ends	F2
03[005]	03[020]	AGCTAAACAGGAGGCC	16	#5_ends	E3
03[347]	03[361]	AACGCGCGGGGAGAG	15	#5_ends	F3
04[020]	04[002]	CCTGAGAAGTGTTTTTATA	19	#5_ends	E4
04[358]	04[343]	GGGAAACCTGTCGTGC	16	#5_ends	F4
05[002]	05[020]	ATCAGTGAGGCCACCGAGT	19	#5_ends	E5
05[343]	05[358]	TGCCCGCTTTCCAGTC	16	#5_ends	F5
06[020]	06[002]	TGTAGCAATACTTCTTTGA	19	#5_ends	E6
06[358]	06[343]	TAATGAGTGAGCTAAC	16	#5_ends	F6
07[002]	07[020]	TTAGTAATAACATCACTTG	19	#5_ends	E7
07[343]	07[358]	TAAAGCCTGGGGTGCC	16	#5_ends	F7
08[020]	08[005]	ATATCCAGAACAATAT	16	#5_ends	E8
08[361]	08[348]	CAATTCCACACAAC	14	#5_ends	F8
09[005]	09[020]	TACCGCCAGCCATTGC	16	#5_ends	E9
09[343]	09[361]	TGAAATTGTTATCCGCTCA	19	#5_ends	F9
10[020]	10[005]	ACCTACATTTTGACGC	16	#5_ends	E10
10[361]	10[343]	CCCCGGGTACCGAGCTCGA	19	#5_ends	F10
11[005]	11[020]	TCAATCGTCTGAAATG	16	#5_ends	E11
11[348]	11[361]	CGACTCTAGAGGAT	14	#5_ends	F11
12[020]	12[002]	GTAATAAAAGGGACATTCT	19	#5_ends	E12
12[358]	12[343]	TAAAACGACGGCCAGT	16	#5_ends	F12
13[002]	13[020]	GGCCAACAGAGATAGAACC	19	#5_ends	E13
13[343]	13[358]	CCCAGTCACGACGTTG	16	#5_ends	F13
14[020]	14[002]	CAGACAATATTTTTGAATG	19	#5_ends	E14
14[358]	14[343]	TGTGCTGCAAGGCGAT	16	#5_ends	F14
15[002]	15[020]	GCTATTAGTCTTTAATGCG	19	#5_ends	E15
15[343]	15[358]	GCTGGCGAAAGGGGGA	16	#5_ends	F15
16[020]	16[005]	GAACGAACCACCAGCA	16	#5_ends	E16
16[361]	16[348]	AAGGGCGATCGGTG	14	#5_ends	F16
17[005]	17[020]	GAAGATAAAACAGAGG	16	#5_ends	E17
17[343]	17[361]	AGGCTGCGCAACTGTTGGG	19	#5_ends	F17
18[020]	18[005]	CTGCAACAGTGCCACG	16	#5_ends	E18
18[361]	18[343]	TCCGGCACCGCTTCTGGTG	19	#5_ends	F18
19[005]	19[020]	CTGAGAGCCAGCAGCA	16	#5_ends	E19
19[348]	19[361]	ACTCCAGCCAGCTT	14	#5_ends	F19

Supplementary Table 2

List of DNA staples - Origami Q

Start	*End*	*Sequence*	*Length*	*Type*	*Pos.*
00[031]	03[027]	ACAGGGCGCGTACTTTCCTCGAGCGGAGACAG	32	#0_core	
00[053]	06[049]	CCCGCCGCGCTTATTACAAACAATTTTGAGTAAC	34	#0_core	
00[073]	06[070]	CGCTGCGCGTACTGAGCAAAAGAGGATTTGAA	32	#0_core	
00[094]	06[091]	GCGCTGGCAAGTACCGACCGTGTATGCCTGTT	32	#0_core	
00[115]	06[112]	AGCGAAAGGAGCAGAACGGGTATTAAAGGAATC	33	#0_core	
00[136]	06[133]	TGGCGAGAAAGGAGACGGGAGAATTAAGATAACC	34	#0_core	
00[157]	06[154]	AACCGTCTATCATTCAACCGATTACACCGTCA	32	#0_core	
00[178]	06[175]	ACTCCAACGTCGAGCCGCCGCCACTAATAAAT	32	#0_core	
00[199]	06[196]	AGTCCACTATTATTTAGTACCGCCCGATTTTCA	33	#0_core	
00[220]	06[217]	GTTGTTCCAGTTTTCTTAAACAGCTGATATAT	32	#0_core	
00[241]	06[238]	ATAGCCCGAGATACTTAGCCGGAACAGGACAGAT	34	#0_core	
00[262]	06[259]	AAATCCCTTATAAATACCACATTCCCAACACTA	33	#0_core	
00[283]	06[280]	GATGGTGGTTCACTCCAACAGGTGTGGATGGC	32	#0_core	
00[304]	06[301]	CCAGCAGGCGAACATTATGACCCTAGAACCCT	32	#0_core	
00[325]	06[322]	AAGCGGTCCACGAAAATTCGCATTGGAATTCGC	33	#0_core	
00[346]	01[346]	CTGGCCCTGAGATTTCACCAGTGA	24	#0_core	
02[034]	07[048]	AGAATCAGTTAGTAGTTGTAGTATCGGCCTTGCGATTCATTTT	43	#0_core	
02[346]	02[329]	GCGTATTGGGCGCCAGG	17	#0_core	
03[028]	03[063]	GAACGGTACGCCTACATTTCGTATTAAATCCGCG	34	#0_core	
03[064]	03[105]	CAGAGAAACAAACATCAATCTTCTAAGGCGTTAAATAAATA	41	#0_core	
03[106]	03[147]	ATCGTACCGCACTCATCGAATAACACCCTGAACAAAGGTT	40	#0_core	
03[148]	03[189]	TACCGGAAGGTAAATATCCCTCAGCAGGAGGTTGAGGCGGA	41	#0_core	
03[190]	03[231]	ATAGAACCGCCACCCTATCGGTTCCGATAGTTGCGCCTGT	40	#0_core	
03[232]	03[273]	CGAAGACGGTCAATCATGTAGAAGCAGATACATAACGAG	39	#0_core	
03[274]	03[315]	CTTCAGAGAGTACCTTTGAGCATACTTTTGCGGGAGAAATT	41	#0_core	
03[316]	03[346]	TAATGTTAAATCAGCTCGCTGCATTAATG	29	#0_core	
04[048]	10[028]	GTTAATAAAGAATCCTGAGAGCAAATTCTACATT	34	#0_core	
04[090]	04[049]	GAATCTTTCAGAAAACAAAATTAATAATCTTTGCCCGAAC	40	#0_core	
04[132]	04[091]	TGAGGAGAGAGAACAAGCAAGCCGATCAGAATAAACACCG	40	#0_core	
04[174]	04[133]	TGGCCACCATGACGGAAATTATTCATATGTCAGAGGGTAAT	41	#0_core	
04[216]	04[175]	AACCATTGTCAGAACCGCCACCCTGCCCAGGTCAGACGAT	40	#0_core	
04[258]	04[217]	TTACACAGAAGGGAACCGAACTGAATTGGACAATGACAAC	40	#0_core	
04[300]	04[259]	ACGCACTCAAATTGCTCCTTTTGATATTCGCCAAAAGGAA	40	#0_core	
04[342]	04[301]	TCGTGCCAATTTTTTAACCAATAGAAATGCCTTTATTTCA	40	#0_core	
05[049]	05[048]	TTTAAAAAATATCATCTATCTAAAATATAATCAATAGTTAAT	42	#0_core	
05[070]	05[069]	TTAACAACAGAATAACATCGGGAGAAACGTGTTACAATACAT	42	#0_core	
05[091]	05[090]	TACTAGAAAACGCTCACCAATCGCAAGATTTAGTTAAATAAT	42	#0_core	
05[112]	05[111]	ATTTTCAGACCGGTATGTCCTGAACAAGAAGAAACCATTTTT	42	#0_core	
05[133]	05[132]	AATATCACAAATAGCAAAATAAGAAACTCCTTTACACGCT	40	#0_core	
05[154]	05[153]	GGTGAATTCGCACCATACGCAAAGACACGAAAATTCATTAAA	42	#0_core	
05[175]	05[174]	ATTCACAACGTTCCAGCCGGAACCGCCTAAGAGCCTTGAT	40	#0_core	
05[196]	05[195]	CCACCACTTTTCGTCAGTTTTGCTCAGTAAAAGTATACAGAG	42	#0_core	
05[217]	05[216]	CACGCATCGGATCGTCAATTTTTTCACGCCCCTTTAATCGCC	42	#0_core	
05[238]	05[237]	CTTTGAAAAGAGTAATATTATACCAAGAACTGATAACCAA	40	#0_core	
05[259]	05[258]	ATAGTAAAGAGGCTTTACGTTGGGAAGAAGCATTATTGAGGC	42	#0_core	
05[280]	05[279]	GTCATTTTAAAATATGTTGCATCAAAAGAGTTTTAAAGAG	40	#0_core	
05[301]	05[300]	AAAAATTTGTTCAAAATCCAATAAATCATTATAAAGCAGGAT	42	#0_core	
05[322]	05[321]	CCATCAATACGAGTAAATGTACCCCGGTTTTATAAGCGAACG	42	#0_core	
06[048]	00[032]	ATTATAGAGCCGCCTTAGACTATGCGCCGCT	31	#0_core	
06[069]	00[054]	TTACCATACCAATTCAATTACACCACCACA	30	#0_core	
06[090]	00[074]	TAGTATATATTTTCTTTGAAATGTAGCGGTCA	32	#0_core	
06[111]	00[095]	ATTACGCATGTAAACATTCCAGGGCGCTAGG	31	#0_core	
06[132]	00[116]	CACAAGTAGCAGCGTGCGCATTAAGGGAAGAA	32	#0_core	
06[153]	00[137]	CCGACCAATAGAGAGGGCGACACGGCGAACG	31	#0_core	
06[174]	00[158]	CCTCACCACCCTCTCCACCACCAAAAGGGCGAAA	34	#0_core	
06[195]	00[179]	GGGATTTGATATCACAGGAGGAAGAACGTGG	31	#0_core	
06[216]	00[200]	TCGGTAAAGGAGTCAGGTGAATTGGAACAAG	31	#0_core	

06[237]	00[221]	GAACGGTCATCGCCCCCATGTTAGGGTTGAGT	32	#0_core	
06[258]	00[242]	TCATAGGAACAAAGATTTAGGAATCAAAAGA	31	#0_core	
06[279]	00[263]	TTAGAAATATCGCGCGGAAGCAACGAAATCGGCA	34	#0_core	
06[300]	00[284]	CATATTTAAGCAGCACCAAAAAAATCCTGTTT	32	#0_core	
06[321]	00[305]	GTCTGAAGATTGTATTTTGTTCTGGTTTGCC	31	#0_core	
06[342]	00[326]	GCTAACTCACATTATTTTTTCTGAGTTGCAGC	32	#0_core	
07[021]	04[021]	ACTCAAACCAATACTTCATCACAGTGTT	28	#0_core	
07[049]	07[090]	GCGGAACAAAGATTGATTTTTTAATGGAAACAGTCAAATCATA	43	#0_core	
07[091]	07[132]	TGCGTTATACAAACGACGCGCCCAATAGCAAGCAAAAAAATTGA	44	#0_core	
07[133]	07[174]	GTTAAGCCCAATCAATTTGAGCCATTTGGGAATTACCGTTAAA	43	#0_core	
07[175]	07[216]	GCCAGAATGGAAAGGGAGCAAGCCCAATAGGAACTCCACGCTG	43	#0_core	
07[217]	07[258]	AGGCTTGCAGGGTGTATGTACAGACCAGGCGCATTAACACCCTC	44	#0_core	
07[259]	07[300]	GTTTACCAGACGATTCAGCTTAATTGCTGAATATAAAAAATTTT	44	#0_core	
07[301]	07[342]	AAATGCAATGCCCAGGGCCTTCCTGTAGCCAGCTCCGGAAGCAT	44	#0_core	
08[048]	08[021]	GAAACTAATATGGTAATATCCAGAACAATA	30	#0_core	
08[090]	08[070]	ATACTTTTTACATAAATCAATA	22	#0_core	
08[132]	08[112]	AACAAAAATGAATCAGATATAG	22	#0_core	
08[174]	08[153]	GACCTCAGAAGAGCCAGCAAAAT	23	#0_core	
08[216]	08[196]	CTAAAAGGCCCATGTACCGTA	21	#0_core	
08[258]	08[238]	AAAAACGAACAGGCTGGCTGAC	22	#0_core	
08[300]	08[280]	AGAATTAGCATGCTGTAGCTCA	22	#0_core	
08[347]	11[347]	CACACAACATACGATAGCTGTGAGCTCAGCTTGCATGC	38	#0_core	
09[021]	15[048]	CATGGAAATACCTTTCACAGACTCGCCATTAAAATCAATATACT	44	#0_core	
09[063]	09[062]	CGCCTTGTGAGTAACCTTGAAAACATAATATACCTACGGATT	42	#0_core	
09[105]	09[104]	CCCATGGCTTATTTTTTTGAAGCCTTCGCCTGTTGATAATAT	42	#0_core	
09[147]	09[146]	TTTATACCAGTACCCAGCACCGTAATATAAAGGAGGAATAAG	42	#0_core	
09[189]	09[188]	AAGTGACTGAGTGAATAGTTAGCGTAAAGAGAATAGGCGGAT	42	#0_core	
09[231]	09[230]	GTACAATCATCAAAACTGCTCATTCAGTAACACTCTAAAACAAA	44	#0_core	
09[273]	09[272]	AGCCCATGTTTTCAATTCCCAATTCTCTATTATAAGTAAGAGGA	44	#0_core	
09[315]	09[314]	AAAGCATGTGAGATTGGGATAGGTCATCGTAAATCAATCAGA	42	#0_core	
10[027]	11[048]	TTGACGCAGATTCACCAGTCACACGAAGGTAT	32	#0_core	
11[070]	11[090]	TGCTTCTGTAATGCAAATAC	20	#0_core	
11[112]	11[132]	TAAGAACGCGAGCAATCCAT	20	#0_core	
11[154]	11[175]	CCATTAGCAAGGCCACCATAA	21	#0_core	
11[196]	11[216]	AGTACAAACTAGAATAATAC	20	#0_core	
11[238]	11[258]	TGACAAGAACCGGTCAGGTG	20	#0_core	
11[280]	11[300]	ACTAAAGTACGATTAACAGG	20	#0_core	
11[322]	05[342]	ACCCGTCGGATTCAGAATTCGAAAATTGCGTTGC	34	#0_core	
12[048]	18[028]	CAATTGAGGACCAGTAATAAAGAATACATAACAG	34	#0_core	
12[090]	12[049]	ATATTCTGAATCGTCGCTATTAATTGTACTCAGATGATGG	40	#0_core	
12[132]	12[091]	AAGAATCCGCGTTTTAGCGAACCTCAATTTGAGAATCGCC	40	#0_core	
12[174]	12[133]	TACAGCAGAGCCGGAAACGTCACCAATATGAAGCCCTTTTT	41	#0_core	
12[216]	12[175]	GAACGTTGCCAACGCCTGTAGCATGATTGGCTTTTGATGA	40	#0_core	
12[258]	12[217]	GGTAACCAGATATTCATTACCCAAACCCAAGACAGCATCG	40	#0_core	
12[300]	12[259]	ATCACTAGCGTGTCTGGAAGTTTCAAGCATTGCCAGAGGG	40	#0_core	
12[335]	12[301]	GCCTCCGTGGGAACAAACTGTCGGAGACAGTCAA	34	#0_core	
13[049]	13[048]	AATATAAGTCAAAATTATCAAACCCTCAGTAAGGAATTCATC	42	#0_core	
13[091]	13[090]	ACGCCAAAACGACAAAGACTACCTTTTTGAGTAAATGTAACA	42	#0_core	
13[133]	13[132]	TAAGCAGATCCAAAAGAGCGTCTTTCCGAATTATTTAAAG	40	#0_core	
13[175]	13[174]	TACTGGTTGATGCCCCCATAGCCCCCTATCGGAACGAGTG	40	#0_core	
13[217]	13[216]	AGCAACGAAGGGTAAAACAACTTTCAACCCAAAGGAAAGGGT	42	#0_core	
13[259]	13[258]	AAAATGTTATCAAATGTCAACTTTAATCAATCATTATATAGT	42	#0_core	
13[301]	13[300]	ATATGATAGGATCTACATTTTCATTTGGGTATAGTAGCATCA	42	#0_core	
14[048]	14[049]	TGGATCAGTTGAGGAGGAGCACTAACGCGGAATTAGATTGTT	42	#0_core	
14[090]	14[091]	GGCAGACTATATATGAACGCGAGAAAAAAGCCAAATAATTTA	42	#0_core	
14[132]	14[133]	AAGTTACAGCCATGGTTTTGTTTAACGTACAATGACGCCGAACA	44	#0_core	
14[174]	14[175]	ACGGGAAAATCACAGTCAGAGCCGCCACATTTACCCAAGTTTTA	44	#0_core	
14[216]	14[217]	TTTGAAACAACTTCAAATCTCCAAAATTTGCGGCGCAGAGGC	42	#0_core	
14[258]	14[259]	AGCGTAACTGGCAGTCTACGTTAATATAGCGAGATACTGGAT	42	#0_core	
14[300]	14[301]	CTAGCTCTACTATGGGCAAGGCAAAGGTAAAGAATAACCGTT	42	#0_core	
14[342]	08[322]	GGCGATTAAGTTGAACATGGTCAGTTCATCAACATT	36	#0_core	
15[021]	12[021]	CTAAAACAAATATTTTAGCGTAAAGGGA	28	#0_core	
15[049]	15[090]	TCTGAATAATGGGATGAGCGATAGCTTAGATTAATATAAGGCA	43	#0_core	
15[091]	15[132]	TTTTCGAGCCAGAACGAAATCAAGATTAGTTGCTTAAACCAGAA	44	#0_core	

15[133]	15[174]	GGAAACCGAGGAATACCAGTAGCGACAGAATCAAAATCGTCAG	43	#0_core	
15[175]	15[216]	TGCCTTGAGTAACCTCACGATCTAAAGTTTTGTCAAGGGGACT	43	#0_core	
15[217]	15[258]	AAAGACTTTTTCCTAAGAATAAGGCTTGCCCTGATAAGCCAATA	44	#0_core	
15[259]	15[300]	CTGCGGAATCGTCCTGAGCGAACGAGTAGATTTAGCAATTGATA	44	#0_core	
15[301]	15[342]	AATTAATGCCGGGTAACGTTGGTGTAGATGGGCGCTTCGCTATT	44	#0_core	
16[062]	16[021]	CAAAGGGTTAGAACCTTTGGCAAAATACCGAACGAACCACCAGC	44	#0_core	
16[104]	16[063]	AGTAATAAGAGAATATTGGGTTAGACGCTGAGAAGAGTAACGT	43	#0_core	
16[147]	16[105]	TACAACGCAATAATAACTTACAAAAATTTTGCACCCAGCTAATGC	45	#0_core	
16[189]	16[148]	ACTCAGTGCCCGTATAATTTTCATGTTTGCCTTTAGCGTAAAA	43	#0_core	
16[231]	16[190]	ACAATGAGGAAGTTTCCGAATAGAGTCTTTCCAGACGTCTGAG	43	#0_core	
16[273]	16[232]	ACCATAAATATTCATTGATGCGATTTCGAGAAACACCAGAAGAAT	45	#0_core	
16[315]	16[274]	ACGAGAGGGTAGCTATTGTGGCATTTTGACCATTAGATACTTT	43	#0_core	
16[347]	19[347]	GATCGGTGCGGGCGCGCCATTTTCTGGTCGGCCTCAGG	38	#0_core	
17[021]	17[034]	TAACACCGCCTGCCA	15	#0_core	
17[329]	16[316]	AGGCAAACTCATCGTAACCGTGCGATGA	28	#0_core	
18[027]	19[062]	TGCCACGCATCACCTTGCTGAACCTCAAATATTTGCACGTAAAAC	45	#0_core	
18[062]	18[063]	AAATTGCTCAGTAACAAATTTTCCCTTAAAGAATTTATAAAG	42	#0_core	
18[104]	18[105]	CGACAATCATTATCAACCCGACTTGCGGAGTTATCCTGACGA	42	#0_core	
18[146]	18[147]	ACGCAGTATTGGCAACATGAAACCATCGTATAGCGCGTTATT	42	#0_core	
18[188]	18[189]	TGAAACAAAGGATTAGTCCACAGACAGCAGTGAATTTTATTC	42	#0_core	
18[230]	18[231]	CCAACCTGCATCTTTGATCAACGTAACATGTAAATTGGCA	40	#0_core	
18[272]	18[273]	TGACCATTGTCAGATTCCATATAACAGGCAAATGGGAGAA	40	#0_core	
18[314]	18[315]	AGTCTGGATACTAGCAGGCGGATTGACCAAAGTTTGACTGAG	42	#0_core	
19[063]	19[104]	AGAAATCAAAATCATAGGTCTGAGAAGGTAAAGTAATTCTG	41	#0_core	
19[105]	19[146]	TCCAGAATCTTACCAACGCTAACGAACTGGCATGATTAAG	40	#0_core	
19[147]	19[188]	ACTCCTTTTCATCGGCATTTTCGGTCTGCCTATTTCGGAAC	41	#0_core	
19[189]	19[230]	CTATTCTGTATGGGATTTTGCTAAATACGTAATGCCACTA	40	#0_core	
19[231]	19[272]	CGAAGGGCTTGAGATGGTTTAATTCTTTAAACAGTTCAG	39	#0_core	
19[273]	19[314]	AAAACTCAATAACCTGTTTAGCTATAAAGGCTATCAGGTCA	41	#0_core	
19[315]	12[336]	TTGCGGGGACGACGACAGTATGCCGGAGTGGTAACGCCAGGCCAGT	46	#0_core	
11[049]	11[069]	ATTCCTGATTACTTTTACCT	20	#1_bottom	B0
11[091]	11[111]	AGTAGGGCTTAAAGATAATC	20	#1_bottom	B1
11[133]	11[153]	AGCTATCTTACCAAAAGAATA	21	#1_bottom	B2
11[176]	11[195]	GCGTCATACATAGCGGGCC	19	#1_bottom	B3
11[217]	11[237]	CCTCAGCAGCGACCAGCGCT	20	#1_bottom	B4
11[259]	11[279]	CAAAAGAAGTTAAGCGGACA	20	#1_bottom	B5
11[301]	11[321]	GTGAGAAAGGCCAATCATCA	20	#1_bottom	B6
08[069]	08[049]	TAGATTGCTAACCACCAGAAG	21	#2_top	T0
08[111]	08[091]	AACCTAATTTATTCTTACCAGT	22	#2_top	T1
08[152]	08[133]	CTTTGTCAAATAAGAGCAAG	20	#2_top	T2
08[195]	08[175]	ACCCGTCGAGAGCGCAGTCTCT	22	#2_top	T3
08[237]	08[217]	CTCGGAGATTAGTTAAAGGCCG	22	#2_top	T4
08[279]	08[259]	ACGAAAGACCGATAAAAACCAA	22	#2_top	T5
08[321]	08[301]	AACCCAAAAATGAGTAATGTGT	22	#2_top	T6
02[055]	02[035]	ATCGACAACTGAGGATTTAGA	21	#3_left	L0
02[076]	02[056]	ATAGATGATGGCGAATTATTC	21	#3_left	L1
02[097]	02[077]	TTGATAAATGACCTAAATTTA	21	#3_left	L2
02[118]	02[098]	GGAAACCAAGGCTGTCTTTCC	21	#3_left	L3
02[139]	02[119]	AAAACTGAACATAAAAACAG	20	#3_left	L4
02[160]	02[140]	GAGAGGGAGAGCGCCAAAGAC	21	#3_left	L5
02[181]	02[161]	TAGCATTGAAGCCGCCACCA	20	#3_left	L6
02[202]	02[182]	TTACCCTCAGGTGTATCACCG	21	#3_left	L7
02[223]	02[203]	TGCTTGATATATCAGCTTGCT	21	#3_left	L8
02[244]	02[224]	TGGAGGCGCAATCCGCGACC	20	#3_left	L9
02[265]	02[245]	GAAACTAATAGATTCATCAGT	21	#3_left	L10
02[286]	02[266]	TTCAGGATTAAAGCGAACCA	20	#3_left	L11
02[307]	02[287]	AATGTAATAAAGCTAAATCGG	21	#3_left	L12
02[328]	02[308]	GTAAATTTTATTGTAAACGTT	21	#3_left	L13
17[035]	17[055]	ATATCTGGTCAGACCATATAG	21	#4_right	R0
17[056]	17[076]	ATTTTCAGGTTTCAATAGTCC	21	#4_right	R1
17[077]	17[097]	TCCGGCTTAGGTAAAGTACAC	21	#4_right	R2
17[098]	17[118]	AACATGTTCAGCTACAATTAG	21	#4_right	R3
17[119]	17[139]	CCTAATTTGCCAGGGAATACGT	22	#4_right	R4
17[140]	17[160]	TAGCAAACGTAGCAGACTGTT	21	#4_right	R5

17[161]	17[181]	AGCGTTTGCCATCACAGTTAAA	22	#4_right	R6
17[182]	17[202]	AGTATTAAGAGGTAGTAAATT	21	#4_right	R7
17[203]	17[223]	TCAGCGGAGTGAATTAAACAA	21	#4_right	R8
17[224]	17[244]	CGAAAGAGGCAAAACGAGTATG	22	#4_right	R9
17[245]	17[265]	TGAATTACCTTAATCCCCCAA	21	#4_right	R10
17[266]	17[286]	TCAAAAATCAGGTCATTTCGCG	22	#4_right	R11
17[287]	17[307]	CGAGCTGAAAAGTTTGAGACA	21	#4_right	R12
17[308]	17[328]	AACAAGAGAATCATCTGCCCC	21	#4_right	R13
00[020]	00[005]	CTATGGTTGCTTTGAC	16	#5_ends	E0
00[361]	00[347]	ATTGCCCTTCACCGC	15	#5_ends	F0
01[005]	01[020]	GAGCACGTATAACGTG	16	#5_ends	E1
01[347]	01[361]	GACGGGCAACAGCTG	15	#5_ends	F1
02[020]	02[005]	GAGCTAAACAGGAGGC	16	#5_ends	E2
02[361]	02[347]	GGGGAGAGGCGGTTT	15	#5_ends	F2
03[005]	03[020]	CGATTAAAGGGATTTT	16	#5_ends	E3
03[347]	03[361]	AATCGGCCAACGCGC	15	#5_ends	F3
04[020]	04[002]	TTTATAATCAGTGAGGCCA	19	#5_ends	E4
04[358]	04[343]	CCAGTCGGGAAACCTG	16	#5_ends	F4
05[002]	05[020]	CCGAGTAAAAGAGTCTGTC	19	#5_ends	E5
05[343]	05[358]	GCTCACTGCCCGCTTT	16	#5_ends	F5
06[020]	06[002]	CTTTGATTAGTAATAACAT	19	#5_ends	E6
06[358]	06[343]	GGTGCCTAATGAGTGA	16	#5_ends	F6
07[002]	07[020]	CACTTGCCTGAGTAGAAGA	19	#5_ends	E7
07[343]	07[358]	AAAGTGTAAAGCCTGG	16	#5_ends	F7
08[020]	08[005]	TTACCGCCAGCCATTG	16	#5_ends	E8
08[361]	08[348]	TCCGCTCACAATTC	14	#5_ends	F8
09[005]	09[020]	CAACAGGAAAAACGCT	16	#5_ends	E9
09[343]	09[361]	TTCCTGTGTGAAATTGTTA	19	#5_ends	F9
10[020]	10[005]	CTCAATCGTCTGAAAT	16	#5_ends	E10
10[361]	10[343]	TAGAGGATCCCCGGGTACC	19	#5_ends	F10
11[005]	11[020]	GGATTATTTACATTGG	16	#5_ends	E11
11[348]	11[361]	CTGCAGGTCGACTC	14	#5_ends	F11
12[020]	12[002]	CATTCTGGCCAACAGAGAT	19	#5_ends	E12
12[358]	12[343]	ACGTTGTAAAACGACG	16	#5_ends	F12
13[002]	13[020]	AGAACCCTTCTGACCTGAA	19	#5_ends	E13
13[343]	13[358]	GGTTTTCCCAGTCACG	16	#5_ends	F13
14[020]	14[002]	TGAATGGCTATTAGTCTTT	19	#5_ends	E14
14[358]	14[343]	GGGGGATGTGCTGCAA	16	#5_ends	F14
15[002]	15[020]	AATGCGCGAACTGATAGCC	19	#5_ends	E15
15[343]	15[358]	ACGCCAGCTGGCGAAA	16	#5_ends	F15
16[020]	16[005]	AGAAGATAAAACAGAG	16	#5_ends	E16
16[361]	16[348]	CTGTTGGGAAGGGC	14	#5_ends	F16
17[005]	17[020]	GTGAGGCGGTCAGTAT	16	#5_ends	E17
17[343]	17[361]	CGCCATTCAGGCTGCGCAA	19	#5_ends	F17
18[020]	18[005]	GCTGAGAGCCAGCAGC	16	#5_ends	E18
18[361]	18[343]	GCCAGCTTTCCGGCACCGC	19	#5_ends	F18
19[005]	19[020]	AAATGAAAAATCTAAA	16	#5_ends	E19
19[348]	19[361]	AAGATCGCACTCCA	14	#5_ends	F19

Supplementary Table 3
List of DNA staples - Origami H

Start	*End*	*Sequence*	*Length*	*Type*	*Pos.*
00[031]	03[027]	GTACTATGGTTGTTAGAATCCTAAACCGGTAC	32	#0_core	
00[053]	06[049]	GCTTAATGCGCCGAATTCGACAACTTATTATCAT	34	#0_core	
00[073]	06[070]	CGTAACCACCACAAAGAAGATGATAAATTACCT	33	#0_core	
00[094]	06[091]	CAAGTGTAGCGCGTGTGATAAATACAGTTTAGTA	34	#0_core	
00[115]	06[112]	AGGAGCGGGCGCGTATTAAACCAAATTCATTAC	33	#0_core	
00[136]	06[133]	AGAAAGGAAGGGGAGAATTAACTGACGACCCACA	34	#0_core	
00[157]	06[154]	AACCGTCTATCACCGATTGAGGGCACACCGAC	32	#0_core	
00[178]	06[175]	ACTCCAACGTCGCCGCCGCCAGCACAAATAAA	32	#0_core	
00[199]	06[196]	GAGTCCACTATTGTTTAGTACCGCCGGTCATTTTC	35	#0_core	
00[220]	06[217]	GTGTTGTTCCACTTAAACAGCTTCCATATATT	32	#0_core	
00[241]	06[238]	AGAATAGCCCGATTAGCCGGAACGAGAGACAGAT	34	#0_core	
00[262]	06[259]	GGCAAAATCCCTATACCACATTCACGACACTAT	33	#0_core	
00[283]	06[280]	GTTTGATGGTGCTCCAACAGGTCTTGATGGCT	32	#0_core	
00[304]	06[301]	TGCCCCAGCAGAAACATTATGACCGTTTTTTAGA	34	#0_core	
00[325]	06[322]	GCAGCAAGCGGTTTGTTAAAATTCCCCAAAAAT	33	#0_core	
00[346]	01[346]	CACCGCCTGGCCTTTTTCTTTTCA	24	#0_core	
02[034]	07[048]	ACGGGAGAGAGCTTCAATACTCTTGCTGGTAATACCGTGGAACA	44	#0_core	
02[346]	02[329]	GCGGTTTGCGTATTGGG	17	#0_core	
03[028]	03[063]	GCCAGAATCCTAGGATTAATCCTTTGCCCGGCG	33	#0_core	
03[064]	03[105]	AATACATCAAGAAAACGACCTAATAAATAAGAATAAGCT	39	#0_core	
03[106]	03[147]	GTCACTCATCGAGAACCATAAAGAACAAAGTCAGACCA	38	#0_core	
03[148]	03[189]	GCGCGTAAATATTGACGTCAGAGGGAGGTTGAGGCACGG	39	#0_core	
03[190]	03[231]	AATAGAACCGCCACCCGGTTTATATAGTTGCGCCGATCG	39	#0_core	
03[232]	03[273]	AAAACGGTCAATCATATAGAAACAGATACATAACGAGC	38	#0_core	
03[274]	03[315]	TTCAGAGAGTACCTTTAAGAGCATACTTTTGCGGGACAA	39	#0_core	
03[316]	03[346]	ATAATTTTTGTTAAATGTGCCAGCTGCA	28	#0_core	
04[048]	10[028]	TTTTTTTGGAGAAGTGTTTAACCGTTGCCGCTC	33	#0_core	
04[090]	04[049]	ATAATTTCTAAAATTAATTACATTAGAGAACGTTATTAA	39	#0_core	
04[132]	04[091]	CGCTATAAAAGCAAGCCGTTTTTATCGACACCGGAATC	38	#0_core	
04[174]	04[133]	TGGCACCCGAAATTATTCATTAAATTTAGGGTAATTGAG	39	#0_core	
04[216]	04[175]	ACCATTATCTCAGAACCGCCACCCAGCCGGTCAGACGAT	39	#0_core	
04[258]	04[217]	TACGCAGGAGGGAACCGAACTGATGTGCAATGACAACA	38	#0_core	
04[300]	04[259]	TTCACCTCATTGCTCCTTTTGATATTCGCCAAAAGGAAT	39	#0_core	
04[342]	04[301]	AACCTGTCCAGCTCATTTTTTAATAAGGAAGCCTTTAT	38	#0_core	
05[049]	05[048]	TTTGAGTGGTATTCCTATATCTTTAGGAGTTAATACAAAAAG	42	#0_core	
05[070]	05[069]	ATTTCATCGCTTGCTTGAAACAATAACAATCGCGCTAACA	40	#0_core	
05[091]	05[090]	GAAAAAATCAGTAGCAAGACAAAGAATTTTTCATCTACTA	40	#0_core	
05[112]	05[111]	CATCGTATTTCTAAGAACAAGAAAAATAGCATCAATAATTTT	42	#0_core	
05[133]	05[132]	TCAGAGAAAAATAGCTGAAACGATTTTGGAGAGAGAAATA	40	#0_core	
05[154]	05[153]	ATTATCACTATTACCAAAGACACCACGGGACATATGGGGTGA	42	#0_core	
05[175]	05[174]	TATTCACTATTCCAGTGAACCGCCTCCCCGCCACCCTTGA	40	#0_core	
05[196]	05[195]	GCCACCAACGTCACGGTTTTGCTCAGAATAAGTATTCAGA	40	#0_core	
05[217]	05[216]	ACGCATACGCGTCACCTTTTTCACGTTGACTTAATTGCGCCC	42	#0_core	
05[238]	05[237]	CTTTGAAAAGTAATCTTATACCAAGCGACGATAAATCCAA	40	#0_core	
05[259]	05[258]	TAGTAAGGAGGCTTTTCGTTGGGAAGAAAGATTATTAAGGCA	42	#0_core	
05[280]	05[279]	TCATTTTATAATATGCTGCATCAAAAAAGTTTTAAAGAGG	40	#0_core	
05[301]	05[300]	AGGATACCGATTCAATCCAATAAATCTGAATAAAGACGCA	40	#0_core	
05[322]	05[321]	AGGAACGGAGTGAGCGATCATATGTACCAAGATTGTACCAAT	42	#0_core	
06[048]	00[032]	TTTGCCAATAGAAGTACAAACCTACAGGGCGC	32	#0_core	
06[069]	00[054]	TTTTTTTACAAAACACCTGAGCAACCCGCCGC	32	#0_core	
06[090]	00[074]	TCATATAGTTAAGATACCGACGTCACGCTGCG	32	#0_core	
06[111]	00[095]	CGCGCGAAACCACTAGAACGGTAGGGCGCTGG	32	#0_core	
06[132]	00[116]	AGAATTCCTTTACAATAGACGGAAGAAAGCGAA	33	#0_core	
06[153]	00[137]	TTGAGGAAAATTTAACATTCAAGAACGTGGCG	32	#0_core	
06[174]	00[158]	TCCTCCCCTCAGAGTCCACCAGAAAAGGGCGAAA	34	#0_core	
06[195]	00[179]	AGGGAGTTGATAACTCAGGAGAAAGAACGTGG	32	#0_core	
06[216]	00[200]	CGGTCGGAGCCTCCTGAATTTGTTTGGAACAA	32	#0_core	

06[237]	00[221]	GAACGGATCGCCTCGATGTTACGATAGGGTTGA	33	#0_core	
06[258]	00[242]	CATAAGAACAACAGTTTAGGATATAAATCAAA	32	#0_core	
06[279]	00[263]	TAGAGATATCGCGCAGAAGCAAAGTTCCGAAATC	34	#0_core	
06[300]	00[284]	ACCCTATTAAGCCGGTACCAAGCGAAAATCCT	32	#0_core	
06[321]	00[305]	AATTCACAGGAAAATAATATTCCACGCTGGTT	32	#0_core	
06[342]	00[326]	AGTGAGCTAACTCATAGGGTGGCTGAGAGAGTT	33	#0_core	
07[021]	04[021]	CTATCGGCTCTTTGATCAAATTTTATAA	28	#0_core	
07[049]	07[090]	AAGAAACCACCACAAGAATGGAAACAGTACATAAAATTTTGCGT	44	#0_core	
07[091]	07[132]	TATACAAATTCTTTGTACCAATAGCAAGCAAATCAGGCAGGAGTTA	46	#0_core	
07[133]	07[174]	AGCCCAATAATAAAATACCATTTGGGAATTAGAGCGCCAATTAA	44	#0_core	
07[175]	07[216]	AGCCAGAATGGAAGAGGTAGCAAGCCCAATAGGAACAAAAGCTGA	45	#0_core	
07[217]	07[258]	GGCTTGCAGGGAGTATCTGTACAGACCAGGCGCATAAACGCCCTCG	46	#0_core	
07[259]	07[300]	TTTACCAGACGACTCAACTTAATTGCTGAATATAACAAACATAT	44	#0_core	
07[301]	07[342]	ATTTTAAATGCAAAAAAGCGTCTGGCCTTCCTGTAGTACGAGCCGG	46	#0_core	
08[048]	08[021]	TTGATTAGAGTCCAGAACAATATTACCGCCA	31	#0_core	
08[090]	08[070]	CACAAATATTCAATATATGTGA	22	#0_core	
08[132]	08[112]	TGTGAAAATAATATAGAAGGCTT	23	#0_core	
08[174]	08[153]	AACAGAACCCAGCAAAATCACCA	23	#0_core	
08[216]	08[196]	TTAGGCTCCCCATGTACCGTAA	22	#0_core	
08[258]	08[238]	ATAACGAACTGGCTGGCTGACCT	23	#0_core	
08[300]	08[280]	TGGAATTAGTGCTGTAGCTCAA	22	#0_core	
08[347]	11[347]	CAATTCCACACAACATGGTCAGGGTACGTGCCAAGCTT	38	#0_core	
09[021]	15[048]	ACCTACATTTTGAACAATATTTTAAAAATACCGAATGAATGAATA	45	#0_core	
09[063]	09[062]	TTGCTGAATAACCGAAACATAGCGATGTAACAGTTTTCGCCTGA	44	#0_core	
09[105]	09[104]	TAATTCCGGTATGTAAGCCTTAAATCTTATCAAATCCCATCC	42	#0_core	
09[147]	09[146]	TTTGTTAGCACCCACCGTAATCAGTAGGTGGCAGAAGTTTAT	42	#0_core	
09[189]	09[188]	TAAGTCTGAGTTTAAATAGTTAGCGTAACAAGAGAAAAGGCGGA	44	#0_core	
09[231]	09[230]	ACAACGATCAAGAATCTGCTCATTCAGTCACTCATACACAAAGT	44	#0_core	
09[273]	09[272]	GCCCGTGTTTTAACTTCCCAATTCTGTATTATAGGAAAGAGGAA	44	#0_core	
09[315]	09[314]	TCAGAATTAAATATACCGTAATGGGAGGTAATCATTTGATAA	42	#0_core	
10[027]	11[048]	AATCGTCAGTCACACGACCAGTATCTAAAGA	31	#0_core	
11[070]	11[090]	GTAAATCGTCGCCAATCGGG	20	#0_core	
11[112]	11[132]	GCGAGGCGTTTAAATAAAT	19	#0_core	
11[154]	11[175]	AGCAAGGCCGGCCACCGAAG	20	#0_core	
11[196]	11[216]	GTACAAACTACTAATAATCT	20	#0_core	
11[238]	11[258]	ACAAGAACCGGTCAGGAGC	19	#0_core	
11[280]	11[300]	CTAAAGTACGGATTAACAA	19	#0_core	
11[322]	05[342]	TAACAACCCGTCACGAGCTCCCACATTAATTGC	33	#0_core	
12[048]	18[028]	CAATGTTAATAAAAGGGACGTGGCACCTACGCT	33	#0_core	
12[090]	12[049]	TAACAAAATCTATTAATTAATTTTTACATGGCAATTCAT	39	#0_core	
12[132]	12[091]	AAAGATCCTAGCGAACCTCCCGATAAGATCGCCATATT	38	#0_core	
12[174]	12[133]	ATACGCCAAAACGTCACCAATGAAAAAGCCTTTTTAAGA	39	#0_core	
12[216]	12[175]	AACGACGAAAACGCCTGTAGCATTGGATGGCTTTTGATG	39	#0_core	
12[258]	12[217]	GTAACCAGATATTCATTACCCAACCCCGACAGCATCGG	38	#0_core	
12[300]	12[259]	GTCATAGCTGTCTGGAAGTTTCATGCAATGCCAGAGGGG	39	#0_core	
12[335]	12[301]	GCCGGATTCTCCGTGGGTAGCGGCCGGAGACA	32	#0_core	
13[049]	13[048]	CCTGATTTTTTTGCACCTCAATCAATATAGGAGGAAGATAAT	42	#0_core	
13[091]	13[090]	AACATGATGTAAAGCTTTTTAACCTCTTCTGATGCACGCC	40	#0_core	
13[133]	13[132]	CAGATAGAGGAACTGGTTTCCAGAGCCTTTATCCCATAAG	40	#0_core	
13[175]	13[174]	TGTACTGATATGCCCCGCCCCCTTATTAGACCAGAAGGAG	40	#0_core	
13[217]	13[216]	GCAACGGAATAAAATAACTTTCAACAGTCCGGAATTGGGGTA	42	#0_core	
13[259]	13[258]	AAATGTTAACAAATGCCAACTTTAATCAAACATTATATAGTA	42	#0_core	
13[301]	13[300]	CCATCATCAGAGATATTTTCATTTGGTTATAGTAGAATCA	40	#0_core	
14[048]	14[049]	ACTTCAGGAATTACTAACAACTAATAATCATCAATGGATTAT	42	#0_core	
14[090]	14[091]	AGGCAGTAAATGGAAGAAAACTTTTTACGCTCAAATTTAGGCAG	44	#0_core	
14[132]	14[133]	TACCAGTATTATTTGTTAACGTCAAAAAAAATAGCTAACAAAGT	44	#0_core	
14[174]	14[175]	TAACGTCACCGGACAGAGCCGCCACCCTTTTACCGCCTAAGTTT	44	#0_core	
14[216]	14[217]	TTGAGAACTAAATCTCTCCAAAAAAAGCGGGATAAAGAGGCT	42	#0_core	
14[258]	14[259]	GCGTCACTGGCTAGCTACGTTAATAAAGCGAGATTCTGGATA	42	#0_core	
14[300]	14[301]	ACCGTTCTACTAGAAGGCAAGGCAAATAGGTAAAGGGATATTCA	44	#0_core	
14[342]	08[322]	TGCAAGGCGATTATGTCGTAATCACCAGCTTTCATCA	37	#0_core	
15[021]	12[021]	ATCGCCATTTGAATGGGAATACATTCTG	28	#0_core	
15[049]	15[090]	ATGGAAGGGTTATACAAGCTTAGATTAAGACGCTGATATTTTTCG	45	#0_core	
15[091]	15[132]	AGCCAGTAATAAGCTGTAAGATTAGTTGCTATTTTGGCCAAAGGAA	46	#0_core	

15[133]	15[174]	ACCGAGGAAACGCTAAAGCGACAGAATCAAGTTTGAAAAGGGTC	44	#0_core	
15[175]	15[216]	AGTGCCTTGAGTATCCTCGATCTAAAGTTTTGTCGTGAACGACTA	45	#0_core	
15[217]	15[258]	AAGACTTTTTCATAAAAGAATAAGGCTTGCCCTGACAAGACAATAC	46	#0_core	
15[259]	15[300]	TGCGGAATCGTCATGACCGAACGAGTAGATTTAGTCAATTCTAG	44	#0_core	
15[301]	15[342]	CTGATAAATTAATGAACTAGGTCACGTTGGTGTAGAGGGCCTCTTC	46	#0_core	
16[062]	16[021]	TAGAACCTACCATATCTCAACAGTCGAACCACCAGCAGAAGATAA	45	#0_core	
16[104]	16[063]	GCAGAATATAAAGTACCATATAACTAGAAGAGTCAATAGTGATGAA	46	#0_core	
16[147]	16[105]	ACAAATAATAACGGAATAAATAAACACACCCAGCTACAATTGAACGC	47	#0_core	
16[189]	16[148]	GACACAGTGCCCGTATAACATAATCCCTTTAGCGTCAGACACAT	44	#0_core	
16[231]	16[190]	ACTGAGGAAGTTTCCATTTAGAAAGCTTTCCAGACGTTAGGCTGA	45	#0_core	
16[273]	16[232]	CCCTAAATATTCATTGAAGCGATTTTGAGAAACACCAGAACAATAC	46	#0_core	
16[315]	16[274]	GATGCCGGAGAGGGTAGCGTGGCATTTGACCATTAGATACTTTA	44	#0_core	
16[347]	19[347]	AAGGGCGATCGGTAGGCAAAGGCACCGACAGTATCGGC	38	#0_core	
17[021]	17[034]	CTGCAACAGTGCCGG	15	#0_core	
17[329]	16[316]	GGAAACCGCTGGGCGCATCGTAACGAATC	29	#0_core	
18[027]	19[062]	GAGAGCTGCTGAACCTCAAATATCAAACCGTAAAACAGAAATAA	44	#0_core	
18[062]	18[063]	GTAGATGTACCTTTCCCTTAGAATCCCGTCAAAATATTGC	40	#0_core	
18[104]	18[105]	AAACAACTACAATAGACTTGCGGGAGGTTAGAATCTTACAAT	42	#0_core	
18[146]	18[147]	GTATGTTCCACATATAACCATCGATAGCAGCGTTTTCACGCA	42	#0_core	
18[188]	18[189]	CTGAAACGTAGGATTACCACAGACAGCTTTTTTCTTTATT	40	#0_core	
18[230]	18[231]	AACCTAACTCTTTGACATCAACGTAACTTAAATTGGCACC	40	#0_core	
18[272]	18[273]	GACCATTATCAGAATCCATATAACAGGGAAATGGTAGAAT	40	#0_core	
18[314]	18[315]	CTGAGAGATGTAAAACAACAAACGGCGGTGCTGCCAGATTGC	42	#0_core	
19[063]	19[104]	AGAACATAGGTCTGAGAGACTACTAATTCTGTCCAGAC	38	#0_core	
19[105]	19[146]	GACGACCAACGCTAACGAGCGTCCATGATTAAGACTCC	38	#0_core	
19[147]	19[188]	TTATTATCGGCATTTTCGGTCATACTGCCTATTTCGGAA	39	#0_core	
19[189]	19[230]	CCTAGTATGGGATTTTGCTAAACACGTAATGCCACTACG	39	#0_core	
19[231]	19[272]	AAGGGCTTGAGATGGTTTAATTTTTTAAACAGTTCAGA	38	#0_core	
19[273]	19[314]	AAACGCAATAACCTGTTTAGCTATCTACAAAGGCTATCA	39	#0_core	
19[315]	12[336]	GGTCTTTGAGGGGACGACGCTTCTGGATAGTTGGGTAACACGACG	45	#0_core	
11[049]	11[069]	TTATCAGATGATCGGGACT	19	#1_bottom	B0
11[091]	11[111]	CTTAATTGAGATCCTGAAC	19	#1_bottom	B1
11[133]	11[153]	CTTACCGAAGCAAACGCATT	20	#1_bottom	B2
11[176]	11[195]	CGTCATACATTAGCGGCA	18	#1_bottom	B3
11[217]	11[237]	CAGCAGCGAAAAGCGATTG	19	#1_bottom	B4
11[259]	11[279]	AAAAGAAGTTTAGCGGATAA	20	#1_bottom	B5
11[301]	11[321]	AGGGTGAGAAAATGTCAAG	19	#1_bottom	B6
08[069]	08[049]	GTTTGAATACGAAGGAGCGGAA	22	#2_top	T0
08[111]	08[091]	ATTACGAGCAACCAGTATAAAGC	23	#2_top	T1
08[152]	08[133]	GCACAATCGAGCAAGAAACAA	21	#2_top	T2
08[195]	08[175]	CAGCCGTCGAAGCGCAGTCTCTG	23	#2_top	T3
08[237]	08[217]	TCGAGATTTGTTAAAGGCCGCTT	23	#2_top	T4
08[279]	08[259]	CAAAAGACTGATAAAAACCAAA	22	#2_top	T5
08[321]	08[301]	ACAAAGCCCCTGCCTGAGTAATG	23	#2_top	T6
02[055]	02[035]	ATCGTATTATAGAAGTATTAG	21	#3_left	L0
02[076]	02[056]	GAGAAACAATATTCATTTCA	20	#3_left	L1
02[097]	02[077]	TCAGGCGTATTTAATGGTTT	20	#3_left	L2
02[118]	02[098]	GCGTACCGCTTTCCTTATCAT	21	#3_left	L3
02[139]	02[119]	GGACACCCTAACAGGGAAGC	20	#3_left	L4
02[160]	02[140]	ACAGGGAAGCAAAGACAAAAG	21	#3_left	L5
02[181]	02[161]	GTATTGACACCGCCACCAGA	20	#3_left	L6
02[202]	02[182]	GAACCCTCAGGTGTATCACC	20	#3_left	L7
02[223]	02[203]	CTGATACCGCAGCTTGCTTTC	21	#3_left	L8
02[244]	02[224]	GAGGCGCAGTCCGCGACCTG	20	#3_left	L9
02[265]	02[245]	ACACTAATGGATTCATCAGTT	21	#3_left	L10
02[286]	02[266]	GGAGGATTAAAGCGAACCAG	20	#3_left	L11
02[307]	02[287]	ACCTGTAATAAAGCTAAATC	20	#3_left	L12
02[328]	02[308]	CGGCATTAATTTAAATTGTAA	21	#3_left	L13
17[035]	17[055]	TCAGTTGGCAAAAAAATTATC	21	#4_right	R0
17[056]	17[076]	AGGTTTAACGTCAGAATTTAGC	22	#4_right	R1
17[077]	17[097]	TTAGGTTGGGTTGACAAAAGGT	22	#4_right	R2
17[098]	17[118]	TCAGCTAATGCATTATCCTAT	21	#4_right	R3
17[119]	17[139]	TTGCCAGTTACAACCCAAAACA	22	#4_right	R4
17[140]	17[160]	AACGTAGAAAATTGTAGCGCG	21	#4_right	R5

17[161]	17[181]	TTTGCCATCTTTTACAGTTAGA	22	#4_right	R6
17[182]	17[202]	AAGTATTAAGAGTAAATGAACA	22	#4_right	R7
17[203]	17[223]	GCGGAGTGAGAAAAACGGGCG	21	#4_right	R8
17[224]	17[244]	AAAGAGGCAAAAGGAGTAGTGT	22	#4_right	R9
17[245]	17[265]	GAATTACCTTATTCCCCCTAT	21	#4_right	R10
17[266]	17[286]	CAAAAATCAGGTCATTTCGCCG	22	#4_right	R11
17[287]	17[307]	CGAGCTGAAAAGTATTTTTGTG	22	#4_right	R12
17[308]	17[328]	GAGCAAACAAGACGTGCATCC	21	#4_right	R13
00[020]	00[005]	CTTTGACGAGCACGTA	16	#5_ends	E0
00[361]	00[347]	ACAGCTGATTGCCCTT	16	#5_ends	F0
01[005]	01[020]	TAACGTGCTTTCCTCG	16	#5_ends	E1
01[347]	01[361]	CCAGTGAGACGGGCA	15	#5_ends	F1
02[020]	02[005]	AGGAGGCCGATTAAAG	16	#5_ends	E2
02[361]	02[347]	AACGCGCGGGGAGAG	15	#5_ends	F2
03[005]	03[020]	GGATTTTAGACAGGAA	16	#5_ends	E3
03[347]	03[361]	TTAATGAATCGGCC	14	#5_ends	F3
04[020]	04[002]	TCAGTGAGGCCACCGAGTA	19	#5_ends	E4
04[358]	04[343]	GCTTTCCAGTCGGGA	15	#5_ends	F4
05[002]	05[020]	AAAGAGTCTGTCCATCACG	19	#5_ends	E5
05[343]	05[358]	GTTGCGCTCACTGCCC	16	#5_ends	F5
06[020]	06[002]	TAGTAATAACATCACTTGC	19	#5_ends	E6
06[358]	06[343]	CCTGGGGTGCCTAATG	16	#5_ends	F6
07[002]	07[020]	CTGAGTAGAAGAACTCAAA	19	#5_ends	E7
07[343]	07[358]	AAGCATAAAGTGTAAAG	17	#5_ends	F7
08[020]	08[005]	GCCATTGCAACAGGAA	16	#5_ends	E8
08[361]	08[348]	ATTGTTATCCGCTCA	15	#5_ends	F8
09[005]	09[020]	AAACGCTCATGGAAAT	16	#5_ends	E9
09[343]	09[361]	TAGCTGTTTCCTGTGTGAA	19	#5_ends	F9
10[020]	10[005]	CTGAAATGGATTATTT	16	#5_ends	E10
10[361]	10[343]	TCGACTCTAGAGGATCCCC	19	#5_ends	F10
11[005]	11[020]	ACATTGGCAGATTCAC	16	#5_ends	E11
11[348]	11[361]	GCATGCCTGCAGG	13	#5_ends	F11
12[020]	12[002]	GCCAACAGAGATAGAACCC	19	#5_ends	E12
12[358]	12[343]	TCACGACGTTGTAAA	15	#5_ends	F12
13[002]	13[020]	TTCTGACCTGAAAGCGTAA	19	#5_ends	E13
13[343]	13[358]	GCCAGGGTTTTCCCAG	16	#5_ends	F13
14[020]	14[002]	CTATTAGTCTTTAATGCGC	19	#5_ends	E14
14[358]	14[343]	CGAAAGGGGGATGTGC	16	#5_ends	F14
15[002]	15[020]	GAACTGATAGCCCTAAAAC	19	#5_ends	E15
15[343]	15[358]	GCTATTACGCCAGCTGG	17	#5_ends	F15
16[020]	16[005]	AACAGAGGTGAGGCGG	16	#5_ends	E16
16[361]	16[348]	TGCGCAACTGTTGGG	15	#5_ends	F16
17[005]	17[020]	TCAGTATTAACACCGC	16	#5_ends	E17
17[343]	17[361]	CGCCATTCGCCATTCAGGC	19	#5_ends	F17
18[020]	18[005]	CAGCAGCAAATGAAAA	16	#5_ends	E18
18[361]	18[343]	CACTCCAGCCAGCTTTCCG	19	#5_ends	F18
19[005]	19[020]	ATCTAAAGCATCACCT	16	#5_ends	E19
19[348]	19[361]	CTCAGGAAGATCG	13	#5_ends	F19

Supplementary Notes – List of functional DNA staples

1. Functional staples for Origami H

Bottom positions

oligoname	Sequence	Description	Partner staples
B_00	TTATCAGATGATCGGGACT	unmodified staples	
B_01	CTTAATTGAGATCCTGAAC		
B_02	CTTACCGAAGCAAACGCATT		
B_03	CGTCATACATTAGCGGCA		
B_04	CAGCAGCGAAAAGCGATTG		
B_05	AAAAGAAGTTTAGCGGATAA		
B_06	AGGGTGAGAAAATGTCAAG		
B18_00	TTATCAGATGATCGGGACT**GCTATGGGTGGTCTGGTT**	staples with extension for TEG-Chol(18)	*5'-Chol-TEG-***AACCAGACCACCCATAGC**
B18_01	CTTAATTGAGATCCTGAAC**GCTATGGGTGGTCTGGTT**		
B18_02	CTTACCGAAGCAAACGCATT**GCTATGGGTGGTCTGGTT**		
B18_03	CGTCATACATTAGCGGCA**GCTATGGGTGGTCTGGTT**		**AACCAGACCACCCATAGC***-TEG-Chol-3'*
B18_04	CAGCAGCGAAAAGCGATTG**GCTATGGGTGGTCTGGTT**		
B18_05	AAAAGAAGTTTAGCGGATAA**GCTATGGGTGGTCTGGTT**		
B18_06	AGGGTGAGAAAATGTCAAG**GCTATGGGTGGTCTGGTT**		
B09_06	AGGGTGAGAAAATGTCAAG**GGTCTGGTT**	staples with extension for TEG-Chol(9)	*5'-Chol-TEG-***AACCAGACC**
B27_03	CGTCATACATTAGCGGCA**GCTATGGGTGGTCTGGTTGGGATTGGC**	staple with extension for TEG-Chol(27)	*5'-Chol-TEG-***GCCAATCCCAACCAGACCACCCATAGC**
BD_00	TTATCAGATGATCGGGACT**AAAAAACACCAAACCC**	staples with extension for Atto488 dye	*5'-Atto488-***GGGTTTGGTGTTTTTT**
BD_01	CTTAATTGAGATCCTGAAC**AAAAAACACCAAACCC**		
BD_02	CTTACCGAAGCAAACGCATT**AAAAAACACCAAACCC**		
BD_03	CGTCATACATTAGCGGCA**AAAAAACACCAAACCC**		
BD_04	CAGCAGCGAAAAGCGATTG**AAAAAACACCAAACCC**		
BD_05	AAAAGAAGTTTAGCGGATAA**AAAAAACACCAAACCC**		
BD_06	AGGGTGAGAAAATGTCAAG**AAAAAACACCAAACCC**		
B_00_Biot	TTATCAGATGATCGGGACT-**TEG-Biotin-3'**	biotinylated staples	
B_03_Biot	CGTCATACATTAGCGGCA-**TEG-Biotin-3'**		
B_06_Biot	AGGGTGAGAAAATGTCAAG-**TEG-Biotin-3'**		
B_00_Thio	TTATCAGATGATCGGGACT-**C3-SS-3'**	thiolated staples	
B_03_Thio	CGTCATACATTAGCGGCA-**C3-SS-3'**		
B_06_Thio	AGGGTGAGAAAATGTCAAG-**C3-SS-3'**		

Top positions

oligoname	Sequence	Description	Partner staples
T_00	GTTTGAATACGAAGGAGCGGAA	unmodified staples	
T_01	ATTACGAGCAACCAGTATAAAGC		
T_02	GCACAATCGAGCAAGAAACAA		
T_03	CAGCCGTCGAAGCGCAGTCTCTG		
T_04	TCGAGATTTGTTAAAGGCCGCTT		
T_05	CAAAAGACTGATAAAAACCAAA		
T_06	ACAAAGCCCCTGCCTGAGTAATG		
TD_00	GTTTGAATACGAAGGAGCGGAA**AAAAAACACCAAACCC**	staples with extension for At488 dye	*5'-Atto488-***GGGTTTGGTGTTTTTT**
TD_01	ATTACGAGCAACCAGTATAAAGC**AAAAAACACCAAACCC**		
TD_02	GCACAATCGAGCAAGAAACAA**AAAAAACACCAAACCC**		
TD_03	CAGCCGTCGAAGCGCAGTCTCTG**AAAAAACACCAAACCC**		
TD_04	TCGAGATTTGTTAAAGGCCGCTT**AAAAAACACCAAACCC**		
TD_05	CAAAAGACTGATAAAAACCAAA**AAAAAACACCAAACCC**		
TD_06	ACAAAGCCCCTGCCTGAGTAATG**AAAAAACACCAAACCC**		
T18_00	GTTTGAATACGAAGGAGCGGAA**GCTATGGGTGGTCTGGTT**	staples with extension for TEG-Chol(18)	*5'-Chol-TEG-***AACCAGACCACCCATAGC**
T18_03	CAGCCGTCGAAGCGCAGTCTCTG**GCTATGGGTGGTCTGGTT**		**AACCAGACCACCCATAGC***-TEG-Chol-3'*
T18_06	ACAAAGCCCCTGCCTGAGTAATG**GCTATGGGTGGTCTGGTT**		

Edge positions

oligoname	Sequence	Description	Partner staples
E_06	TAGTAATAACATCACTTGC**AAAAAACACCAAACCC**	staples with extension for Al488 dye	*5'-Alexa488-***GGGTTTGGTGTTTTTT**
E_13	**CCCAAACCACAAAAAA**TTCTGACCTGAAAGCGTAA		**TTTTTTGTGGTTTGGG**-Alexa488-3'
F_06	**CCCAAACCACAAAAAA**CCTGGGGTGCCTAATG		
F_13	GCCAGGGTTTTCCCAG**AAAAAACACCAAACCC**		

2. Functional staples for Origami Q

Bottom positions

oligoname	Sequence	Description	Partner staples
B_00	ATTCCTGATTACTTTTACCT	unmodified staples	
B_01	AGTAGGGCTTAAAGATAATC		
B_02	AGCTATCTTACCAAAAGAATA		
B_03	GCGTCATACATAGCGGGCC		
B_04	CCTCAGCAGCGACCAGCGCT		
B_05	CAAAAGAAGTTAAGCGGACA		
B_06	GTGAGAAAGGCCAATCATCA		
B18_00	ATTCCTGATTACTTTTACCT**GCTATGGGTGGTCTGGTT**	staples with extension for TEG-Chol(18)	*5'-Chol-TEG-***AACCAGACCACCCATAGC**
B18_01	AGTAGGGCTTAAAGATAATC**GCTATGGGTGGTCTGGTT**		**AACCAGACCACCCATAGC***-TEG-Chol-3'*
B18_02	AGCTATCTTACCAAAAGAATA**GCTATGGGTGGTCTGGTT**		
B18_03	GCGTCATACATAGCGGGCC**GCTATGGGTGGTCTGGTT**		
B18_04	CCTCAGCAGCGACCAGCGCT**GCTATGGGTGGTCTGGTT**		
B18_05	CAAAAGAAGTTAAGCGGACA**GCTATGGGTGGTCTGGTT**		
B18_06	GTGAGAAAGGCCAATCATCA**GCTATGGGTGGTCTGGTT**		
B09_00	ATTCCTGATTACTTTTACCT**GGTCTGGTT**	staples with extension for TEG-Chol(9)	*5'-Chol-TEG-***AACCAGACC**
B09_03	GCGTCATACATAGCGGGCC**GGTCTGGTT**		
B09_06	GTGAGAAAGGCCAATCATCA**GGTCTGGTT**		
B27_03	GCGTCATACATAGCGGGCC**GCTATGGGTGGTCTGGTTGGGATTGGC**	staple with extension for TEG-Chol(27)	*5'-Chol-TEG-***GCCAATCCCAACCAGACCACCCATAGC**
BD_00	ATTCCTGATTACTTTTACCT**AAAAAACACCAAACCC**	staples with extension for Atto488 dye	*5'-Atto488-***GGGTTTGGTGTTTTTT**
BD_01	AGTAGGGCTTAAAGATAATC**AAAAAACACCAAACCC**		
BD_02	AGCTATCTTACCAAAAGAATA**AAAAAACACCAAACCC**		
BD_03	GCGTCATACATAGCGGGCC**AAAAAACACCAAACCC**		
BD_04	CCTCAGCAGCGACCAGCGCT**AAAAAACACCAAACCC**		
BD_05	CAAAAGAAGTTAAGCGGACA**AAAAAACACCAAACCC**		
BD_06	GTGAGAAAGGCCAATCATCA**AAAAAACACCAAACCC**		

Top positions

oligoname	Sequence	Description	Partner staples
T_00	TAGATTGCTAACCACCAGAAG	unmodified staples	
T_01	AACCTAATTTATTCTTACCAGT		
T_02	CTTTGTCAAATAAGAGCAAG		
T_03	ACCCGTCGAGAGCGCAGTCTCT		
T_04	CTCGGAGATTAGTTAAAGGCCG		
T_05	ACGAAAGACCGATAAAAACCAA		
T_06	AACCCAAAAATGAGTAATGTGT		
TD_00	TAGATTGCTAACCACCAGAAG**AAAAAACACCAAACCC**	staples with extension for At488 dye	*5'-Atto488-***GGGTTTGGTGTTTTTT**
TD_01	AACCTAATTTATTCTTACCAGT**AAAAAACACCAAACCC**		
TD_02	CTTTGTCAAATAAGAGCAAG**AAAAAACACCAAACCC**		
TD_03	ACCCGTCGAGAGCGCAGTCTCT**AAAAAACACCAAACCC**		
TD_04	CTCGGAGATTAGTTAAAGGCCG**AAAAAACACCAAACCC**		
TD_05	ACGAAAGACCGATAAAAACCAA**AAAAAACACCAAACCC**		
TD_06	AACCCAAAAATGAGTAATGTGT**AAAAAACACCAAACCC**		
T18_00	TAGATTGCTAACCACCAGAAG**GCTATGGGTGGTCTGGTT**	staples with extension for TEG-Chol(18)	*5'-Chol-TEG-***AACCAGACCACCCATAGC**
T18_03	ACCCGTCGAGAGCGCAGTCTCT**GCTATGGGTGGTCTGGTT**		**AACCAGACCACCCATAGC***-TEG-Chol-3'*
T18_06	AACCCAAAAATGAGTAATGTGT**GCTATGGGTGGTCTGGTT**		
T09_00	TAGATTGCTAACCACCAGAAG**GGTCTGGTT**	staples with extension for TEG-Chol(9)	*5'-Chol-TEG-***AACCAGACC**
T09_03	ACCCGTCGAGAGCGCAGTCTCT**GGTCTGGTT**		
T09_06	AACCCAAAAATGAGTAATGTGT**GGTCTGGTT**		

Lateral positions (left side)

oligoname	Sequence	Description	Partner staples
L_00	ATCGACAACTGAGGATTTAGA	unmodified staples	
L_01	ATAGATGATGGCGAATTATTC		
L_02	TTGATAAATGACCTAAATTTA		
L_03	GGAAACCAAGGCTGTCTTTCC		
L_04	AAAACTGAACATAAAAACAG		
L_05	GAGAGGGAGAGCGCCAAAGAC		
L_06	TAGCATTGAAGCCGCCACCA		
L_07	TTACCCTCAGGTGTATCACCG		
L_08	TGCTTGATATATCAGCTTGCT		
L_09	TGGAGGCGCAATCCGCGACC		
L_10	GAAACTAATAGATTCATCAGT		
L_11	TTCAGGATTAAAGCGAACCA		
L_12	AATGTAATAAAGCTAAATCGG		
L_13	GTAAATTTTATTGTAAACGTT		
LS_00	**TATATATTT**ATCGACAACTGAGGATTTAGA	staples for lateral oligomerization	
LS_01	**TATATATTT**ATAGATGATGGCGAATTATTC		
LS_02	**TATATATTT**TTGATAAATGACCTAAATTTA		
LS_03	**TATATATTT**GGAAACCAAGGCTGTCTTTCC		
LS_04	**TATATATTT**AAAACTGAACATAAAAACAG		
LS_05	**TATATATTT**GAGAGGGAGAGCGCCAAAGAC		
LS_06	**TATATATTT**TAGCATTGAAGCCGCCACCA		
LS_07	**TATATATTT**TTACCCTCAGGTGTATCACCG		
LS_08	**TATATATTT**TGCTTGATATATCAGCTTGCT		
LS_09	**TATATATTT**TGGAGGCGCAATCCGCGACC		
LS_10	**TATATATTT**GAAACTAATAGATTCATCAGT		
LS_11	**TATATATTT**TTCAGGATTAAAGCGAACCA		
LS_12	**TATATATTT**AATGTAATAAAGCTAAATCGG		
LS_13	**TATATATTT**GTAAATTTTATTGTAAACGTT		

Lateral positions (right side)

oligoname	Sequence	Description	Partner staples
R_00	ATATCTGGTCAGACCATATAG	unmodified staples	
R_01	ATTTTCAGGTTTCAATAGTCC		
R_02	TCCGGCTTAGGTAAAGTACAC		
R_03	AACATGTTCAGCTACAATTAG		
R_04	CCTAATTTGCCAGGGAATACGT		
R_05	TAGCAAACGTAGCAGACTGTT		
R_06	AGCGTTTGCCATCACAGTTAAA		
R_07	AGTATTAAGAGGTAGTAAATT		
R_08	TCAGCGGAGTGAATTAAACAA		
R_09	CGAAAGAGGCAAAACGAGTATG		
R_10	TGAATTACCTTAATCCCCCAA		
R_11	TCAAAAATCAGGTCATTTCGCG		
R_12	CGAGCTGAAAAGTTTGAGACA		
R_13	AACAAGAGAATCATCTGCCCC		
RS_00	**TATATATTT**ATATCTGGTCAGACCATATAG	staples for lateral oligomerization	
RS_01	**TATATATTT**ATTTTCAGGTTTCAATAGTCC		
RS_02	**TATATATTT**TCCGGCTTAGGTAAAGTACAC		
RS_03	**TATATATTT**AACATGTTCAGCTACAATTAG		
RS_04	**TATATATTT**CCTAATTTGCCAGGGAATACGT		
RS_05	**TATATATTT**TAGCAAACGTAGCAGACTGTT		
RS_06	**TATATATTT**AGCGTTTGCCATCACAGTTAAA		
RS_07	**TATATATTT**AGTATTAAGAGGTAGTAAATT		
RS_08	**TATATATTT**TCAGCGGAGTGAATTAAACAA		
RS_09	**TATATATTT**CGAAAGAGGCAAAACGAGTATG		
RS_10	**TATATATTT**TGAATTACCTTAATCCCCCAA		
RS_11	**TATATATTT**TCAAAAATCAGGTCATTTCGCG		
RS_12	**TATATATTT**CGAGCTGAAAAGTTTGAGACA		
RS_13	**TATATATTT**AACAAGAGAATCATCTGCCCC		
R18_00	ATATCTGGTCAGACCATATAG**GCTATGGGTGGTCTGGTT**	staples with extension for TEG-Chol(18)	*5'-Chol-TEG-***AACCAGACCACCCATAGC**
R18_06	AGCGTTTGCCATCACAGTTAAA**GCTATGGGTGGTCTGGTT**		**AACCAGACCACCCATAGC***-TEG-Chol-3'*
R18_12	CGAGCTGAAAAGTTTGAGACA**GCTATGGGTGGTCTGGTT**		

Edge positions

oligoname	Sequence	Description	Partner staples
E_08	TTACCGCCAGCCATTG	staples for tip-to-tip interactions:	
E_09	CAACAGGAAAAACGCT	- Q-E5 structures (10 of 10)	
E_10	CTCAATCGTCTGAAAT	- Q-E7 structures (10 of 14)	
E_14	TGAATGGCTATTAGTCTTT	- Q-E13 structures (10 of 26)	
E_18	GCTGAGAGCCAGCAGC		
F_08	TCCGCTCACAATTC		
F_09	TTCCTGTGTGAAATTGTTA		
F_10	TAGAGGATCCCCGGGTACC		
F_14	GGGGGATGTGCTGCAA		
F_18	GCCAGCTTTCCGGCACCGC		
E_04	TTTATAATCAGTGAGGCCA	additional staples:	
E_19	AAATGAAAAATCTAAA	- Q-E7 structures (4 of 14)	
F_04	CCAGTCGGGAAACCTG	- Q-E13 structures (4 of 26)	
F_19	AAGATCGCACTCCA		
E_03	CGATTAAAGGGATTTT	additional staples:	
E_05	CCGAGTAAAAGAGTCTGTC	- Q-E13 structures (12 of 26)	
E_06	CTTTGATTAGTAATAACAT		
E_07	CACTTGCCTGAGTAGAAGA		
E_11	GGATTATTTACATTGG		
E_15	AATGCGCGAACTGATAGCC		
F_03	AATCGGCCAACGCGC		
F_05	GCTCACTGCCCGCTTT		
F_06	GGTGCCTAATGAGTGA		
F_07	AAAGTGTAAAGCCTGG		
F_11	CTGCAGGTCGACTC		
F_15	ACGCCAGCTGGCGAAA		

3. Functional staples for Origami L

Bottom positions

oligoname	Sequence	Description	Partner staples
B_00	ATTATCATCATAAACAGTATG	unmodified staples	
B_01	GCTCAACAGTAGCAACAATCG		
B_02	GCAATAGCTATCCATATAAAC		
B_03	GTAAGCGTCATGATTAGCAC		
B_04	TCAGCAGCGAAAAGCGATTGA		
B_05	AGAAGTTTTGCCGATTGCAAA		
B_06	AAGGCCGGAGACATGTACCTC		
B18_00	ATTATCATCATAAACAGTATG**GCTATGGGTGGTCTGGTT**	staples with extension for TEG-Chol(18)	*5'-Chol-TEG-***AACCAGACCACCCATAGC**
B18_03	GTAAGCGTCATGATTAGCAC**GCTATGGGTGGTCTGGTT**		
B18_06	AAGGCCGGAGACATGTACCTC**GCTATGGGTGGTCTGGTT**		

Top positions

oligoname	Sequence	Description	Partner staples
T_00	AAATTCGCCCGGAACAAAGAA	unmodified staples	
T_01	ATTCCCATCTATACAAATTCT		
T_02	ATTTATTTCCAATAATAAGA		
T_03	AAGTGCCGTGGAAAGCGCAGT		
T_04	CAAGATTTGTTAAAGGCCGCT		
T_05	TTACTTCAAAAAACCAAAATA		
T_06	AGACAGGAAATGTGTAGGTAA		
TD_00	AAATTCGCCCGGAACAAAGAA**AAAAAAACACCAAACCC**	staples with extension for At488 dye	*5'-Atto488-***GGGTTTGGTGTTTTTT**
TD_01	ATTCCCATCTATACAAATTCT**AAAAAAACACCAAACCC**		
TD_02	ATTTATTTCCAATAATAAGA**AAAAAAACACCAAACCC**		
TD_03	AAGTGCCGTGGAAAGCGCAGT**AAAAAAACACCAAACCC**		
TD_04	CAAGATTTGTTAAAGGCCGCT**AAAAAAACACCAAACCC**		
TD_05	TTACTTCAAAAAACCAAAATA**AAAAAAACACCAAACCC**		
TD_06	AGACAGGAAATGTGTAGGTAA**AAAAAAACACCAAACCC**		
