# Supplementary material for: Membrane sculpting by curved DNA origami scaffolds
Source: Nat Commun. 2018 Feb 23;9:811. doi: 10.1038/s41467-018-03198-9 (PMC5824810; doi:10.1038/s41467-018-03198-9)
Supplement: Supplementary file 3 — Description of Additional Supplementary Files [file 41467_2018_3198_MOESM3_ESM.pdf]

Description of Additional Supplementary Files

File Name: Supplementary Movie 1
Description: Minor shrinkage upon hypertonic deflation (t = 0 s) of giant unilamellar vesicles (labeled with DOPEAtto655, red) pre-incubated with the non-membrane-binding origami structure Q0 (labeled with Atto488, green). Scale bar: 10µm.

File Name: Supplementary Movie 2
Description: Outward tubule formation upon hyperosmotic stress (t = 0 s) of giant unilamellar vesicles (labeled with DOPE-Atto655, red) pre-incubated with membrane-bound concave origami Q3 (labeled with Atto488, green). Scale bar: 10µm.

File Name: Supplementary Movie 3
Description: Minor shrinkage upon hypertonic deflation (t = 0 s) of giant unilamellar vesicles (labeled with DOPEAtto655, red) pre-incubated with Atto488-labeled (green) origami structure QR3 (membrane binding side with zero curvature) Scale bar: 10µm.

File Name: Supplementary Movie 4
Description: Membrane deformations upon hyperosmotic stress (t = 0 s) of giant unilamellar vesicles (labeled with DOPE-Atto655, red) pre-incubated with membrane-bound convex origami QI3 (labeled with Atto488, green). Scale bar: 10µm
