# Supplementary material for: Membrane sculpting by curved DNA origami scaffolds
Source: Nat Commun. 2018 Feb 23;9:811. doi: 10.1038/s41467-018-03198-9 (PMC5824810; doi:10.1038/s41467-018-03198-9)
Supplement: Supplementary file 2 — Peer Review File [file 41467_2018_3198_MOESM2_ESM.pdf]

Editorial Note: This manuscript has been previously reviewed at another journal that is not operating a transparent peer review scheme. The manuscript was considered suitable for publication without further review at *Nature Communications.*
